# Validation of the SMART-REACH model after stroke and the effect of colchicine by atherosclerotic cardiovascular disease risk category: a secondary analysis of the CONVINCE randomised clinical trial

**DOI:** 10.1093/esj/aakag033

**Published:** 2026-04-25

**Authors:** Louise Maes, Claudia Verschuere, Cathal Walsh, Christian Weimar, Francisco Purroy, Christopher Price, Catarina Fonseca, Michael D Hill, Dalius Jatuzis, Janika Kõrv, Christina Kruuse, Robert Mikulik, Paul Nederkoorn, Anna Czlonkowska, Urs Fischer, Michael McCormick, Maria Castellanos, Miguel Baptista, João Pedro Marto, Kamy Thavanesan, David J Williams, Peter Kelly, Robin Lemmens, for the CONVINCE investigators, G Vanhooren, D Hemelsoet, A Peeters, L Sinnaeve, M Hill, M Haršány, O Škoda, H Klingenberg Iversen, C Kruuse, N Solokha, J Korv, P Kraft, T Neumann-Haefelin, H Soda, K G Häusler, B Schmitz, G Thomalla, C Krogias, S Murphy, S Cronin, R Collins, D McCabe, D Williams, J Harbison, E Dolan, P Cotter, P Hickey, P Bienfait, A Członkowska, W Brola, M Baptista, J Marto, E Cuadrado, M Castellanos, D Cánovas, M Medina, J Masjuan, M Gomez-Choco, M J Serena, S Perez Sanchez, J Fernandez Dominguez, M Heldner, M Barber, N Evans, G Shim, L Sztriha, K Adie, A Ali, M Garside, L D’Anna, L Sekaran, A Bhalla, B Keegan, K Rashed, T England, G Gunathilagan, S Ivatts, P Guyler, D Lashley, A Elmerimi, M Hasan, T Hlaing, M Burn

**Affiliations:** Department of Neurology, University Hospitals Leuven, Leuven, Belgium; Department of Neurosciences, Experimental Neurology, KU Leuven - University of Leuven, Leuven, Belgium; Department of Neurology, University Hospitals Leuven, Leuven, Belgium; TCD Biostatistics Unit, Discipline of Public Health and Primary Care, School of Medicine, Trinity College Dublin, Dublin, Ireland; BDH-Clinic Elzach, Elzach, Germany; Institute for Medical Informatics, Biometry and Epidemiology, University Hospital, University Duisburg-Essen, Essen, Germany; Stroke Unit, Department of Neurology, Hospital Universitari Arnau de Vilanova de Lleida, Lleida, Spain; Biomedical Research Institute of Lleida (IRBLleida), Universitat de Lleida, Lleida, Spain; Population Health Sciences Institute, Newcastle University, Newcastle, United Kingdom; Neurology Department, Hospital Santa Maria, Faculdade de Medicina, Universidade de Lisboa, Lisbon, Portugal; Department of Clinical Neurosciences & Hotchkiss Brain Institute, Cumming School of Medicine, University of Calgary and Foothills Medical Centre, Calgary, Alberta, Canada; Centre of Neurology, Institute of Clinical Medicine, Faculty of Medicine, Vilnius University, Vilnius, Lithuania; Department of Neurology and Neurosurgery, Institute of Clinical Medicine, University of Tartu, Tartu, Estonia; Department of Neurology, Copenhagen University Hospital -Herlev and Gentofte, and Department of Brain and Spinal Cord Injury, Copenhagen University Hospital -Rigshospitalet, Copenhagen, Denmark; International Clinical Research Center and Department of Neurology, St Anne’s University Hospital and Masaryk University Brno, Brno, Czech Republic; Department of Neurology, Amsterdam University Medical Center, Amsterdam, The Netherlands; 2nd Department of Neurology, Institute of Psychiatry and Neurology, Warsaw, Poland; Department of Neurology, University Hospital Bern and University of Bern, Bern, Switzerland; Stroke Service, Craigavon Area Hospital, Portadown, United Kingdom; Neurology Department, A Coruña University Hospital Complex and Biomedical Research Institute, A Coruña, Spain; Department of Physiotherapy, Medicine, and Biomedical Sciences, University of A Coruña, A Coruña, Spain; Department of Neurology, Hospital Egas Moniz, ULS Lisboa Ocidental, Lisbon, Portugal; NOVA Medical School, NOVA University, Lisbon, Portugal; Department of Neurology, Hospital Egas Moniz, ULS Lisboa Ocidental, Lisbon, Portugal; NOVA Medical School, NOVA University, Lisbon, Portugal; Department of Stroke Medicine, Royal Bournemouth and Christchurch Hospital, University Hospitals Dorset NHS Trust, Bournemouth, United Kingdom; Health Research Board (HRB) Stroke Clinical Trials Network Ireland (SCTNI), University College Dublin, Dublin, Ireland; RCSI University of Medicine and Health Sciences and Beaumont Hospital, Dublin, Ireland; Health Research Board (HRB) Stroke Clinical Trials Network Ireland (SCTNI), University College Dublin, Dublin, Ireland; School of Medicine, University College Dublin (UCD), Dublin, Ireland; Stroke Service, Mater Misericordiae University Hospital, Dublin, Ireland; Department of Neurology, University Hospitals Leuven, Leuven, Belgium; Department of Neurosciences, Experimental Neurology, KU Leuven - University of Leuven, Leuven, Belgium

**Keywords:** colchicine, stroke, transient ischaemic attack, atherosclerotic cardiovascular disease, risk factors, risk estimation, secondary prevention

## Abstract

**Introduction:**

The Colchicine for prevention of vascular inflammation in Non-CardioEmbolic stroke (CONVINCE) trial showed that recurrent events were significantly reduced among colchicine-adherent non-cardioembolic stroke patients in the on-treatment analysis. This study aimed to validate the SMART-REACH risk score in stroke patients, and to determine whether colchicine’s efficacy varies by baseline atherosclerotic cardiovascular disease (ASCVD) risk.

**Patients and methods:**

Patients with non-severe non-cardioembolic ischaemic stroke/transient ischaemic attack (TIA) were randomised to colchicine 0.5 mg plus usual care or usual care alone. Participants were stratified into moderate (10%–19%), high (20%–30%) and very high (≥30%) 10-year ASCVD risk categories using the SMART-REACH model. Model performance was assessed using the C-statistic and calibration plots. The primary endpoint (major adverse cardiovascular events [MACE]) was a composite of fatal or non-fatal recurrent ischaemic stroke, myocardial infarction, cardiac arrest or hospitalisation for unstable angina.

**Results:**

Among 3144 patients, MACE incidence significantly increased with ASCVD risk levels: 7.2% (moderate), 8.8% (high) and 13.8% (very high) (*P* < .01). The C-statistic for 3-year risk of MACE was 0.59 (95% CI, 0.56–0.63). While no statistically significant treatment interaction was found (*P* = .88), absolute risk reductions (ARRs) were more pronounced in higher-risk groups: moderate risk 7.2% (colchicine) vs 7.2% (usual care) (hazard ratio [HR] 1.01; 95% CI, 0.55–1.83); high risk 7.7% vs 9.8% (ARR 2.1%; HR 0.79; 95% CI, 0.53–1.18); very high risk 12.5% vs 15.2% (ARR 2.7%; HR 0.85; 95% CI, 0.64–1.12).

**Discussion and Conclusion:**

We identified an association between very high baseline ASCVD risk (≥30%) assigned by the SMART-REACH score and increased recurrent MACE. Although no significant treatment interaction was observed, patients in higher risk categories may represent a more promising target population for secondary prevention with colchicine.

**Trial Registration:**

ClinicalTrials.gov Identifier: NCT02898610.

**Graphical Abstract:**
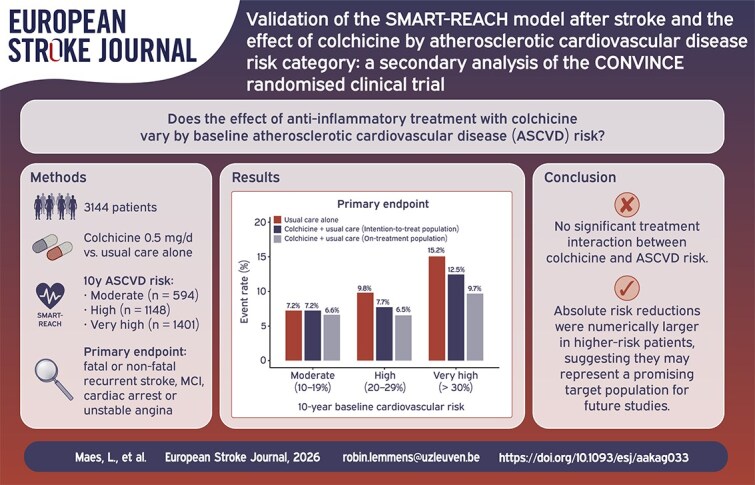

## Introduction

Cardiovascular disease remains the leading cause of death worldwide.^1^ Patients with established atherosclerotic cardiovascular disease (ASCVD) are at high risk for recurrent events. Although risk factor modification results in improved survival, a substantial residual risk persists in patients with ASCVD.^2^ Recurrent events are likely higher in individuals with multiple ASCVD risk factors. Therefore, a treatment response to secondary preventive medication might depend on baseline ASCVD risk profile. The SMART and SMART-REACH risk models are recommended by European Society of Cardiology (ESC) guidelines for prediction of 10-year and lifetime risk of myocardial infarction, stroke or cardiovascular death in patients with established ASCVD;^3–5^ however, these models have not been well-validated following stroke.

Targeting inflammation can provide an important therapeutic option in patients with cardiovascular disease. Recently, both European and American guidelines have recommended low-dose colchicine in selected patients with atherosclerotic coronary artery disease.^6^^,^^7^ Despite encouraging findings in coronary artery disease, the role of colchicine on long-term secondary stroke prevention remains to be robustly established. The only completed long-term prevention trial, the Colchicine for prevention of vascular inflammation in Non-CardioEmbolic stroke (CONVINCE) trial, evaluated colchicine for the prevention of major adverse cardiovascular events (MACE) after ischaemic stroke.^8^ Although underpowered due to the COVID-19 pandemic, the direction of effect favoured colchicine, with fewer outcome events in the colchicine-treated group. Moreover, in the on-treatment analysis, a significant benefit was observed in patients compliant with colchicine therapy. In a study estimating the long-term benefit of colchicine in patients with chronic coronary artery disease, the greatest benefit was observed in those with higher baseline ASCVD risk, as defined by the SMART-REACH model.^9^

The primary objective of this secondary analysis of the CONVINCE trial was to validate the SMART-REACH risk model in a population with ischaemic stroke and long-term follow-up. The secondary objective was to evaluate whether colchicine provided greater benefit in reducing recurrent MACE and stroke among patients with higher baseline ASCVD risk.

## Patients and methods

### Study population

CONVINCE was an investigator-led, parallel-group, prospective, randomised open-label, blinded-endpoint-assessed controlled phase 3 trial in which participants were randomised to receive low-dose colchicine (0.5 mg daily) in addition to usual care, or to usual care alone. Patients were eligible if they were aged 40 years or older with non-severe ischaemic stroke (mRS score ≤ 3) or high-risk transient ischaemic attack (TIA) (ABCD2 score ≥ 4), for whom the qualifying event was most likely caused by large artery atherosclerosis of an ipsilateral carotid, vertebral, or intracranial artery, by lacunar disease or by cryptogenic embolism after assessment by the treating clinicians. Patients were ineligible if the qualifying stroke or transient ischaemic attack was likely caused by atrial fibrillation, other cardiac embolism or other defined causes such as arterial dissection. Detailed descriptions of methodology and primary results of the trial have been published elsewhere.^8^^,^^10^ The study protocol and statistical analysis plan are available as supplemental material. In this predefined secondary analysis, all consenting randomised patients were included. All findings are reported following the Consolidated Standards of Reporting Trials (CONSORT) guidelines.

### SMART-REACH model

The SMART2 and SMART-REACH risk models are recommended by the ESC guidelines for prediction of 10-year and lifetime risk of myocardial infarction, stroke or cardiovascular death (MACE) and MACE-free life expectancy in patients with established coronary, cerebrovascular and/or peripheral artery disease.^3–5^ For the current analysis, the SMART-REACH model was used to predict 10-year ASCVD risk. The following predictors are used in the model: sex, current smoking, diabetes mellitus, systolic blood pressure, total cholesterol, creatinine, number of cardiovascular disease locations, atrial fibrillation, heart failure and antithrombotic therapy. All variables included as predictors were available in the CONVINCE database. Participants were classified according to the predicted 10-year cardiovascular event risk into the following categories: low risk (<10%), moderate risk (10%–20%), high risk (>20%–30%) and very high risk (≥30%).^4^ These thresholds were chosen to align with prior studies applying SMART2 and SMART-REACH models in populations with established cardiovascular disease.^9^^,^^11^^,^^12^

### Outcomes

The primary outcome was a composite primary endpoint of first recurrent non-fatal ischaemic stroke, myocardial infarction, cardiac arrest, hospitalisation for unstable angina or vascular death. Secondary outcomes included all ischaemic stroke (fatal and non-fatal).

### Statistical analysis

Continuous variables were presented as medians with interquartile ranges or means with standard deviations, and categorical variables were presented as frequencies and percentages. Baseline characteristics were compared between ASCVD risk groups using χ^2^ tests for categorical variables, and the Kruskal–Wallis test for continuous variables.

The SMART-REACH risk model was externally validated in CONVINCE. Model performance was assessed for discrimination (using the c-statistic) and calibration (using plots of the predicted vs observed 3-year risk) across deciles of predicted risk.

Statistical analyses were performed in the intention-to-treat, as well as the on-treatment populations. The on-treatment population was defined as all randomised patients who took at least one dose of colchicine with censoring at last reported compliance date in those who permanently discontinued colchicine.

First, a Cox proportional hazards model was used to test if the treatment effect of colchicine was modified by ASCVD risk group by including a multiplicative interaction term in the model. Next, separate Cox proportional hazards models were performed within each ASCVD risk group, to explore potential differences in efficacy of colchicine based on ASCVD risk. In a sensitivity analysis, the moderate and high-risk groups were combined, and compared with the very high-risk group. Hazard ratios (HRs) and 95% CIs were calculated from Cox regression models adjusted for age, time since entry event and type of qualifying event (TIA/stroke).

Missing baseline characteristics (≤0.01% for all predictor variables) were imputed using the median of the respective variable in the complete dataset. Age values were constrained to the model’s applicable range: patients younger than 45 years had their age set to 47.5, while those older than 80 years had their age set to 78.5.

All statistical analyses were conducted in R (version 4.4.2, 2024-03-06).

## Results

### Patient characteristics

**Figure 1:**
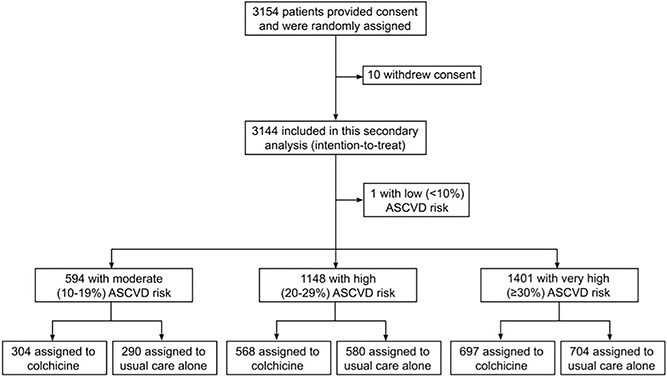
CONSORT flowchart of patient selection. Abbreviation: CONSORT = Consolidated Standards of Reporting Trials

In this analysis, we included 3143 patients out of 3154 enrolled in CONVINCE (Figure 1). Ten participants were excluded due to withdrawal of consent. A single patient was classified with low (<10%) ASCVD risk, which precluded meaningful analysis, and was therefore excluded from further analysis.

In the remaining 3143 patients, 594 (18.9%) were classified with moderate (10%–19%) ASCVD risk, 1148 (36.5%) with high (20%–29%) ASCVD risk and 1401 (44.6%) with very high (≥30%) ASCVD risk. Table 1 shows the baseline characteristics stratified by ASCVD risk group and treatment arm. The prevalence of variables included in the SMART-REACH risk model increased in the higher risk scores (see also Table S1). TIA as the qualifying event for inclusion was more frequent with increasing ASCVD risk (*P* < .01). Baseline C-reactive protein (CRP) levels did not differ between risk groups (*P* = .42).

**Table 1:** Baseline characteristics according to ASCVD risk.

	Moderate (10%–19%) risk			High (20%–29%) risk		Very high (≥30%) risk			
	Total (*n* = 594)	Colchicine (*n* = 304)	Control (*n* = 290)	Total (*n* = 1148)	Colchicine (*n* = 568)	Control (*n* = 580)	Total (*n* = 1401)	Colchicine (*n* = 697)	Control (*n* = 704)
**Age, years**	57 (8.8)	57 (7.2)	57 (8.8)	64 (11)	64 (11)	65 (11)	73 (11)	73 (11)	73 (11)
**Female**	193 (32.5)	99 (32.6)	94 (32.4)	355 (30.9)	185 (32.6)	170 (29.3)	404 (28.8)	204 (29.3)	200 (28.4)
**Race**									
**White**	559 (94.1)	279 (91.8)	280 (96.6)	1086 (94.6)	538 (94.7)	548 (94.5)	1355 (96.7)	677 (97.1)	678 (96.3)
**Black**	18 (3)	13 (4.3)	5 (1.7)	37 (3.2)	17 (3.0)	20 (3.4)	20 (1.4)	6 (0.9)	14 (2.0)
**Asian**	11 (1.9)	7 (2.3)	4 (1.4)	16 (1.4)	10 (1.8)	6 (1.0)	19 (1.4)	10 (1.4)	9 (1.3)
**Other**	6 (1.0)	5 (1.6)	1 (0.3)	9 (0.8)	3 (0.5)	6 (1.0)	7 (0.5)	4 (0.6)	4 (0.4)
**Onset to randomisation, days**	10 (11)	10 (12)	9.5 (11)	9 (12)	8 (10.2)	9 (13)	9 (13)	9 (12)	9 (13)
**Qualifying event**									
**Stroke**	540 (90.9)	277 (91.1)	263 (90.7)	1032 (89.9)	511 (90.0)	521 (89.8)	1192 (85.1)	593 (85.1)	599 (85.1)
**Transient ischaemic attack**	54 (9.1)	27 (8.9)	27 (9.3)	116 (10.1)	57 (10.0)	59 (10.2)	209 (14.9)	104 (14.9)	105 (14.9)
**Modified Rankin Scale score**	1 (2)	1 (2)	1 (2)	1 (2)	1 (2)	1 (2)	1 (2)	1 (2)	1 (2)
**National Institutes of Health Stroke Scale score**	1 (2)	1 (2)	1 (2)	1 (3)	1 (3)	1 (2)	1 (3)	1 (3)	1 (3)
**ABCD2 score (TIA only)**	4 (1)	4 (1)	4 (1)	5 (1)	5 (1)	4 (1)	5 (1)	5 (2)	5 (1)
**Carotid revascularisation at 28 days**	3 (0.5)	2 (0.7)	1 (0.3)	28 (2.4)	14 (2.5)	14 (2.4)	39 (2.8)	22 (3.2)	17 (2.4)
**Emergency treatment**									
**Thrombolysis**	82 (13.8)	46 (15.1)	36 (12.4)	179 (15.6)	82 (14.4)	97 (16.7)	212 (15.2)	128 (18.4)	84 (11.9)
**Thrombectomy**	38 (6.4)	17 (5.6)	21 (7.2)	70 (6.1)	35 (6.2)	35 (6.0)	64 (4.6)	35 (5.0)	29 (4.1)
**Previous stroke**	39 (6.6)	21 (6.9)	18 (6.2)	120 (10.5)	57 (10.0)	63 (10.9)	172 (12.3)	79 (11.3)	93 (13.2)
**Hypertension**	316 (53.2)	159 (52.3)	157 (54.1)	698 (60.8)	348 (61.3)	350 (60.3)	1042 (74.4)	519 (74.5)	523 (74.4)
**Diabetes**	9 (1.5)	8 (2.6)	1 (0.3)	163 (14.2)	86 (15.1)	77 (13.3)	529 (37.8)	264 (37.9)	265 (37.6)
**Smoker**	65 (10.9)	33 (10.9)	32 (11.0)	282 (24.6)	139 (24.5)	143 (24.7)	347 (24.8)	178 (25.5)	169 (24.0)
**Previous coronary artery disease**	4 (0.7)	3 (1.0)	1 (0.3)	42 (3.7)	15 (2.6)	27 (4.7)	233 (16.6)	108 (15.5)	125 (17.8)
**Peripheral artery disease**	2 (0.3)	0 (0.0)	2 (0.7)	16 (1.4)	8 (1.4)	8 (1.4)	110 (7.9)	55 (7.9)	55 (7.8)
**Gout**	12 (2.0)	4 (1.3)	8 (2.8)	31 (2.7)	17 (3.0)	14 (2.4)	73 (5.2)	31 (4.4)	42 (6.0)
**Baseline C-reactive protein, mg/L**	3 (3.8)	2.8 (4)	3 (4.5)	3 (5)	2.8 (5)	3.2 (5)	3 (4.9)	3.4 (4.9)	3 (4.9)
**Medication at randomisation**									
**Any antiplatelet**	590 (99.3)	303 (99.7)	287 (99.0)	1125 (98.0)	555 (97.7)	570 (98.3)	1350 (96.4)	666 (95.6)	684 (97.2)
**Any statin**	558 (93.9)	290 (95.4)	268 (92.4)	1079 (94.0)	529 (93.1)	550 (94.8)	1294 (92.4)	639 (91.7)	655 (93.0)

Abbreviations: ASCVD, atherosclerotic cardiovascular disease; TIA, transient ischaemic attack.

Continuous variables are presented as median (interquartile range), categorical variables as *n*(%).

Variables included in the SMART-REACH model are presented in Table S1.

### Outcome events

We did not identify a significant interaction between treatment assignment and ASCVD risk group for the primary endpoint (*P* for interaction = .88), and therefore we performed all analyses in the full study cohort. In the intention-to-treat population, the primary endpoint occurred in 7.2% of patients (43/594) with moderate ASCVD risk; 8.8% (101/1148) of patients with high ASCVD risk and 13.8% of patients (194/1401) with very high ASCVD risk (*P* < .01). (Table 2). On multivariable Cox regression analysis, compared to the moderate category, the adjusted HR for MACE associated with the very high SMART-REACH risk category was 1.99 (95% CI, 1.37–2.89, *P* < .001). The adjusted HR for the high SMART REACH risk category was 1.18 (95% CI, 0.82–1.70, *P* = .37).

**Table 2:** Outcome events by ASCVD risk.

	Moderate (10%–19%) risk			High (20%–29%) risk			Very high (≥30%) risk		
Intention-to-treat	Total (*n* = 594)	Colchicine (*n* = 304)	Control (*n* = 290)	Total (*n* = 1148)	Colchicine (*n* = 568)	Control (*n* = 580)	Total (*n* = 1401)	Colchicine (*n* = 697)	Control (*n* = 704)
**Primary endpoint**	43 (7.2)	22 (7.2)	21 (7.2)	101 (8.8)	44 (7.7)	57 (9.8)	194 (13.8)	87 (12.5)	107 (15.2)
**All ischaemic stroke (fatal and non-fatal)**	38 (6.4)	20 (6.6)	18 (6.2)	79 (6.9)	34 (6.0)	45 (7.8)	127 (9.1)	54 (7.7)	73 (10.4)
**On-treatment**	**Total** **(*n* = 592)**	**Colchicine** **(*n* = 302)**	**Control** **(*n* = 290)**	**Total** **(*n* = 1147)**	**Colchicine** **(*n* = 567)**	**Control** **(*n* = 580)**	**Total** **(*n* = 1394)**	**Colchicine** **(*n* = 690)**	**Control** **(*n* = 704)**
**Primary endpoint**	41 (6.9)	20 (6.6)	21 (7.2)	94 (8.2)	37 (6.5)	57 (9.8)	174 (12.5)	67 (9.7)	107 (15.2)
**All ischaemic stroke (fatal and non-fatal)**	37 (6.3)	19 (6.3)	18 (6.2)	73 (6.4)	28 (4.9)	45 (7.8)	116 (8.3)	43 (6.2)	73 (10.4)

Abbreviation: ASCVD, atherosclerotic cardiovascular disease.

### External validation of the SMART-REACH risk model

**Figure 2:**
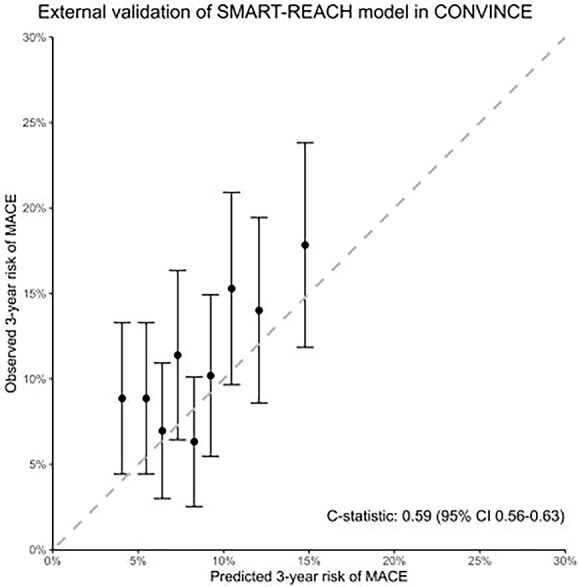
Calibration plot of external validation of the SMART-REACH model in the CONVINCE trial. The calibration plot shows the mean predicted risk (X-axis) against the mean observed risk (Y-axis) of MACE across deciles of predicted risk. Each box represents the mean observed risk for a decile, with vertical lines indicating the 95% CIs of the observed risk. The dotted diagonal line represents perfect calibration, where predicted and observed risks would be identical. Boxes above the diagonal indicate underestimation of risk (observed risk > predicted risk), while boxes below the diagonal indicate overestimation (observed risk < predicted risk). Abbreviations: CONVINCE = Colchicine for prevention of vascular inflammation in Non-CardioEmbolic stroke; MACE = major adverse cardiovascular events.

The SMART-REACH model demonstrated modest predictive performance in the CONVINCE stroke population, with a C-statistic of 0.59 (95% CI, 0.56–0.63), indicating limited discrimination between individuals with and without recurrent MACE. Calibration analysis showed a mean calibration error of 0.004 (0.4%), suggesting adequate overall prediction. However, the calibration plot revealed only moderate agreement between predicted and observed MACE risk across ASCVD risk deciles, showing a systematic underestimation of ASCVD risk in CONVINCE participants (Figure 2).

### Colchicine treatment effect by risk category

**Figure 3:**
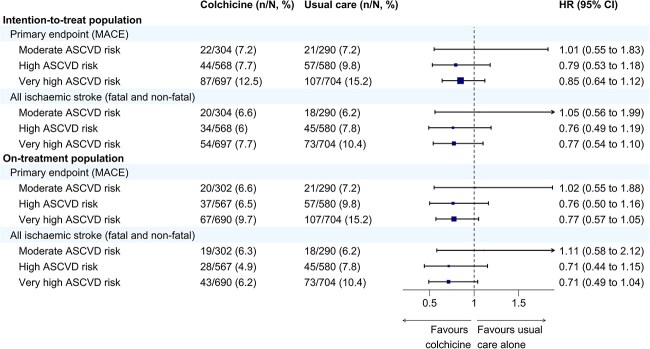
Forest plot of the treatment effect across baseline ASCVD risk groups as estimated by the SMART-REACH model. HRs and 95% CIs are shown for the effect of colchicine vs usual care alone on the prespecified study endpoints, presented for both the intention-to-treat and on-treatment populations. The primary endpoint was a composite of first recurrent non-fatal ischaemic stroke, myocardial infarction, cardiac arrest, hospitalisation for unstable angina or vascular death. Abbreviations: ASCVD = atherosclerotic cardiovascular disease; HRs = hazard ratios.

**Figure 4:**
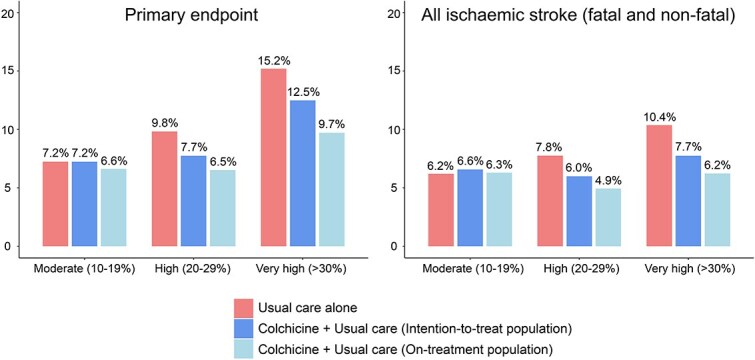
Bar chart showing the event rates of the primary composite endpoint and all ischaemic strokes by baseline ASCVD risk groups, as estimated by the SMART-REACH model, and by treatment assignment. Event rates are presented as percentages for patients receiving usual care alone colchicine in the intention-to-treat population and colchicine in the on-treatment population. The primary composite endpoint included first recurrent non-fatal ischaemic stroke, myocardial infarction, cardiac arrest, hospitalisation for unstable angina or vascular death. Abbreviation: ASCVD = atherosclerotic cardiovascular disease.

In the moderate-risk group, the primary endpoint rates were 7.2% in both the colchicine (22/304) and usual care (21/290) arms (HR 1.01; 95% CI, 0.55–1.83). In the high-risk group, the primary outcome occurred in 7.7% (44/568) colchicine-treated patients compared to 9.8% (57/580) in the usual care (absolute risk reduction [ARR] 2.1%; HR 0.79; 95% CI, 0.53–1.18). In the very high-risk group, event rates were 12.5% (87/697) in the colchicine arm vs 15.2% (107/704) in the usual care arm (ARR 2.7%; HR 0.85; 95% CI, 0.64–1.12) (Figures 3 and 4A).

In the on-treatment analysis, the high-risk and very high-risk groups showed directionally consistent but statistically non-significant absolute and relative risk reductions with colchicine-treatment comparable to the intention-to-treat analysis (Figure 3 and 4A). Similar results were observed for the endpoint of ischaemic stroke (Figure 3 and 4B).

In a sensitivity analysis combining the moderate and high-risk group, the primary outcome occurred in 7.6% (66/872) of the colchicine-treated patients compared to 9.0% (78/870) in the usual care (ARR 1.4%; HR 0.85; 95% CI, 0.61–1.17). In the very high-risk group, event rates were 12.5% (87/697) in the colchicine arm vs 15.2% (107/704) in the usual care arm (ARR 2.7%; HR 0.85; 95% CI, 0.64–1.12). There was no significant interaction (*P* = .88).

## Discussion

In this sub-study of the CONVINCE trial, we externally validated the SMART-REACH model, and studied the efficacy of colchicine in patients with stroke or TIA, stratified by baseline ASCVD risk. We identified an association between very high baseline ASCVD risk (≥30%) assigned via the SMART-REACH score and increased recurrent MACE events. Although no statistically significant interaction between ASCVD risk group and treatment effect of colchicine was observed, we noted a trend toward greater risk reductions in the high and very high ASCVD risk groups. These findings should be interpreted as exploratory and hypothesis generating.

Following stroke, patients with multiple ASCVD risk factors are more likely to experience recurrent events. Treatment response might therefore depend on patient characteristics related to their baseline ASCVD profile, as estimated by the SMART-REACH risk model.^3–5^ The SMART-REACH model was originally developed in cohorts predominantly consisting of patients with coronary artery disease (approximately 60%), whereas only about 30% had a history of cerebrovascular disease. In these original derivation and external validation cohorts, overall C-statistics ranged from 0.60 to 0.68.^4^^,^^9^^,^^13^ SMART-REACH provides a longer-term risk assessment compared with earlier late risk models for stroke patients, such as the Essen risk score, which predicts 1-year recurrent MACE with reported C-statistics ranging from 0.56 to 0.63.^14–17^ However, in the original development cohorts, limited information was available on demographics, stroke aetiology and stroke severity among patients with prior cerebrovascular disease, restricting insights into performance of SMART-REACH in stroke-only populations to date.

Only one earlier study has validated SMART-REACH in an ischaemic stroke population. This included 465 patients followed for 2 years, and reported modest discrimination (C-statistic 0.63; 95% CI, 0.55–0.71).^18^ Our study, conducted in a much larger contemporary sample, suggests limited discrimination of the SMART-REACH model for risk prediction after stroke (C-statistic 0.59; 95% CI, 0.56–0.63). We also identified limited calibration of the model in CONVINCE, and an underestimation of the actual recurrence risk. The findings may have clinical implications. While the SMART-REACH risk score is publicly available and could in principle be incorporated into clinical practice, its modest discriminative power and tendency to underestimate absolute risk necessitate caution when using it for individualised risk assessment following ischaemic stroke, until further validation or stroke-specific recalibration is conducted. Nevertheless, the model can still serve as a useful tool; in our cohort, patients classified as very high risk (≥30%) experienced substantially higher absolute event rates, indicating that the score remains valuable for population-level risk stratification and for identifying high-risk subgroups in clinical trials.

The role of colchicine in secondary stroke prevention remains unconfirmed. The CONVINCE trial was under-powered because of slow recruitment during the COVID-19 pandemic which reduced the available follow-up time and did not demonstrate a statistically significant benefit for the primary efficacy endpoint (HR 0.84; 95% CI, 0.68–1.05), although the colchicine-treated group had a numerically lower event rate (9.8% vs 11.7%).^8^ The intention-to-treat analysis reflects the effect of treatment assignment regardless of treatment adherence, whereas the on-treatment analysis captures the effect among colchicine-compliant patients. In the latter analysis, a treatment effect was observed in patients treated with colchicine (HR 0.80; 95% CI, 0.63–0.99). Additional subgroup analyses suggested benefit in patients with prior atherosclerotic coronary artery disease (HR 0.57; 95% CI, 0.35–0.94), but not in those without (HR 0.95; 95% CI, 0.75–1.21).^8^ A meta-analysis on the long-term use of colchicine in patients with a history of stroke or coronary disease demonstrated a 27% relative risk reduction for both ischaemic stroke and MACE.^19^

For colchicine-adherent patients with ≥ 30% 10-year ASCVD risk, we observed an ARR of 5.5% for MACE (number needed to treat [NNT] 18) and 4.2% for ischaemic stroke (NNT 24). Our results generate the hypothesis that patients with evidence of atherosclerotic disease and high baseline predicted risk may have greater benefit in future trials of colchicine for secondary stroke prevention. Specifically, in patients with ≥ 20% 10-year ASCVD risk, risk reductions might be more pronounced compared to unselected patients. Patient selection according to baseline CRP level might further improve risk stratification, given its association with recurrent cardiovascular events in patients with ischaemic stroke or TIA.^20^

Our study is the first to assess the benefit of colchicine according to baseline ASCVD risk in a stroke population. The effect of colchicine in patients with chronic coronary artery disease has been previously studied, indicating that estimated 10-year ARRs from low-dose colchicine were largest for patients with high baseline risk of CVD, as defined by the SMART-REACH model.^9^ While those analyses relied on estimated benefits, our results are derived from real-life data observed in a randomised trial. However, we did not evaluate lifetime benefits, which are typically more pronounced in younger patients, irrespective of baseline risk.^21^ Therefore, we cannot rule out the possibility of long-term benefit in (younger) patients with a lower ASCVD risk profile, who may derive more sustained treatment effects over time. However, given the lack of a significant interaction, any observed differences between ASCVD risk subgroups should be considered exploratory rather than evidence of differential efficacy.

Strengths of our study include the use of high-quality randomised controlled trial data from a large cohort of stroke patients with diverse ASCVD risk factors. We validated the SMART-REACH risk score in a stroke population with long-term follow-up. All relevant predictors required for calculating the 10-year ASCVD risk using SMART-REACH were available in CONVINCE, with only very limited missing data. The SMART-REACH model was developed for individuals aged 45–80 years, whereas 9.1% of our cohort fell outside this range. Threshold imputation (45/80 years) may have resulted in slightly more conservative risk estimates in older patients; however, this approach ensured model validity. We acknowledge this was an exploratory analysis, as patients were not stratified by ASCVD risk at the time of randomisation in CONVINCE. However, we did not observe important differences in baseline characteristics beyond those expected based on the SMART-REACH model. A further limitation is the inability to dynamically assess changes in ASCVD risk factors during follow-up. While ASCVD risk factors may evolve over time (eg, smoking cessation, adequate treatment of hypertension, etc), such changes were not accounted for in our analysis. In addition, this trial population received intensive contemporary secondary prevention therapy. Together, these factors could theoretically lead to an overestimation of ASCVD risk. However, this was not observed in our study. On the contrary, the SMART-REACH model appeared to systematically underestimate ASCVD risk in this population compared to the observed event rate. A further limitation is the higher proportion of patients categorised as high to very high risk, compared with those at low to moderate risk.

## Conclusion

In conclusion, although we observed a higher frequency of MACE outcomes following stroke in patients with higher baseline SMART-REACH score, the SMART-REACH model had poor discrimination and limited calibration in the CONVINCE population. Further studies are needed to validate SMART-REACH and to improve long-term risk prediction models after stroke. Although no interaction between treatment and baseline ASCVD risk group was observed, we documented a trend toward greater benefit in colchicine-treated patients with higher ASCVD risk. Our findings suggest that future studies investigating anti-inflammatory treatment in secondary prevention after TIA or stroke could consider prioritising patients with underlying atherosclerotic disease.

## Group authorship

CONVINCE investigators.

**Table:** 

Name	Location	Role	Contribution
G Vanhooren	AZ Sint-Jan, Belgium	Site investigator	Data acquisition
D Hemelsoet	UZ Gent, Belgium	Site investigator	Data acquisition
A Peeters	UCL St. Luc, Belgium	Site investigator	Data acquisition
L Sinnaeve	AZ St Lucas, Belgium	Site investigator	Data acquisition
M Hill	Foothills Medical Centre, Calgary, Canada	Site investigator	Data acquisition
M Haršány	St Anne’s University Hospital Brno, Czech Republic	Site investigator	Data acquisition
O Škoda	Hospital Jihlava, Czech Republic	Site investigator	Data acquisition
H Klingenberg Iversen	Rigshospitalet Glostrup, Denmark	Site investigator	Data acquisition
C Kruuse	Herlev Hospital, Denmark	Site investigator	Data acquisition
N Solokha	Nordsjællands Hospital Hillerød, Denmark	Site investigator	Data acquisition
J Korv	Tartu University Hospital, Estonia	Site investigator	Data acquisition
P Kraft	Klinikum Main-Spessart, Krankenhaus Lohr, Germany	Site investigator	Data acquisition
T Neumann-Haefelin	Klinikum Fulda, Germany	Site investigator	Data acquisition
H Soda	Rhön-Klinikum Campus Bad Neustadt, Germany	Site investigator	Data acquisition
K G Häusler	Universitätsklinikum Würzburg, Germany	Site investigator	Data acquisition
B Schmitz	Vivantes Humboldt-Klinikum, Germany	Site investigator	Data acquisition
G Thomalla	Universitätsklinikum Hamburg-Eppendorf, Germany	Site investigator	Data acquisition
C Krogias	Katholisches Klinikum Bochum gGmbH, Germany	Site investigator	Data acquisition
S Murphy	Mater Misericordiae University Hospital, Ireland	Site investigator	Data acquisition
S Cronin	Cork University Hospital, Ireland	Site investigator	Data acquisition
R Collins	Tallaght University Hospital, Ireland	Site investigator	Data acquisition
D McCabe	Tallaght University Hospital, Ireland	Site investigator	Data acquisition
D Williams	Beaumont Hospital, Ireland	Site investigator	Data acquisition
J Harbison	St James’s Hospital, Ireland	Site investigator	Data acquisition
E Dolan	Connolly Hospital, Ireland	Site investigator	Data acquisition
P Cotter	St Luke’s General Hospital, Ireland	Site investigator	Data acquisition
P Hickey	Sligo University Hospital, Ireland	Site investigator	Data acquisition
P Bienfait	Gelre Ziekenhuis Apeldoorn, Netherlands	Site investigator	Data acquisition
A Członkowska	Institute of Psychiatry and Neurology, Poland	Site investigator	Data acquisition
W Brola	Specialist Hospital of Saint Luke, Poland	Site investigator	Data acquisition
M Baptista	Hospital Egas Moniz, Portugal	Site investigator	Data acquisition
J Marto	Hospital Egas Moniz, Portugal	Site investigator	Data acquisition
E Cuadrado	Hospital del Mar, Spain	Site investigator	Data acquisition
M Castellanos	CHU A Coruña, Spain	Site investigator	Data acquisition
D Cánovas	Parc Taulí Sabadell, Spain	Site investigator	Data acquisition
M Medina	Hospital Virgen del Rocío, Spain	Site investigator	Data acquisition
J Masjuan	Hospital Ramón y Cajal, Madrid, Spain	Site investigator	Data acquisition
M Gomez-Choco	Hospital Moisès Broggi, Spain	Site investigator	Data acquisition
M J Serena	Hospital Josep Trueta Girona, Spain	Site investigator	Data acquisition
S Perez Sanchez	Hospital Virgen Macarena, Spain	Site investigator	Data acquisition
J Fernandez Dominguez	Centro Médico de Asturias, Spain	Site investigator	Data acquisition
M Heldner	University Hospital Bern, Switzerland	Site investigator	Data acquisition
M Barber	Monklands Hospital, United Kingdom	Site investigator	Data acquisition
N Evans	Addenbrooke’s Hospital, United Kingdom	Site investigator	Data acquisition
G Shim	North Durham University Hospital, United Kingdom	Site investigator	Data acquisition
L Sztriha	King’s College Hospital, United Kingdom	Site investigator	Data acquisition
K Adie	Royal Cornwall Hospital, United Kingdom	Site investigator	Data acquisition
A Ali	Royal Hallamshire Hospital, United Kingdom	Site investigator	Data acquisition
M Garside	Northumbria Hospital, United Kingdom	Site investigator	Data acquisition
L D’Anna	Charing Cross Hospital, United Kingdom	Site investigator	Data acquisition
L Sekaran	Luton & Dunstable University Hospital, United Kingdom	Site investigator	Data acquisition
A Bhalla	St Thomas’ Hospital, United Kingdom	Site investigator	Data acquisition
B Keegan	South West Acute Hospital, United Kingdom	Site investigator	Data acquisition
K Rashed	Yeovil District Hospital, United Kingdom	Site investigator	Data acquisition
T England	Royal Derby Hospital, United Kingdom	Site investigator	Data acquisition
G Gunathilagan	Queen Elizabeth The Queen Mother Hospital, United Kingdom	Site investigator	Data acquisition
S Ivatts	St Richard’s Hospital, United Kingdom	Site investigator	Data acquisition
P Guyler	Southend University Hospital, United Kingdom	Site investigator	Data acquisition
D Lashley	Derriford Hospital, United Kingdom	Site investigator	Data acquisition
A Elmerimi	Lincoln County Hospital, United Kingdom	Site investigator	Data acquisition
M Hasan	Peterborough City Hospital, United Kingdom	Site investigator	Data acquisition
T Hlaing	Aintree University Hospital, United Kingdom	Site investigator	Data acquisition
M Burn	Wycombe Hospital, United Kingdom	Site investigator	Data acquisition

## Supplementary Material

Supplemental_Materials_aakag033

## Data Availability

The data that support the findings of this study are available from the corresponding author upon reasonable request. The data are not publicly available due to privacy or ethical restrictions.
