# Supplementary material for: Validation of the SMART-REACH model after stroke and the effect of colchicine by atherosclerotic cardiovascular disease risk category: a secondary analysis of the CONVINCE randomised clinical trial
Source: Eur Stroke J. 2026 Apr 25;11(4):aakag033. doi: 10.1093/esj/aakag033 (PMC13109098; doi:10.1093/esj/aakag033)
Supplement: Supplemental_Materials_aakag033 [file supplemental_materials_aakag033.zip › Supplemental_Materials_aakag033_NEW/Supplemental Material - Statistical Analysis Plan final.pdf]

STATISTICAL ANALYSIS PLAN

CONVINCE

*CO*lchicine for preventio*N* of *V*ascular *I*nflammation in *N*on-*C*ardio*E*mbolic stroke – a randomised clinical trial of low-dose colchicine for secondary prevention after stroke

Version: 2.0, 18 October 2023

CONTENTS:

Section		Page
1.	Administrative information	3
2	Introduction	6
3	Study methods	7
4	Statistical principles	9
5	Trial sample	11
6	Analysis	13
7	Procedure for amendments to the SAP	16
8	References	17

Section 1: Administrative information:

Title:

*CO*lchicine for preventio*N* of *V*ascular *I*nflammation in *N*on- *C*ardio*E*mbolic stroke – a randomised clinical trial of low-dose colchicine for secondary prevention after stroke **CONVINCE**

Trial registration number:

EudraCT number: 2015-004505-16

SAP Version number:

SAP version 2.0, 18th October 2023

Refers to:

Trial protocol identification: CON-001

Protocol date and version number: 12 August 2020, Version 3.0

Version information:

This update of the CONVINCE Statistical Analysis Plan (SAP, Version 2, 18.10.23) was written to ensure clear and transparent compliance with guidance for best practice for Statistical Analysis Plans for randomised controlled trials (Refers to: Gamble C et al, Guidelines for content of Statistical Analysis Plans in Clinical Trials (JAMA 2017;318(23);2337-2343)). It was written in advance of the data lock and analysis. All members of the Steering Committee and SAP writing/review group are blinded to the results of the trial at the time of writing.

Version history:

**Document**	**Date of Issue, Version no**	**Summary of Change**
SAP Version 1.0	7th March 2016,	Not applicable, original
SAP Version 2.0	18th October 2023	Alignment of SAP to recommended guidelines for content and reporting of Statistical Analysis Plans in Clinical Trials[1] (JAMA 2017;318(23);2337-2343).<br><br>Changes made after 2 pre-specified interim analyses conducted by the Data Monitoring Committee. SAP writers, Trial Steering Committee and Trial Statistician remain blinded to results of the interim analyses at the time of completion of SAP V2.

Roles and responsibilities:

Sponsor: University College Dublin, Belfield, Dublin 4, Ireland

Chief Investigator and SAP Co-author: Prof. Peter Kelly, Mater Misericordiae University Hospital, Dublin 7, and Stroke Clinical Trials Network Ireland, University College Dublin, Ireland

Trial Statistician and SAP Co-author: Prof Cathal Walsh, Professor of Biostatistics, Trinity College Dublin Ireland

SAP revision review group:

Prof Michael Hill, University of Calgary, Canada

Prof Robin Lemmens, University of Leuven, Belgium

Prof Christopher Price, University of Newcastle, UK

Prof Peter Sandercock, University of Edinburgh, UK

Document primacy:

This SAP is the final and complete description of the pre-specified analysis plan for CONVINCE. In case of ambiguity or discrepancy with other trial documents, the SAP has primacy.

Reporting of the SAP:

Prior to the data lock and statistical analysis of outcomes assigned by blinded adjudicators, the SAP will be made publicly available, either through journal publication following peer-review, posting on the trial website, or both[1,2].

Signature Page:

I confirm that all information in this Statistical Analysis Plan is accurate to the best of my knowledge.

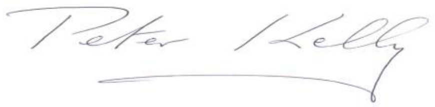

____

Peter Kelly, MD MS FRCPI FESO

Professor of Neurology (Adjunct)

University College Dublin

Chief Investigator, CONVINCE

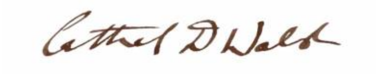

____

Cathal Walsh

Professor of Biostatistics

Trinity College Dublin

Trial Statistician, CONVINCE

Section 2: Introduction:

Background and rationale:

Inflammation is a key contributor to first and recurrent stroke, myocardial infarction and vascular death. It is unknown if anti-inflammatory agents reduce the risk of recurrent stroke and other vascular events in survivors of first stroke or transient ischaemic attack. Colchicine is an inexpensive, orally-administered anti-inflammatory agent with an established safety and tolerability profile when used in low dose over prolonged periods. CONVINCE is a randomised trial to evaluate the efficacy and safety of low dose daily colchicine in addition to usual care, compared with usual care alone, for the prevention of secondary vascular events after stroke or transient ischaemic attack[3].

Objectives:

Primary objective:

We hypothesise that low-dose colchicine will prevent recurrent ischaemic stroke and coronary events in survivors of first stroke and transient ischaemic attack. Our primary objective is to investigate the efficacy of low dose colchicine (0.5mg/day) plus usual care as indicated (defined as antiplatelet, lipid-lowering, antihypertensive treatment, and appropriate lifestyle advice) compared with usual care alone to prevent non-fatal recurrent ischaemic stroke, myocardial infarction, cardiac arrest, hospitalization for unstable angina and vascular death after ischaemic stroke or transient ischaemic attack (TIA) not caused by cardiac embolism or other defined causes unrelated to atherosclerosis

Secondary objectives:

We hypothesise that low dose colchicine will be safe (ie. no excess of serious adverse events related to the study drug will be observed) and that the effect of treatment will be consistent for components of the composite outcome and across important subgroups of patients. Our secondary objectives are:

1. To investigate the safety of low dose colchicine (0.5mg/day) plus usual care compared with usual care alone.
2. To investigate the effect of colchicine on each component of the composite primary outcome.
3. To investigate the effect of colchicine on fatal and non-fatal ischaemic stroke combined.
4. To investigate the effect of colchicine on recurrent disabling and non-disabling ischaemic stroke.
5. To assess whether the effect of treatment on the primary outcome is materially different among different categories of patient defined at baseline or during the trial, including but not limited to demographic and clinical characteristics, adherence, baseline and on-treatment high-sensitivity C-reactive protein (hsCRP)
6. To investigate the effect of colchicine on direct health care costs

Section 3: Study methods:

Trial design:

CONVINCE is a randomised, open-label, blinded endpoint-assessed, parallel group Phase 3 clinical trial, comparing low-dose colchicine plus usual care to usual care alone for prevention of recurrent non-fatal ischaemic stroke, myocardial infarction and cardiac arrest, hospitalization for unstable angina and vascular death after ischaemic stroke or TIA, not caused by cardiac embolism or other defined mechanisms unrelated to atherosclerosis. After an initial Vanguard Stage which recruited less than 10% of the total sample to establish trial procedures and verify recruitment projections, the trial continued seamlessly to the Full Trial stage. No efficacy analysis was done after the end of the Vanguard Stage and all patients included in the Vanguard are included in the Full Trial.

Randomisation:

Randomisation will take place via an Interactive Web Response System (IWRS) supplied by Sealed Envelope, UK. Randomisation will be conducted using a minimisation algorithm, to ensure groups are balanced for key prognostic variables affecting recurrent stroke risk, including the following mandatory variables:

1. age (less than 70, 70 or greater)
2. time since qualifying stroke/TIA (7 days or less, greater than 7 days)
3. type of qualifying event (stroke or TIA).

In addition, if information on large artery stenosis is available at the time of randomisation, the algorithm will include these data, although it is not a mandatory item.

Sample size:

The original sample size for CONVINCE was 2,623 patients when designed in 2014. Based on contemporary data suggesting reduced annual rates of recurrent stroke and cardiac events, as a precautionary measure to maximise statistical power, a protocol amendment was made to increase the trial sample size. In brief, this was calculated as follows: Assuming an annualized rate of the composite outcome in controls of 4.5%/year (13.5% over median 3-year follow-up) and a relative hazard of 0.75in colchicine-treated patients after adjusting for a 15% non-compliance rate (alpha 0.05, power 80%), the revised sample size is calculated at 3,154 patients (Refer to Protocol, Appendix 13, for detailed justification and revised sample size calculation).

Framework:

The primary analysis is based on superiority of colchicine plus usual care, compared to usual care alone. Statistical analyses of secondary outcomes will also be based on superiority analyses.

Statistical interim analysis and stopping guidance:

Interim analyses and stopping guidance are also outlined in the CONVINCE DMC Charter.

Number of Interim Analyses:
2 interim analyses will be conducted prior to the final analysis, when approximately 50% and 75% of subjects have been randomised. The interim analyses are conducted by the Independent Statistician member of the Data Monitoring Committee (DMC), unblinded to accumulating outcome data according to study arm, and are shared with the DMC members in closed session[4]. The Chief Investigator, Trial Statistician, Trial Steering Committee (TSC), Project Team, and Investigators will remain strictly blinded to the results.

Early Stopping Rules:
For both interim analyses, using the Haybittle-Peto principle, a statistical threshold of ≤0.001 will apply for consideration for early stopping for efficacy. This threshold will refer to comparison of the primary composite outcome measure, and not individual components of the outcome, and will apply to a between-group comparison of time to primary outcome using the log-rank test. The DMC, at their discretion, may also examine the between-group effect size analysed by Cox proportional hazards modelling, adjusting for minimisation variables, expressed as the hazard ratio and confidence intervals.
No statistical rules are applied for early stopping for futility or safety. A recommendation for early stopping for safety reasons may be considered by the DMC based on the accumulating safety data provided in tabular format, clinical experience, and relevant data from other trials and systematic reviews.

Related considerations:
When deliberating, the DMC will consider the totality of evidence, including the results of statistical interim analyses, precision and magnitude of treatment effect, potential for knowledge gain or loss by stopping early, and clinical implications in practice such as acceptance/uptake. The DMC may wish to observe consistent direction of benefit across components of the composite outcome before recommending early stopping for efficacy.

Unanimity is preferred among DMC members in a decision to advise early termination for benefit to the TSC. If unanimity is not possible, a clear majority decision is recommended. DMC recommendations on stopping or continuing are advisory to the TSC, who will make the ultimate decision after careful consideration.

Timing of final analysis:

Final analysis will be done when all outcomes are collated and verified, patient end-of-trial vital status verified (living/dead/unknown, outcome/no outcome/unknown) and database is locked.

Timing of outcome assessments:

Outcomes are assessed at follow up visits conducted at post-randomisation day 28, 90, and at 6-monthly intervals to a maximum of 84 months.

Section 4: Statistical principles:

<u>Confidence intervals and P values:</u>

The level of statistical significance:

For the primary analysis, a P-value of 0.048 or less will be considered as statistically-significant (two-sided alpha), accounting for the 'alpha-spend' of 0.001 in each of 2 interim analysis.

Multiple comparisons:
For secondary analyses, a pre-specified hierarchical analysis plan will be implemented[5,6]. A P-value <0.05 or less will be considered statistically-significant. Statistical significance will be required on each analysis for interpretation of the subsequent analysis as confirmatory. If statistical significance is not reached on the primary analysis or a secondary analysis, subsequent secondary analyses will be interpreted as hypothesis-generating. No statistical adjustment for multiplicity will be made for secondary analyses.

Confidence intervals:

Uncertainty around effect estimates will be reported as 95% confidence intervals, including the effect size (expressed as hazard ratio) and estimate precision for the primary analysis.

<u>Adherence and protocol deviations:</u>

Adherence:

Permanent colchicine discontinuation will be defined when a patient originally randomised to colchicine is recorded as permanently discontinued colchicine at the visit preceding the end-of-trial visit, or last trial visit before a primary outcome event, non-cardiovascular death, or loss-to-follow-up. Adherence expressed as numbers and percentages of patients with permanent discontinuation will be reported[7].

Numbers and percentages of patients with never-initiated status (randomised to colchicine, no dose received), and early discontinuation status (randomised to colchicine, permanently discontinued at 28 day visit) will also be reported. The median duration of adherence to colchicine treatment will be reported, with inter-quartile ranges.

Protocol deviations:

Protocol deviations will be classified as major if they affect the scientific integrity of the trial, are major errors in inclusion criteria, or are likely to have a significant impact on patient safety. Examples include recruitment of patients originally diagnosed as stroke/TIA at onset for whom the diagnosis is subsequently revised, permanent non-adherence among patients

randomised to receive colchicine, or use of colchicine in patients randomised to usual care (cross-overs). Only major protocol deviations will be reported. Major deviations expressed as numbers and percentages of patients will be reported stratified by randomised arm.

Analysis populations:

The ITT population is defined as all consenting patients randomised to colchicine or usual care (ie. assigned to intervention or control arms). The flow of study participants will be described in a CONSORT diagram.[8.] Analysis of the primary outcome measure will be performed on the intention-to-treat (ITT) population.

The per-protocol population is defined as those study subjects without major protocol deviations related to pre-randomisation eligibility criteria, who remained adherent to the assigned study arm (colchicine or usual care) post-randomisation, and had no other post-randomisation major protocol deviations. (Non-adherence for this analysis will be defined as permanent discontinuation for reasons other than appropriate clinical reasons)[9,10]. Analysis of the primary outcome measure will be repeated in the per-protocol population, with statistical adjustment for confounding due to incomplete adherence. The findings will be interpreted as confirmatory of the primary ITT analysis.

The 'safety population' is defined as all randomised patients providing consent who initiated at least a single dose of colchicine (if randomised to the intervention arm).

Section 5: Trial sample:

Screening data:
Screening logs will not be kept.

Eligibility:

Detailed eligibility criteria are listed in the Protocol and briefly summarised below:

Inclusion criteria are:

1. Written informed consent provided
2. Age 40 years or greater
3. Patient has had either an ischaemic stroke without major disability (modified Rankin score 3 or less) or high-risk TIA (motor/speech with ABCD2 score>/=4, or DWI positive, or large artery stenosis >/=50%), and brain imaging excluding haemorrhage, and randomisation 72 hours-28 days post-onset of qualifying event
4. Qualifying stroke/TIA probably caused by large artery stenosis, small artery occlusion (lacunar stroke), or cryptogenic embolism
5. eGFR >/= 50 ml/min
6. Patient medically- stable, capable of participating in a randomised trial

Main exclusion criteria:
1. Stroke/TIA due to cardiac embolism or other defined causes
2. Medical conditions raising safety concerns by colchicine therapy
3. Taking concomitant medications contra-indicated with colchicine therapy
4. Medical conditions precluding participation in a randomised trial
5. Women of child-bearing potential

Recruitment:
The CONSORT flow diagram[8] in the main and subsequent publications from the trial will describe:
1. numbers of patients randomised to each arm
2. numbers who did and did not receive the allocated study intervention
3. numbers lost to follow up
4. numbers included in the ITT analysis

Non-outcome deaths and withdrawal/loss to follow-up:
Numbers of deaths due to causes other than outcomes and numbers lost to follow up will be provided in each randomised arm.

Baseline characteristics:
The following baseline characteristics will be presented in a table or tables as numbers and proportions of the totals, or mean/median with standard deviations/interquartile ranges, in each randomised arm: age, sex, ethnicity, qualifying event, onset-randomisation interval, ABCD2 score, NIH stroke score, baseline imaging type, large artery stenosis, current smoking, hypertension, diabetes, prior stroke, prior coronary disease, LDL and total

cholesterol, hsCRP, eGFR, antiplatelet monotherapy, antiplatelet dual therapy, other antithrombotic therapy, statin therapy, antihypertensive therapy.

Section 6: Analysis:

Outcome definitions:

The primary outcome is defined as the first event in the composite of non-fatal recurrent ischaemic stroke, non-fatal myocardial infarction, non-fatal cardiac arrest, hospitalization for unstable angina or vascular death.

Secondary outcomes are defined as:

(1) Key secondary outcome: Composite of first non-fatal recurrent ischaemic stroke, non-fatal myocardial infarction, non-fatal cardiac arrest, or vascular death

Individual components of the primary composite outcome, defined as:

(2) Fatal and non-fatal recurrent ischaemic stroke combined
(3) Non-fatal ischaemic stroke,
(4) Vascular death
(5) non-fatal MI and cardiac arrest
(6) unstable angina requiring hospitalisation

Exploratory outcomes and analyses:

All exploratory analyses will be interpreted as hypothesis-generating. These analyses will examine the effect of colchicine therapy on:

(1) recurrent disabling ischaemic stroke (modified Rankin score [MRS] 3-5)
(2) Disability (modified Rankin score) of recurrent strokes measured across the entire range of the modified Rankin score
(3) Recurrent severe ischaemic stroke (fatal or MRS 4-5)
(4) direct healthcare resource costs
(5) cognitive decline and dementia, measured by the Montreal Cognitive Assessment (MOCA)
(6) health-related quality of life, measured by EuroQoL (EQ5D-5L)
(7) cumulative total number of component events in the primary outcome cluster detected over the duration of the trial
(8) primary and secondary outcome events stratified by baseline and on-treatment high-sensitivity C-reactive protein (hsCRP)

Safety:

The following safety outcomes will be described by reporting numbers and proportions in each treatment arm:

1. Fatal events not coded as outcomes

2. All serious adverse events, defined as per the Protocol

3. Selected pre-specified non-serious adverse events (defined in the Protocol), specifically:

   a. Gastrointestinal (vomiting, nausea, diarrhoea)

b. Myalgia requiring discontinuation of study medication or myopathy
c. Hepatic impairment (transaminases (AST or ALT) ≥2 ULN)
d. Myelosuppression
e. Moderate or severe renal impairment
f. Peripheral neuropathy
g. Rash, itch, or alopecia
h. Major haemorrhage

Analysis methods:

The statistical approach will be a comparison of time to primary outcome event (first recurrent event in the outcome composite) in colchicine-treated and usual-care groups. The effect size and precision will be reported as hazard ratio with 95% confidence intervals using Cox proportional hazards modelling. The hazard ratio will be adjusted for the three mandatory minimisation variables (age, duration since last event, type of qualifying event) specified in the randomisation algorithm.

The proportional hazards assumptions will be verified by inspection of log-log plots and examination of the Schoenfeld residuals. Deviations from the proportionality assumption will be resolved by stratification and the incorporation of time covariate interactions in the model as required.

For secondary analyses of recurrent events, these will be analysed as time-to-event analyses using Cox proportional hazard modelling.

Subgroup analyses will be done to examine the consistency of therapeutic effect across important clinical subgroups: age, sex, qualifying event (stroke or TIA), time to randomisation (≤7days or >7 days), hypertension, diabetes, current smoking, prior stroke, prior coronary disease, statin therapy, baseline LDL, subsequent atrial fibrillation detected

<u>Procedure for accounting for missing, unused and spurious data</u>

For the primary analysis, missing outcome data will be considered as censored at the date last known to be alive. Sensitivity analyses will be undertaken to evaluate the effect of the missingness in the data. Firstly, in the worst-case scenario, all lost to follow-up patients will be considered to have had an event at the time last known to be alive. Secondly the worst-comparison scenario will be examined with losses in the treatment group taken as having had an event while losses in the control group being censored. This imputation method is not biased towards a positive treatment effect.

For secondary analyses of continuous outcome variables which have been measured on multiple occasions the primary analysis will be by a Mixed Model Repeated Measures approach. This technique imputes the final value using the actual values measured when

the final outcome is missing. For a sensitivity analysis a multiple imputation approach to missing values will be taken. Five imputations will be performed sampling from a posterior Bayesian distribution of the baseline values of the variable in question. Sampling from the baseline distribution values does not assign any treatment benefit in the imputed values.

Multiple imputation will also be used for key non-outcome variables. Using a regression approach the non-missing confounders in the model will be used to impute the missing confounder with a multivariate random component added in. Five imputations will be carried out. A sensitivity analysis will be undertaken looking at the effects of other and no imputation methods on the trial outcome. Data will not be imputed where more than 15% of cases are missing for a given variable.

Section 7: Procedure for reporting any amendment to this SAP:

Procedures for amending this version of the SAP will be:

1. Review of justification and proposed revised approach to the original statistical plan by the Trial Statistician and Steering Committee
2. Approval or non-approval of revised approach, by Steering Committee
3. Incorporation of revised approach into SAP as an amendment, with clear justification and date of revision, and procedure for publication and/or posting on the trial website
4. Any analysis plan amendment before the date of data lock will be classified as Pre-specified. Any amendment occurring during the analysis will be classified as post-hoc.

Section 8: References:

1. Gamble C et al, Guidelines for content of Statistical Analysis Plans in Clinical Trials (JAMA 2017;318(23);2337-2343
2. Hemming K, Kearney A, Gamble C, et al. Prospective reporting of statistical analysis plans for randomised controlled trials. Trials 2020;21:898
3. Kelly PJ, Weimar C, Lemmens R, et al, Colchicine for prevention of vascular inflammation after non-cardioembolic stroke – study protocol for a randomised controlled trial, Eur Stroke J 2021;222-228
4. Ellenberg SS, Fleming TR, DeMets DL. Data Monitoring Committees in Clinical Trials-A Practical Perspective. Statistics in Practice, John Wiley and Sons, Chichester, England, 2002
5. European Agency for Evaluation of Medicinal Products, Committee for Proprietary Medicinal Products. Points to consider on multiplicity issues in clinical trials. 2002. https://www.ema.europa.eu/en/documents/scientific-guideline/points-consider-multiplicity-issues-clinical-trials_en.pdf. Accessed 17.10.2023
6. Parker RA, Weir CJ. Multiple secondary outcome analyses: precise interpretation is important. Trials 2022;23:27
7. Valgimigli M, Garcia-Garcia HM, Vrijens B, et al. Non-adherence Academic Research Consortium standardised classification and framework for reporting, interpreting, and analysing medication non-adherence in cardiovascular clinical trials. Eur Heart J, 2019;40:2070-2085
8. Schulz KF, Altman DG, Moher D, et al for the CONSORT group. CONSORT 2020 statement: updated guidelines for reporting parallel group randomised trials. Trials 2010;11:32: PMID:20334632
9. Hernan MA, Robins JM. Per-Protocol analysis of pragmatic trials. N Engl J Med 2017;377:1391-98
10. Smith VA, Coffman CJ, Hudgens MG. Interpreting the results of intention-to-treat, per-protocol, and as-treated analyses of clinical trials. JAMA 2021;326:433-34
