# Supplementary material for: Validation of the SMART-REACH model after stroke and the effect of colchicine by atherosclerotic cardiovascular disease risk category: a secondary analysis of the CONVINCE randomised clinical trial
Source: Eur Stroke J. 2026 Apr 25;11(4):aakag033. doi: 10.1093/esj/aakag033 (PMC13109098; doi:10.1093/esj/aakag033)
Supplement: Supplemental_Materials_aakag033 [file supplemental_materials_aakag033.zip › Supplemental_Materials_aakag033_NEW/Supplemental Material - Study Protocol Final.pdf]

CLINICAL TRIAL PROTOCOL

CONVINCE

*CO*lchicine for preventio*N* of *V*ascular *I*nflammation in *N*on-*C*ardio*E*mbolic stroke – a randomised clinical trial of low-dose colchicine for secondary prevention after stroke

Protocol, Version: 3.0 12 August 2020

Sponsor: University College Dublin, Belfield, Dublin 4, Ireland

Chief Investigator: Prof. Peter Kelly, Mater Misericordiae University Hospital, Nelson Street, Dublin 7

This document contains confidential information that must not be disclosed to anyone other than the sponsor, the investigative team, regulatory authorities, and members of the Research Ethics Committee.

1 STUDY DETAILS

1.1 Study title

CONVINCE (COlchicine for preventioN of Vascular Inflammation in Non-CardioEmbolic stroke) – a randomised clinical trial of low-dose colchicine for secondary prevention after stroke

1.2 Reference numbers

Protocol identification: CON-001
EudraCT number: 2015-004505-16
Date and version number: 12 August 2020, Version 3.0

1.3 Investigator details

Chief investigator & Trial Medical Expert

Prof Peter Kelly,

UCD Clinical Research Centre, Mater Misericordiae University Hospital, Nelson Street, Dublin 7

Tel: +353 (0) 18301122

Email: pjkelly@mater.ie

Sponsor
Name: University College Dublin, Belfield, Dublin 4, Ireland
Contact details: Prof Peter Doran, peter.doran@ucd.ie

Trial Coordinating Centre
Irish Stroke Clinical Trials Network, Project Office, UCD Clinical Research Centre, Mater Misericordiae University Hospital, 27 Nelson Street, Dublin 7 (+3531 7164576, isctn@ucd.ie)

Funder:
Health Research Board of Ireland, Grattan House, 67-72 Lwr Mount St, Dublin 2
Contact details: Oonagh Ward, oward@hrb.ie

1.4 **Signatures**

A review of the protocol has been completed and is understood and approved by the following:

____

Prof Peter Kelly, Principal (Chief) Investigator

Date:

____

Prof Peter Doran, University College Dublin (Sponsor)

Date

1.5 Categorisation of study (Switzerland only, ClinO, Art. 19)

CONVINCE is a clinical trial of medicinal products under category C (colchicine is not authorised in Switzerland)

2 SITE INVESTIGATOR SIGNATURE

I, the undersigned, am responsible for the conduct of the trial at this site (named below) and agree to the following:

- I understand and will conduct the trial according to the protocol, any approved protocol amendments, ICH GCP and all applicable regulatory authority requirements and national laws.
- I will not deviate from the protocol without prior written permission from the Sponsor and prior review and written approval from Independent Ethics Committee, except where necessary to prevent any immediate danger to the subject.
- I have read and understand fully the Summary of Product Characteristics (SPC) for colchicine; and I am familiar with its use according to this protocol.
- I have sufficient time to properly conduct and complete the trial within the agreed trial period, and I have available an adequate number of qualified staff and adequate facilities for the foreseen duration of the trial to conduct the trial properly and safely.
- I will ensure that any staff at my site(s) who are involved in the trial conduct are adequately trained regarding the Investigational Medicinal Products, the protocol and their responsibilities. In the case of delegating any of my trial responsibilities I will provide the Sponsor with a Delegation of Activities certificate.

Site Lead Investigator Name (BLOCK CAPITALS): ______________________________

Site Lead Investigator Signature:____

Date: _________________________________

Site Name and Country (BLOCK CAPITALS): __________________________________

TABLE OF CONTENTS

1 STUDY DETAILS 2
2 SITE INVESTIGATOR SIGNATURE 4
3 DOCUMENT HISTORY 6
4 SYNOPSIS 9
5 ABBREVIATIONS 13
6 INTRODUCTION 15
7 STUDY OBJECTIVE 19
8 DEFINITION OF OUTCOME MEASURES 20
9 TRIAL DESIGN 22
10 OVERVIEW OF TRIAL MEDICATION 36
11 SAFETY AND QUALITY MONITORING AND REPORTING 39
12 STATISTICS 47
13 DIRECT ACCESS TO SOURCE DATA/DOCUMENTS 50
14 DATA HANDLING AND RECORD KEEPING 51
15 RETENTION OF ESSENTIAL DOCUMENTS 51
16 QUALITY CONTROL AND QUALITY ASSURANCE PROCEDURES 52
17 AUDITS AND INSPECTIONS 52
18 ETHICS 52
19 FINANCING AND INSURANCE/INDEMNITY 54
20 CLINICAL STUDY REPORT 54
21 REFERENCES 55
APPENDIX 1: MODIFIED RANKIN SCORE, STANDARDISED ALGORITHM 58
APPENDIX 2: ABCD2 SCORE 60
APPENDIX 3: MONTREAL COGNITIVE ASSESSMENT (MOCA) 61
APPENDIX 4: NIH STROKE SCALE 66
APPENDIX 5: EQ5D-5L 74
APPENDIX 6: UNIVERSAL DEFINITION OF MYOCARDIAL INFARCTION, TIMI DEFINITION FOR HOSPITALIZATION OF UNSTABLE ANGINA 76
APPENDIX 7: EXPECTED ADVERSE EVENTS AFTER STROKE 79
APPENDIX 8: EXPECTED ADVERSE EVENTS AFTER COLCHICINE 83
APPENDIX 9: MINIMUM SOURCE DOCUMENTS FOR OUTCOME ADJUDICATION 85
APPENDIX 10: CREATININE CLEARANCE BY MDRD FORMULA 88
APPENDIX 11: CREATININE CLEARANCE BY CKD-EPI FORMULA 89
APPENDIX 12: DETAIL OF ALL CHANGES APPLIED TO PROTOCOL VERSION 3.0 90
APPENDIX 13: RE-ESTIMATION OF SAMPLE SIZE FOR CONVINCE 121
APPENDIX 14: STANDARD DEFINITIONS FOR TARGETED ADVERSE EVENTS 131
APPENDIX 15: CONVINCE PROCEDURES IN RESPONSE TO COVID-19 132

3 DOCUMENT HISTORY

Document	Date of Issue, Version no	Summary of Change
Protocol Version 1	7th March 2016,	Not applicable, original
Protocol Version 1	4th May 2016 (cover page), 7th March 2016 (internal protocol pages)	Response to VHP request. Added: four new exclusion criteria and amended one criterion<br>Wallet card and GP letter added<br>Clarified categories of review of study progress<br>See response to VHP 4th May 2016
Protocol Version 2	29 August 2016	**Substantial:**<br>The primary outcome cluster has been updated to include *an additional outcome event* of *Hospitalization for Unstable Angina.*<br><br>**Non-substantial**<br>See appendix 12
Protocol Version 2.1	29 November 2016	**Non-substantial:**<br>Multiple. Refer to Appendix 12, items 12-34
Protocol Version 2.1 (Switzerland)	18 June 2019	Non-substantial<br>- See appendix 12

Protocol Version 2.2 (Switzerland)	23 July 2019	Non-substantial<br>- See appendix 12
Protocol Version 2.1 (Netherlands)	13 Nov 2019	Non-substantial See appendix 12
Protocol Version 2.2	30 January 2019	**Non-substantial:** Additions made re replacement of study subjects and duration of document retention in Canada only. Refer to Appendix 12, items 35-36
Protocol Version 2.3	20 February 2019	Response to The Office of Clinical Trials, Health Canada.<br>Clarified Key Exclusion Criteria for Female Participants and Reproductive Risks of Colchicine<br>**Non-substantial**<br>Refer to Appendix 12, Item 37
Protocol Version 2.4	03 April 2019	Response to INFARMED, NCA Portugal. Additional text added to key exclusion criteria number 6: Impaired hepatic function. New text added:<br>"Bilirubin greater than twice upper limit of normal (ULN). Gamma-GT or alkaline phosphatase greater than 3 times ULN."

Protocol Version 2.5	11 February 2020	Protocol amended per Belgian Ethics Committee request:<br><br>Confirmation that data will be pseudo-anonymised, the only reference to the patient will be their CONVINCE Subject ID number.<br><br>Clarification that in Belgium all records and documents will be retained for 25 years after the end of the study. Compliance with Belgian regulations.
Protocol Version 3	12 August 2020	**Substantial**<br>Change to sample size<br><br>**Non-substantial**<br>See appendix 12

4 SYNOPSIS

Title of study	CONVINCE (COlchicine for preventioN of Vascular Inflammation in Non-CardioEmbolic stroke) – a randomised clinical trial of low- dose colchicine for secondary prevention after stroke
Name of sponsor/company	University College Dublin
Phase of development	Phase 3
Design	Prospective, Randomised Open-label, Blinded Endpoint assessment (PROBE) controlled clinical trial. This is a Phase 3 trial to compare low-dose colchicine (0.5mg/day) plus usual care, to usual care alone, to prevent recurrent ischaemic stroke and coronary events after non-cardioembolic stroke and TIA.
Objectives	Primary objective:<br><br>To investigate the efficacy of low dose colchicine (0.5mg/day) plus usual care (defined as antiplatelet, lipid-lowering, antihypertensive treatment, and appropriate lifestyle advice) compared with usual care alone to prevent non-fatal recurrent ischaemic stroke, myocardial infarction, cardiac arrest, hospitalization for unstable angina and vascular death after ischaemic stroke or transient ischaemic attack (TIA) not caused by cardiac embolism or other defined causes unrelated to atherosclerosis<br><br>Secondary objectives:<br>1. To investigate the safety of low dose colchicine (0.5mg/day) plus usual care compared with usual care alone.<br>2. To investigate the effect of colchicine on each component of the composite primary outcome.<br>3. To investigate the effect of colchicine on fatal and non-fatal ischaemic stroke combined.<br>4. To investigate the effect of colchicine on recurrent disabling and non-disabling ischaemic stroke.<br>5. To investigate the effect of colchicine on late disability, compared with usual care.<br>6. To assess whether the effect of treatment on the primary outcome is materially different among different categories of patient defined at baseline.<br>7. To investigate the effect of colchicine on direct health care costs, adjusted for quality-adjusted life year

Inclusion criteria	To be eligible for inclusion, each subject must meet each of the following criteria at the screening assessment and baseline visit:<br><br>1. Written informed consent consistent with ICH-GCP guidelines and local laws signed prior to all trial-related procedures.<br><br>2. Age 40 years or greater<br><br>3. Patient has had either; -<br>• An ischaemic stroke without major disability (modified Rankin score *3 or less*) **<br>• ***or***<br>• ***a*** high-risk TIA*<br><br>AND<br>A brain CT or MRI has excluded primary intracranial haemorrhage<br><br>AND<br>The stroke/TIA has occurred more than 72 hours before randomisation AND no more than 28 days prior to randomisation<br><br>• **Retinal infarction due to retinal artery occlusion is allowed<br><br>• **High-risk TIA is defined as transient focal neurological symptoms of presumed vascular cause with, in addition, one or more of the following criteria:*<br>*(a) ABCD2 score 4 or more, with motor or speech symptoms (dysarthria or dysphasia)*<br>*(b) DWI hyperintensity on acute MRI*<br>*(c) Stenosis (lumen narrowing of 50% or greater on ultrasound, MRA, CTA, or invasive angiography) of the internal carotid, vertebral, middle cerebral, anterior cerebral, or basilar artery in the arterial territory consistent with symptoms*<br><br>4. Qualifying stroke/TIA probably caused by large artery stenosis, small artery occlusion (lacunar stroke), or cryptogenic embolism, with cardiac embolism or other defined stroke mechanism deemed unlikely in the opinion of the treating physician.<br><br>5. eGFR greater than or equal to 50 ml/min.<br><br>6. In the opinion of the treating physician, patient is medically-stable, capable of participating in a randomised trial, and willing to attend follow-up.

Key exclusion criteria	1. Stroke/TIA, probably caused by identified atrial fibrillation (permanent or paroxysmal), in the opinion of the treating physician.<br><br>2. stroke/TIA probably caused by other identified cardiac source (intra-cardiac thrombus, endocarditis, metallic heart valve, low ejection fraction <30%)<br><br>3. Stroke/TIA caused by dissection, endocarditis, paradoxical embolism, drug use, venous thrombosis, carotid or cardiac surgery, hypercoagulability states, migraine, or inherited cerebrovascular disorders.<br><br>4. History of myopathy or myalgias with raised creatine kinase (CK) on statin therapy.<br><br>5. Blood dyscrasia (haemoglobin <10g/dL platelet count <150 x10^{9}/L, white cell count <4 x10^{9}/L)<br><br>6. Impaired hepatic function (transaminases [ALT and/or AST] greater than twice upper limit of normal (ULN)<br><br>7. Concurrent treatment with colchicine contraindicated drugs:- CYP3A4 inhibitors (clarithromycin, erythromycin, telithromycin, other macrolide antibiotics, ketoconazole, itraconazole, voriconazole, ritonavir, atazanavir, indinavir, other HIV protease inhibitors, verapamil, diltiazem, quinidine, digoxin, tolbutamide, disulfiram) or P- gp inhibitors (cyclosporine) at randomisation.<br><br>8. Symptomatic peripheral neuropathy and pre-existing progressive neuromuscular disease<br><br>9. Inflammatory bowel disease (Crohn's or ulcerative colitis) or chronic diarrhoea.<br><br>10. Dementia, sufficient to impair independence in basic activities of daily living.<br><br>11. Active malignancy, known hepatitis B or C, or HIV infection.<br><br>12. Impaired swallow preventing oral administration of Colchicine<br><br>13. History of poor medication compliance.<br><br>14. Unlikely to comply with study procedures due to severe or fatal comorbid illness or other factor (e.g. inability to travel for follow up visits), in opinion of randomising physician.<br><br>15. Women of childbearing potential (WCBP), or pregnant or are breastfeeding, are not eligible to participate in this study.<br>A woman of childbearing potential is a woman who:<br>- has not had surgery to remove the uterus and ovaries<br>- has had menstrual periods at any time in the preceding 24 consecutive months<br>- Menstrual periods interrupted due to cancer chemotherapy treatment are considered WCBP as this may still allow conception.

	Pregnancy is considered highly unlikely during the trial because women of childbearing potential are excluded. However, in the unlikely event that a woman in the trial becomes pregnant, pregnancy information will be collected.<br><br>16. Patient concurrently participating in another clinical trial with an investigational drug or device, or use of investigational drug within 30 days of the Screening visit or 5 half-lives before the screening visit (whichever is longer)<br><br>17.Known allergy or sensitivity to colchicine.<br><br>18. Requirement for colchicine therapy for treatment of acute gout, gout prevention, or other rheumatological disorder<br><br>19. Requirement for chronic daily immunosuppressants, oral steroids, or non-steroidal anti-inflammatory drugs (NSAIDs)
Test product, dose and mode of administration	Low-dose colchicine, 0.5mg/day, oral administration
Intervention arm	Low-dose colchicine, plus usual care (defined below)
Control arm	Usual care only, defined as anti-platelet, lipid-lowering, and anti-hypertensive treatment and lifestyle advice (smoking cessation, diet and physical activity), as deemed appropriate by the treating clinician.
Duration of treatment	Median approximately 36 months (approximate range 24-84 months)
Statistical methods	<u>Primary analysis</u><br>Primary efficacy analysis will be by intention to treat, comparing time to primary outcome event in colchicine-treated and usual-care groups via a log-rank test.<br><br><u>Secondary analyses</u><br>Pre-specified secondary analyses will be on-treatment (defined as any patient who received allocated intervention and remained compliant beyond one month), and individual analyses of secondary outcomes
Sample size	3,154 subjects

5 ABBREVIATIONS

ABCB1	ATP-binding cassette sub-family B member 1
AE	Adverse event
ALT	Alanine Aminotransferase
AR	Adverse reaction
ASCO	[A for atherosclerosis, S for small vessel disease, C for cardiac source, O for other cause]
BUN	Blood Urea Nitrogen
^{o}C	Degrees Celsius
CA	Competent authority
CADASIL	Cerebral Autosomal-Dominant Arteriopathy with Subcortical Infarcts and Leukoencephalopathy
CI	Chief investigator/Co-ordinating investigator
CIRT	Cardiovascular Inflammation Reduction Trial
CK	Creatine kinase
CKD-EPI	Chronic Kidney Disease Epidemiology Collaboration
CRA	Clinical research associate
CRF	Case report form
CRO	Contract research organisation
CRP	C- reactive protein
CT	Computed Tomography
CTA	Clinical trial authorisation
CTA	Computed Tomographic Angiography
CXR	Chest x-ray
CYP3A4	Cytochrome P450 3A4
DMC	Data Monitoring Committee
DWI	Diffusion-Weighted Imaging
ECG	Electrocardiogram
e-CRF	Electronic case report form
EU	European Union
EQ5D	EuroQol 5D
FDA	Food and Drug Administration
FDG	Fluorodeoxyglucose
FMF	Familial Mediterranean Fever
GCP	Good Clinical Practice
GFR	Glomerular Filtration Rate
GI	Gastrointestinal
GP	General Practitioner
HDL	High Density Lipoprotein
HIV	Human Immunodeficiency Virus
HPRA	Health Products Regulatory Authority
HSE	Health Service Executive
IB	Investigators brochure
ICF	Informed consent form
ICH	International Conference on Harmonisation
IL	Interleukin
IMP	Investigational medicinal products
IMPD	Investigational medicinal product dossier

IUD	intrauterine device (IUD),
IUS	intrauterine hormone-releasing system (),
IVRS	Interactive Voice Randomisation Service/
IWRS	Interactive Web Randomisation Service
LDL	Low Density Lipoprotein
mg	Milligrams
MI	Myocardial Infarction
Min	Minute
mL	Millilitre
MMPs	Matrix Metalloproteinases
MOCA	Montreal Cognitive Assessment
MRA	Magnetic Resonance Angiography
MDRD	Modification of Diet in Renal Disease
MRI	Magnetic Resonance Imaging
MRS	Modified Rankin Scale
NIH	National Institutes of Health
NLRP	Nucleotide-binding leucine-rich repeat-containing pyrin Receptor
OAC	Outcome Adjudication Committee
PET	Positron Emission Tomography
PGP	P-Glycoprotein
PI	Principal investigator
PIL	Patient/subject information leaflet
PROBE	Prospective Randomised Open-Label Blinded-Endpoint
P450	Cytochrome P450
QALYs	Quality Adjusted Life Years
REC	Research ethics committee
ROI	Republic of Ireland
SAE	Serious adverse event
SAR	Serious adverse reaction
SPC	Summary of product characteristics
SOP	Standard operating procedure
SUSAR	Suspected unexpected serious adverse reaction
TIA	Transient Ischaemic Attack
TOAST	Trial of Org 10172 in Acute Stroke Treatment.
TNF	Tumor-Necrosis Factor α
TSC	Trial Steering Committee
WBC	White Blood Cells
WHO	World Health Organisation
ULN	Upper Level of normal
US	United States

6 INTRODUCTION

6.1 Background

<u>Importance of stroke:</u>

The World Health Organisation (WHO) estimates that cardiovascular diseases are the leading cause of death globally, causing 17.3 million deaths in 2008. Of these, stroke was the second leading cause of global death and a major cause of healthcare costs[1-3].

<u>Importance of atherosclerosis in cerebrovascular disease:</u>

Population-based studies by our group and others have reported ipsilateral carotid atherosclerosis (≥50% arterial lumen narrowing) in up to 20% of ischaemic stroke patients,[4] with intracranial atherosclerosis in a further 8-12%. Athero-thrombosis in the arterial tree is also likely to account for a substantial proportion of the 30-40% of patients with stroke in whom no direct mechanism is identified. For example, 73% of patients in the North Dublin Population Stroke Study with TOAST-classified stroke of unidentified etiology had evidence of aortic or cranio-cervical atherosclerosis defined by the ASCO classification.[5]

In addition, disease of cerebral small vessels (arteries and arterioles) accounts for up to 20% of stroke and is a major contributor to dementia. Although the pathophysiology of cerebral ischaemic small vessel disease is varied and not fully understood, existing data suggest two common mechanisms:[6] (1) micro-atheroma of larger penetrating arterioles, causing single, frequently symptomatic acute lacunar infarcts, and (2) lipohyalinosis ('arteriolosclerosis'), more frequently associated with diffuse white matter hyperintensity on neuroimaging and neuropathological evidence of cerebral demyelination.

After exclusion of stroke due to a defined cardio-embolic source (eg. atrial fibrillation) or other identified mechanism (eg. carotid dissection), atherosclerosis of the aorta, cervical, or intracranial large or small arteries is a major pathophysiological mechanism underlying most ischemic stroke. Randomised trials of statins and antiplatelet agents (eg. SPARCL, CHANCE) have demonstrated benefit of targeting plaque-related lipid accumulation and platelet activation in non-cardioembolic stroke to prevent recurrent vascular events.

<u>High risk of recurrent vascular events in stroke survivors:</u>

Despite high rates of optimal medical and surgical treatment, we and others have demonstrated high risk of recurrent stroke associated with atherosclerosis of the internal carotid and intracranial arteries.[7] A recent systematic review reported a cumulative pooled recurrent stroke risk of 11.1% at one year and 26.4% at 5 years.[8] The risk of myocardial infarction and vascular death is also substantial in long-term stroke/TIA survivors. In a systematic review (39 studies, 65,996 patients), Touze found a 2.1%/year rate of non-stroke vascular death and 0.9%/year risk of non-fatal Myocardial Infarction (MI) in stroke survivors.[9]

<u>Importance of inflammation:</u>

Accumulating evidence indicates that inflammation is of key importance in the pathophysiology of atherosclerotic plaque destabilisation and thrombo-embolism[10,11]. The central inflammatory pathway is characterised by the key cytokines interleukin-1, interleukin-6 [IL-6], and tumor-necrosis factor α [TNF-α]. Epidemiological studies have consistently

described dose-dependent associations between ‘downstream’ markers of this pathway (e.g. C- reactive protein [CRP]) and ‘up-stream’ markers (eg. IL-6, TNF-α]) and vascular disease, including stroke.

Mendelian randomisation studies have shown that polymorphisms in the IL-6 receptor are associated with lower CRP and reduced vascular risk. In the JUPITER trial, rosuvastatin reduced the rate of vascular events in patients with low LDL but high CRP at entry, with benefit proportionate to the degree of CRP reduction.

In large-artery atherosclerosis, plaque inflammatory cells (mainly monocyte-macrophages), are increasingly recognised as key mediators of lipid oxidation, plaque remodelling, smooth muscle cell apoptosis, loss of extracellular matrix integrity via release of collagenolytic matrix metalloproteinases and other proteolytic enzymes, leading to fibrous cap rupture and thrombo-embolism.

Tawakol[12] reported high FDG uptake on positron emission tomography (PET) in symptomatic carotid plaque, with high (r=0.89) correlation with plaque macrophage density. We have shown that carotid plaque inflammation related FDG uptake predicts stroke recurrence independently of stenosis[13].

In cerebral small artery disease, available data indicate that inflammation may be an important mediator of lacunar stroke and arteriolar pathology. In patients with small artery disease, increased inflammatory cells (macrophages, activated microglia) expressing matrix metalloproteinases (MMPs) are present around affected arterioles and ischemic demyelination, and MMP-9 is increased in cerebrospinal fluid[14,15]. In SPARCL, patients with lacunar stroke randomised to atorvastatin lipid-lowering therapy had similar reduction in stroke and coronary events as those with large-artery atherosclerosis[16]. In lacunar stroke patients enrolled in the SPS3 trial; baseline CRP independently predicted recurrent stroke and vascular events[17].

Recent laboratory and clinical-pathological studies have shown that cholesterol crystals form in atherosclerotic plaques, and may activate the nucleotide-binding leucine-rich repeat-containing pyrin receptor (NLRP) inflammasome, an intracellular protein complex which promotes IL-1β expression in response to crystal stimulation, leading to elevated IL-6 and CRP[20-22]. These important observations provide direct evidence linking plaque lipid metabolism and inflammation.

Why is this trial needed now?

Few completed trials have directly tested the benefit of anti-inflammatory agents for vascular risk reduction. Small studies have demonstrated reduction in inflammatory blood and imaging surrogate-markers such as plaque FDG uptake in a dose-dependent manner by statins possibly mediated by an anti-inflammatory effect[18,19]. The LoDoCo1 trial demonstrated a 66% relative risk reduction in cardiovascular events in patients with stable coronary disease treated with low-dose colchicine, plus anti-platelet agents and statins, compared with usual care.

However, several trials are currently under way[10]. In CANTOS, canukinumab (an interleukin-1β antagonist) is under evaluation in 10,000 patients with stable coronary disease. The National Institutes of Health (NIH) funded Cardiovascular Inflammation Reduction Trial (CIRT) is comparing low-dose weekly methotrexate with placebo plus recommended care in 7,000 patients with coronary disease and diabetes or metabolic syndrome. In Australia, the LoDoCo2 trial is testing low-dose colchicine for prevention of vascular events in 3,000 patients with stable coronary syndromes. Other trials have recently targeted inflammatory

pathways (eg. LpPLA2) unrelated to the IL1-IL6-TNFα pathway (STABILITY, VISTA, SOLID-TIMI52).

6.2 Description of Colchicine and Rationale for the study

Overview:

The investigational product to be studied is low-dose colchicine, 0.5mg daily, taken by mouth for a median duration of approximately 36 months (range 24-84 months). In Europe, colchicine is registered and marketed in a number of countries by several pharmaceutical companies in generic forms (usually 0.5mg and 1mg tablets, 0.6mg tablets in some countries). The US FDA also approved single ingredient oral colchicine (0.6mg tablet) in 2009.

Pharmacokinetics – absorption, distribution, metabolism, excretion:

Colchicine has been used for many years for the treatment of acute gout and other inflammatory and arthritic conditions. Derived originally from the Autumn Crocus (Colchicium autumnale), colchicine is readily absorbed after oral administration in the jejunum and ileum, by a P-glycoprotein (ABCB1-transporter) dependent process. It undergoes significant 1st- pass hepatic metabolism, resulting in oral bioavailability averaging 45% (range 24-88%) in healthy volunteers. It is excreted primarily (80-90%) via biliary secretion (via the cytochrome P450 CYPA34 system), and also partly by the renal route (10-20%), and via intestinal epithelium.[23]

Mechanism:

Its primary cellular action is binding to α- and β-tubulin proteins, which are highly expressed in neutrophils and monocyte-macrophage inflammatory cells. It has multiple anti- inflammatory properties including inhibition of microtubule polymerization, with inhibition of E-selectin mediated leucocyte rolling and endothelial adhesion, and leucocyte motility, phagocytosis, and cytokine secretion. In vitro, colchicine inhibits crystal-induced activation of the NLRP inflammasome, possibly via inhibition of microtubule polymerisation, which is a pre-requisite for inflammasome assembly. This leads to inhibition of proteolytic cleavage of pro-IL1β by caspase-1, leading to reduced secretion of active interleukin-1β from monocytes and macrophages.

Rationale for use in atherosclerotic vascular disease:

As described above, accumulating evidence indicates that inflammation is a key process in the pathophysiology of atherosclerosis, coronary disease, and stroke. Non-randomised data report lower rates of coronary disease in gout and Familial Mediterranean Fever (FMF) patients treated with long-term low-dose colchicine therapy compared to colchicine-untreated patients.[24]

In stable coronary patients treated with statins and antiplatelet agents with elevated CRP, a 4-week treatment with low-dose colchicine was associated with significant reduction in CRP compared with controls, providing proof-of-concept that colchicine could impair inflammation in patients with atherosclerotic vascular disease.[25]

Following this study, one of our collaborators (SM Nidorf) demonstrated 66% reduced risk of recurrent vascular events in 532 patients with stable coronary disease randomised to low-dose colchicine (0.5mg/day) compared with usual care.[26] This large benefit was observed despite high (>90%) rates of statin and antiplatelet treatment in colchicine and control arms. At present, a large (3,000 patients) placebo-controlled randomised trial of low-dose (0.5mg/day) colchicine (LoDoCo2) is under way in Australia to independently validate these findings in stable coronary patients.

Recent trials have also demonstrated benefit of colchicine at a dose of 0.5-1mg/day for prevention of recurrent pericarditis.[27] More recently, in a trial of patients with acute ST-elevation myocardial infarction, colchicine (loading dose 2mg, followed by 0.5-1mg/day for 5 days) caused lower creatine kinase (p<0.001), lower MRI infarct size (p=0.019) and lower relative infarct size (p=0.034) compared with placebo.

While no systematic Cochrane reviews of colchicine for vascular prevention exist, in a recent review Ridker and Luscher stated 'large-scale….trials of colchicine in secondary prevention are warranted'.[10]

The underlying pathophysiology of recurrent vascular events in patients with non-cardioembolic stroke is likely to resemble that in patients with coronary artery disease. Therefore, we have selected the same low colchicine dose (0.5mg/day) taken by mouth, as has already shown efficacy and safety in the LoDoCo1 trial.

<u>Safety:</u>

The safety profile of colchicine is highly dose dependent. According to the UK SPC and FDA SPC, the most commonly reported adverse reaction in clinical trials of colchicine for gout prophylaxis and acute treatment was diarrhoea. Less common gastrointestinal adverse events were nausea, abdominal pain, and vomiting. These effects were far more common at high colchicine doses (up to 4.8mg daily) compared with intermediate doses (1.8mg daily). Other adverse events reported at a frequency of 2% or greater in studies of patients taking high and intermediate doses (1.8mg and 4.8mg) of colchicine included nervous system disorders, headache, and pharyngolaryngeal pain.

Per the FDA SPC, excessive accumulation or overdose of colchicine has been associated with a range of other adverse events, which are 'generally reversible upon temporarily interrupting treatment or lowering the dose'. These include neuropathy, alopecia, rash, myelosuppression, elevated transaminases (ALT and/or AST), myopathy, muscle pain, rhabdomyolysis, azoospermia, oligospermia. Serious toxic reactions associated with overdosage include myelosuppression, disseminated intravascular coagulopathy, cardiac toxicity, central nervous system toxicity, and death.

Low-dose colchicine has been used safely for many years for prevention of inflammatory complications of FMF and Bechet's disease. In contrast to the higher doses of colchicine frequently used for treatment of acute gout, low-dose (0.5mg/day) colchicine has been used in recent cardiovascular trials with excellent safety profiles. In 42 patients assigned to 0.5-1.0mg daily in the CORE trial (mean follow up 20 months), no serious adverse events occurred[27]. Mild diarrhoea developed in 3 patients, which quickly resolved on stopping or lowering the dose. In the LoDoCo1 trial, among 282 patients assigned 0.5mg colchicine daily (mean age 66.5 years, mean follow up 36 months), the overall withdrawal rate was 16%, similar to recent trials of statins and dabigatran for stroke prevention[26]. Diarrhoea and other GI adverse effects occurred in 13%, myalgia/myositis in 1%, with rash, itch, alopecia, and 'peripheral neuritis' each in 1 patient.

Adverse effects are more likely in patients with moderate-to-severe renal failure (creatinine clearance <50mL/min), hepatic failure/cirrhosis, or those taking CYP3A4 inhibitors (macrolide antibiotics, HIV protease inhibitors, itraconazole, ketoconazole, diltiazem, verapamil, grapefruit juice) or P-Glycoprotein (PGP) inhibitors (macrolide antibiotics, cyclosporine). Such patients will be excluded from the trial. If a short course of one of these agents (e.g. clarithromycin) is required, a dose interruption of colchicine will be allowed during treatment. Five cases of myopathy have been reported in the literature in patients co- administered statins with colchicine. Careful monitoring will be performed for myopathic symptoms in such patients, and they will be instructed to report significant myalgias to study personnel.

Colchicine crosses the placenta and is secreted into breast milk of nursing mothers, with unknown effects upon the developing foetus and infant. Pre-menopausal women will be excluded from entry into the trial.

7 STUDY OBJECTIVE

7.1 Primary objective

To investigate the efficacy of low dose colchicine (0.5mg/day) plus usual care (antiplatelet, lipid-lowering, antihypertensive treatment, and appropriate lifestyle advice) compared with usual care alone to prevent non-fatal recurrent ischaemic stroke, myocardial infarction, cardiac arrest, hospitalization for unstable angina and vascular death after ischaemic stroke or transient ischaemic attack (TIA) not caused by cardiac embolism or other defined causes unrelated to atherosclerosis.

7.2 Secondary objectives

1. To investigate the safety of low dose colchicine (0.5mg/day) plus usual care (antiplatelet, lipid-lowering, antihypertensive treatment, and appropriate lifestyle advice) compared with usual care alone.
2. To investigate the effect of colchicine on each component of the composite primary outcome measure.
3. To investigate the effect of colchicine on fatal and non-fatal ischaemic stroke combined.
4. To investigate the effect of colchicine on recurrent disabling and non-disabling ischaemic stroke.
5. To investigate the effect of colchicine on late disability, compared with usual care.
6. To assess whether the effect of treatment on the primary outcome is materially different among different categories of patient defined at baseline.
7. To investigate the effect of colchicine on direct health care costs, adjusted for quality-adjusted life years.

7.3 Exploratory objectives

1. Investigation of the relationship between colchicine therapy and cognition assessed by the Montreal Cognitive Assessment (MOCA, Appendix 3).
2. Investigation of the relationship between colchicine therapy and patient reported quality of life, measured by EQ5D (Appendix 5).
3. Investigation of the relationship between colchicine therapy, outcome, and early CRP.
4. Investigation of the relationship between colchicine therapy and the cumulative number of ischaemic events as components of the primary outcome cluster during the duration of the trial.

8 DEFINITION OF OUTCOME MEASURES

8.1 Primary outcome measures

The primary efficacy outcome measure will be time to the first occurrence of non-fatal recurrent ischaemic stroke, non-fatal myocardial infarction, non-fatal cardiac arrest, hospitalization for unstable angina or vascular death.

Events confirmed through centralised adjudication to meet protocol-defined primary outcome criteria, will be included in the analyses of the number of occurrences of the composite primary outcome for the respective treatment group.

The components of the primary composite efficacy outcome measure are defined below:

8.1.1 Non-fatal ischaemic stroke: defined as one of the following:

(a) A new focal neurological deficit, presumed due to cerebrovascular disease, persisting beyond 24 hours, without intracerebral haemorrhage or other mimic condition (eg. abcess, tumour, subdural haematoma) on brain CT or MRI.

(b) Brain imaging is strongly recommended for evaluation of recurrent stroke events. If brain imaging is not performed, but the focal neurological deficit is acute in onset, persists beyond 24 hours, and is consistent with stroke in the opinion of the Site Investigator/Outcomes Committee, it will be classified as non-fatal ischaemic stroke

(c) If acute new focal symptoms/signs last less than 24 hours but If brain CT or MRI demonstrates acute ischaemic change, (i.e. consistent with the ‘tissue definition’ of TIA).

Note: In patients with symptom duration less than 24 hours, in whom brain CT/MRI are normal or not performed, they will be categorised as ‘TIA’ and not counted as stroke.

(d) Retinal infarction, confirmed by an ophthalmologist.
(e) Spinal cord infarction, with mimic conditions excluded by spinal MRI.

8.1.2 Non-fatal myocardial infarction: defined according to the 3rd Universal Definition of MI criteria (Appendix 6)

8.1.3 Non-fatal cardiac arrest: defined as recovery from sudden collapse, with ECG rhythm-strip verified cardiac asystole, ventricular tachycardia, or ventricular fibrillation

8.1.4 Hospitalization for Unstable Angina: TIMI definition (see Appendix 6)

8.1.5 Vascular death: Defined as death caused by recurrent ischaemic stroke within the previous 30 days or sudden death due to verified cardiac causes (cardiac arrest, myocardial infarction (as defined above or on autopsy), without other identified cause. Ischaemic stroke will be defined as detailed in section 8.1.1 above.

8.2 Secondary Outcomes

8.2.1 Safety

The following safety outcomes will be compared between colchicine-treated and usual care groups:

i. Adverse events (non-serious and serious)
ii. Gastrointestinal (vomiting, nausea, diarrhoea)
iii. Myalgia requiring discontinuation of study medication
iv. Myopathy (defined as muscle pain or weakness associated with creatine kinase 2 or more times greater than the upper limit of normal (ULN))
v. Hepatic impairment (transaminases (AST or ALT) ≥2 ULN)
vi. Myelosuppression (defined per NIH Common Toxicity Criteria as at least Grade 2 suppression of circulating blood counts; ie. haemoglobin less than 10 and greater than 8 g/dL in the absence of major bleeding;absolute neutrophil count <1.5 - 1.0 x 10^9/L;platelet count <75.0 - 50.0 x 10^9/L)
vii. Moderate or severe renal impairment, defined as glomerular filtration rate (GFR) less than 50 ml/min/1.73m^2 on two measures at least 3 months apart
viii. Peripheral neuropathy, defined as new or worsened symptoms of numbness, parasthesiae, burning or weakness in the extremities, with confirmation on nerve conduction studies
ix. Rash, itch, or alopecia
x. Major haemorrhage, per International Society on Thrombosis and Haemostasis classification. This includes fatal and non-fatal intracranial haemorrhage. (Although colchicine has not been associated with adverse effects on platelet function or coagulation, we will record major haemorrhage rates)
xi. All cause-fatality

8.2.2 Components of composite primary outcome measure

The effect of colchicine on each of the components of the primary composite outcome measure will be analysed separately.

8.2.3 Recurrent fatal or non-fatal ischaemic stroke

Comparison of fatal plus non-fatal ischaemic stroke between colchicine and usual care arms will be performed. Fatal and non-fatal ischaemic stroke will be defined as in section 8.1.1.

8.2.4 Recurrent disabling/non-disabling ischaemic stroke

Comparison of rates of recurrent disabling ischaemic stroke (modified Rankin score 3-5) and recurrent non-disabling ischaemic stroke (modified Rankin score 0-2) between colchicine and usual care arms will be performed.

8.2.5 Disability

Comparison of disability in colchicine-treated and usual care groups will be assessed by modified Rankin score (shift analysis and proportion with no, mild, or moderate disability, defined as Rankin score of 0-2).

8.2.6 Treatment effect interaction

The effect of colchicine treatment on the primary outcome stratified by categories of key baseline variables (eg. age, gender, large artery stenosis) will be assessed.

8.2.7 Health economic outcomes

The effect of colchicine treatment on direct cumulative costs of health resource utilisation related to Quality Adjusted Life Years (QALYs) during the trial will be assessed.

8.3 Exploratory outcome measures

8.3.1 Cognition

Cognition at baseline and end of study will be measured using the Montreal Cognitive Assessment, [MOCA, Appendix 3] and compared between colchicine and usual care groups.

8.3.2 Quality of life

Health-related quality of life (self-reported) will be measured and compared using EuroQoL (EQ5D-5L) (Appendix 5).

8.3.3 CRP

Associations between colchicine treatment effect and baseline CRP will be analysed.

8.3.4 Cumulative number of ischaemic events

The relationship between colchicine therapy and the cumulative total number of component events in the primary outcome cluster detected over the duration of the trial will be investigated.

9 TRIAL DESIGN

9.1 General considerations

CONVINCE is a randomised, open-label, blinded endpoint-assessed, parallel group Phase 3 clinical trial, comparing low-dose colchicine plus usual care to usual care alone for prevention of recurrent non-fatal ischaemic stroke, myocardial infarction and cardiac arrest, hospitalization for unstable angina and vascular death after ischaemic stroke or TIA, not caused by cardiac embolism or other defined mechanisms unrelated to atherosclerosis.

This multinational trial will initially be conducted in Europe, and may then be expanded to additional European countries, North America, and/or Australia, over the recruitment period.

The trial will be conducted in two steps or stages, with a review at the end of the first stage, as follows:

- **Vanguard stage:** The first stage will be a Vanguard stage, during which it is planned to recruit approximately 265 patients at circa. 24 sites in Europe. This sample is selected as it represents 10% of the total original sample size of 2,623 study subjects. At this point, the DMC independent statistician will perform an end-of- Vanguard review of data pertaining to safety/tolerability, adherence, and the accuracy of baseline assumptions relating to outcome rates. The independent statistician will be blinded to treatment assignment and no comparison of efficacy outcomes will be performed. The DMC will review this end-of-Vanguard review performed by the independent statistician and will make a recommendation to the Trial Steering Committee relating to study modification, continuation, or termination. In addition, the TSC will review the effectiveness of study procedures and recruitment rates at this point and will consider the recommendation provided by the DMC. All decision making with respect to trial continuation will be made by the Trial Steering Committee without reference to the unblinded efficacy data and hence no 'spending of p values' will occur. The TSC will decide whether to proceed to the next stage (with protocol amendments if required) or to stop the trial at that point.

- **Full Trial stage:** The next stage is called the Full Trial stage. This is expected to proceed after the outcome of the Vanguard stage review. Recruitment of a further

2,889 patients (approximately) will be performed at up to 200 hospital sites (total re-estimated sample size 3,154). The entire sample size for the trial includes the 265 patients recruited in the Vanguard Stage plus the 2,889 recruited in the Full Trial Stage. Following close of recruitment, an estimated further two years of follow-up will then take place. Including patients recruited in the Vanguard Stage, the estimated longest follow-up period will be 84 months and shortest will be 24 months (median approximately 36 months). Follow up for a longer period may be performed if recommended by the DMC and/or TSC.

Figure 1: Study Schema

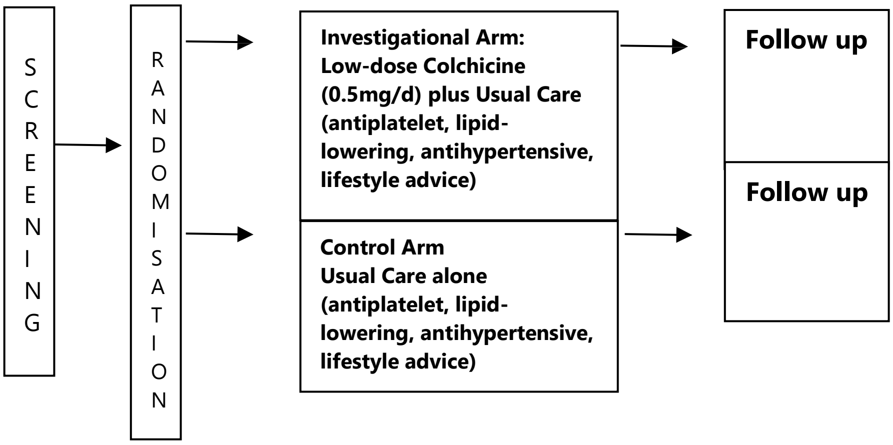

Subjects will be eligible if deemed clinically stable by their treating physician in the period between 72 hours and 28 days after a new ischaemic stroke causing mild-to-moderate disability (modified Rankin score 0-3) or high-risk TIA. They will be identified in hospital inpatient settings and outpatient clinics, informed consent obtained, randomised to one of 2 study arms and followed for an estimated 24-84 months. Follow-up visits will be conducted as described below.

Visit Number	-	-	1	2	3	4	5	6	7,9,11, 13, 15	8, 10, 12, 14	End of Trial (EOT)
**Visit Name**	**Screening**	**Baseline**	**Week 4**	**Week 12**	**Week 26**	**Week 52**	**Week 78**	**Week 104**	**Week 130, 182, 234, 286, 338**	**Week 156, 208, 260, 312**	**Week 364**
**# Days Prior to or Post Randomisation**	**0 to 28 days prior to randomisation**	**0**	**28 days**	**90 days**	**½ year**	**1 year**	**18 months**	**2 years**	**2.5, 3.5, 4.5, 5.5, 6.5 years**	**3, 4, 5, 6, years**	**7 years**
**Visit Window Allowed (days). Longer window allowed during COVID**	**See above**	**0**	**± 7**	**± 7**	**± 7**	**± 7**	**± 7**	**± 7**	**± 7**	**± 7**	**± 7**
* ϒ ECG (from usual care)	X										
* ϒ Brain CT or MRI (from usual care)	X										
* ϒ Hgb, WBC, Platelets, eGFR, ALT and/or AST, Creatinine (from usual care, <28d pre-randomisation)	X										
Inclusion/Exclusion criteria	X	X									
Informed Consent	X										
Modified Rankin Score	X		X	X	X	X	X	X	X	X	X
ABCD2 (high risk TIA only)	X										
Relevant Medical/Surgical History*		X									
Weight, Height		X									
Vital Signs (Pulse and BP after 5 min) (not mandatory during remote follow up visit)		X	X		X	X	X	X	X	X	X
Relevant Prior and Concomitant Medications		X						X			
Concomitant Contraindicated Meds Check		X	X	X	X	X	X	X	X	X	X
MOCA (not mandatory during remote follow up visit)		X						X			
EuroQol-5D-5L		X						X			
NIHSS (not mandatory, record if performed)		X									
* [a] Lipids, CK, CRP (within 28 days of randomisation, from usual care labs, not mandatory)		X									
* [a] Other stroke/TIA tests (eg, echo, holter) (not mandatory, record if done for usual care)		X									
Randomisation		X									
Dispense Study Colchicine (2 packs), if randomised to colchicine		X			X	X	X	X	X	X	X
Adverse event assessment			X	X	X	X	X	X	X	X	X
o Labs: CRP, B12 (within 60d of visit) (not mandatory if remote follow up visit)			X			X		X		X	
o Labs: Creatinine, eGFR, LFT (ALT and or AST), Hgb, WBC, Platelets (within 60d of visit) (not mandatory if remote follow up visit)						X		X		X	
Outcome event assessments			X	X	X	X	X	X	X	X	X
Study Medication Compliance Check			X	X	X	X	X	X	X	X	X
Occurrence of Carotid Endarterectomy, Stent, Atrial Fib			X	X	X	X	X	X	X	X	X

9.2 Selection of study population

9.2.1 Overall description of trial subjects

The trial subjects will be men and women aged 40 years or greater with recent ischaemic stroke or TIA, causing no disability or mild-to-moderate disability (modified Rankin score 0-3) at randomisation.

9.2.2 Inclusion criteria

To be eligible for inclusion, each subject must meet each of the following criteria at Screening Assessment and must continue to fulfil these criteria at enrolment into the trial (Baseline Visit).

1. Written informed consent consistent with ICH-GCP guidelines and local laws signed prior to all trial-related procedures.

2. Age 40 years or greater

3. Patient has had either; -

- An ischaemic stroke without major disability (modified Rankin score *3 or less). (Clarification - retinal infarction due to retinal artery occlusion is allowed*)

    or

a high-risk TIA*

AND
A brain CT or MRI has excluded primary intracranial haemorrhage

AND
The stroke/TIA has occurred more than 72 hours before randomisation AND no more than 28 days prior to randomisation

- **High-risk TIA is defined as transient focal neurological symptoms of presumed vascular cause with, in addition, one or more of the following criteria:*
    - *(a) ABCD2 score 4 or more, with motor or speech symptoms (dysarthria or dysphasia)*
    - *(b) DWI hyperintensity on acute MRI*
    - *(c) Stenosis (lumen narrowing of 50% or greater on ultrasound, MRA, CTA, or invasive angiography) of the internal carotid, vertebral, middle cerebral, anterior cerebral, or basilar artery in the arterial territory consistent with symptoms*

4. Qualifying stroke/TIA probably caused by large artery stenosis, small artery occlusion (lacunar stroke), or cryptogenic embolism, with cardiac embolism or other defined stroke mechanism deemed unlikely in the opinion of the treating physician.

5. eGFR greater than or equal to 50 ml/min.

6. In the opinion of the treating physician, patient is medically stable, capable of participating in a randomised trial, and willing to attend follow-up.

9.2.3 Exclusion criteria

Subjects are excluded from the study if any of the following criteria are met:

1. Stroke/TIA, probably caused by identified atrial fibrillation (permanent or paroxysmal), in the opinion of the treating physician.

2. Stroke/TIA probably caused by other identified cardiac source (intra-cardiac thrombus, endocarditis, metallic heart valve, low ejection fraction <30%),

3. Stroke/TIA caused by dissection, endocarditis, paradoxical embolism, drug use, venous thrombosis, carotid or cardiac surgery, hypercoagulability states, migraine, or inherited cerebrovascular disorders.

4. History of myopathy or myalgias with raised creatine kinase (CK) on statin therapy.

5. Blood dyscrasia (haemoglobin<10g/dL platelet count <150 x10^9/L, white cell count <4 x10^9/L)

6. Impaired hepatic function (transaminases greater than twice upper limit of normal)

7. Concurrent treatment with colchicine contraindicated drugs:- CYP3A4 inhibitors (clarithromycin, erythromycin, telithromycin, other macrolide antibiotics, ketoconazole, itraconazole, voriconazole, tolbutamide, ritonavir, atazanavir, indinavir, other HIV protease inhibitors, verapamil, diltiazem, quinidine, digoxin, disulfiram) or P-gp inhibitors (cyclosporine) at randomisation.

8. Symptomatic peripheral neuropathy and pre-existing progressive neuromuscular disease

9. Inflammatory bowel disease (Crohn's or ulcerative colitis) or chronic diarrhoea.

10. Dementia, sufficient to impair independence in basic activities of daily living.

11. Active malignancy, known hepatitis B or C, or HIV infection.

12. Impaired swallow preventing oral administration of Colchicine

13. History of poor medication compliance.

14. Unlikely to comply with study procedures due to severe or fatal comorbid illness or other factor (eg. inability to travel for follow up visits), in opinion of randomising physician.

15. Women of childbearing potential (WCBP), or pregnant or are breastfeeding, are not eligible to participate in this study. (Clarification: A woman of childbearing potential is a woman who:
    - has not had surgery to remove the uterus and ovaries
    - has had menstrual periods at any time in the preceding 24 consecutive months
    - Menstrual periods interrupted due to cancer chemotherapy treatment are considered WCBP as this may still allow conception.)

    Pregnancy is considered highly unlikely during the trial because women of childbearing potential are excluded. However, in the unlikely event that a woman in the trial becomes pregnant, pregnancy information will be collected.

16. Patient concurrently participating in another clinical trial with an investigational drug or device or use of investigational drug within 30 or 5 half-lives before the Screening visit (whichever is longer).

17. Known allergy or sensitivity to colchicine.

18. Requirement for colchicine therapy for treatment of acute gout, gout prevention, or other rheumatological disorder

19. Requirement for chronic daily immunosuppressants oral steroids, or non-steroidal anti-inflammatory drugs (NSAIDs)

9.2.4 Method of assigning Subjects to treatment groups

Randomisation:

Randomisation will be conducted using a minimisation algorithm, to ensure groups are balanced for key prognostic variables affecting recurrent stroke risk. The following mandatory variables will be included in the algorithm for randomisation:

(1) age (less than 70, 70 or greater)

(2) time since qualifying stroke/TIA (7 days or less, greater than 7 days)

(3) type of qualifying event (stroke or TIA).

Imaging of cervical and intracranial arteries is not mandatory prior to randomisation but is strongly encouraged. If information on large artery stenosis (i.e.. verified carotid, vertebral, or intracranial artery stenosis 50% or greater) is available at the time of randomisation, the algorithm will include this data to achieve balance for this variable. Baseline data will be entered at the same visit as treatment allocation, although non-mandatory data fields may be entered later.

Randomisation will take place via an Interactive Web Response System (IWRS),. Before randomisation occurs, informed consent and verification of trial eligibility of key inclusion/exclusion criteria must be performed by the site study staff.

In practice, when a patient is randomised, the patient will be allocated to active treatment, or usual care. If randomised to study medication, the patient will be instructed to take the first dose on the day of receipt of study medication, with food. The patient will be advised to take the medication once daily and in the morning. If the dose is forgotten in the morning it can be taken at a later time in the day. The patient will also be instructed never to take more than one tablet per day even if a previous day/days dosage has been missed

Blinding:

This is a Prospective Randomised Open-Label Blinded-Endpoint (PROBE) trial, similar to other recent trials of stroke prevention (eg. RE-LY) and acute treatment (eg. ESCAPE). Participants and treating physicians will be aware of treatment allocation to colchicine or usual care. Therefore, to control for bias, assessment of outcome events will be achieved by assessment of defined ‘hard’ endpoints with pre-specified objective evidence to support identification (see Section 8.1, Primary Outcome Measure). The assessment will be conducted by an Outcomes Adjudication Committee blinded to treatment allocation. The OAC will meet or teleconference regularly during the conduct of the trial.

9.2.5 Investigations prior to enrolment in the trial

Certain investigations are **mandatory** prior to entry in the trial to determine patient eligibility. These are usually performed as the standard of usual care for patients with their qualifying stroke or TIA. They are listed below ***and are required within 28 days prior to trial entry:-***

1. Brain CT or MRI
2. eGFR (by MDRD or CKD-Epi method – Appendices 10,11)
3. Liver transaminases (ALT and/or AST)
4. Hemoglobin, WBC, Platelets
5. ECG or rhythm strip (a minimum requirement to exclude atrial fibrillation)

The following investigations are <u>not mandatory</u> prior to trial entry but if they are performed please enter the results into the eCRF at the Baseline visit

1. ***Vascular imaging of the neck and intracranial arteries is not mandatory*** prior to enrolment but ***is strongly encouraged***.

2. ***Continuous cardiac monitoring (telemetry or ambulatory Holter monitoring) is not mandatory,*** If continuous cardiac monitoring is performed, a minimum duration of 20 hours is recommended.

3. ***Echocardiography is not mandatory prior to enrolment***,

4. ***Further investigations for uncommon causes of stroke*** (eg. 'dissection-protocol' MR angiography sequences, transoesaphageal echocardiography, hypercoagulability screen) are not mandatory prior to enrolment. If performed enter on the Baseline and Randomisation visit eCRF

5. ***CK, CRP, and lipid profiles are not mandatory prior to enrolment***: if they are done within 28 days of randomisation from local laboratories, they should be entered on the CRF.

6. **The NIH Stroke Scale** (Appendix 4) score (stroke patients only) is not mandatory, but if performed results will be recorded.

9.3 Study assessments and procedures

9.3.1 Schedule of Visits – Visit Timelines

In order to standardise the number of weeks between visits for all patients in the study: 'weeks' rather than 'calendar months' are being used. Therefore, for this study **the definition of a month** is 4 weeks, a year is 52 weeks, and a half year is 26 weeks. All visits for each patient are scheduled out for the entire study based on the randomisation date of each patient.

If for any reason a patient is late/too early for one visit, the subsequent planned visit is not to be adjusted: the patient should continue per the schedule planned at randomisation.

Study visits and assessments are summarised in the Task Schedule below

Baseline and follow-up assessment data will be reported to the sponsor using the eCRF. If a patient cannot attend for a visit the information can be taken over the phone with the patient and the visit completed in the e-CRF (the visit date = date of call)

9.3.1a Protocol Update: Response to COVID-19:

In response to the COVID-19 pandemic, to reduce unnecessary visits to hospital, the following procedures will apply:

1. ***Timing of follow-up visits**: All follow up visits should be completed as close as possible to the schedule outlined in the study protocol. Increased flexibility is permitted for the timing of the follow-up visit, if judged by the site team that this is a practical solution to completing the visit. If the visit is conducted outside the range +/-7 days of the scheduled date, this is not classified as a Protocol Violation.*

2. *The protocol currently allows for follow-up visits to be completed by telephone with patient supported by a family member if needed.* ***Remote visits may also be done by video-call*** *(using Skype, Zoom, or other applications).*

3. *Where possible, the End of Trial/Withdrawal Visit should be done as a face-to-face visit. Laboratory tests should be done at this visit where possible. If a face-to-face visit is not possible, it is acceptable for this visit to be done remotely without laboratory tests. In this case, a remote visit or unavailability of laboratory tests will not be classified as a Protocol Violation.*

9.3.2 Description of Study Assessments

Adverse Events:

Adverse event collection will begin at randomisation and will finish at the end-of-trial assessment (28 days after last dose of study medication).

Adverse Event Assessment

Subjects will be interviewed during each subject visit to determine if an adverse event has occurred. Specific monitoring for the following adverse events will be performed in colchicine-treated and usual care groups:

1. Any adverse events (non-serious and serious)
2. Gastrointestinal (vomiting, nausea, diarrhoea)
3. Myalgia requiring discontinuation of study medication
4. Myopathy (defined as muscle pain or weakness associated with creatine kinase 2 or more times greater than the upper limit of normal (ULN))
5. Hepatic (transaminases ≥2 ULN)
6. Myelosuppression (defined per NIH Common Toxicity Criteria as at least Grade 2 suppression of circulating blood counts, i.e. haemoglobin less than 10 and greater than 8 g/dL in the absence of major bleeding; absolute neutrophil count <1.5 - 1.0 x 10^9/L; platelet count <75.0 - 50.0 x 10^9/L)
7. Moderate or severe renal impairment, defined as GFR less than 50 ml/min/1.73m2 on two measures at least 3 months apart
8. Peripheral neuropathy, defined as new or worsened symptoms of numbness, parasthesiae, burning or weakness in the extremities, with confirmation on nerve conduction studies
9. Rash, itch, or alopecia
10. Major haemorrhage, per International Society on Thrombosis and Haemostasis classification (although colchicine has not been associated with adverse effects on platelet function or coagulation, we will record major haemorrhage rates)
11. All cause-fatality

All adverse events will be reviewed by an Investigator, who will determine the causal association between the event and colchicine. In the event of an adverse reaction, please refer to Section 11.5 for further guidance.

Concomitant Medication: The subject will be assessed at Baseline, Visit 6 (104 weeks), Visit 12 (week 260) to determine all concomitant medications that are relevant to this study group:- anti-platelets, anti-coagulants, anti-hypertensives, lipid lowering, occasional anti-inflammatory/immunosuppressant agents, anti-diabetic.

Concomitant contraindicated medication check: The subject will be asked at every visit if any medications have been taken that are contraindicated in patients taking colchicine (see section 10.7.2)

Demographics: The date of birth, gender and race will be recorded at Screening Visit

Dispense study medication: Patients randomised to study drug are dispensed colchicine at baseline, visit 3 and every subsequent visit. (no drug is dispensed at week 4 visit). See section 10.8 for COVID-19 Protocol Update, Dispensing.

ECG: It is expected that a 12-lead ECG or cardiac monitoring documented on a printed rhythm strip to exclude atrial fibrillation will have been performed as standard of care for the qualifying stroke/TIA within 28 days of randomisation. This will be reviewed at Screening to determine eligibility and recorded in the Baseline visit. Abnormal findings will be noted for clinical significance and signed by an Investigator.

Informed Consent: see screening assessment (see 9.3.3)

Inclusion/Exclusion criteria: see screening assessment (see 9.3.3)

Laboratory exams: see task schedule for visits and windows allowed for all lab tests. All blood samples collected for safety monitoring will be measured by local laboratories and destroyed by local laboratories according to their standard procedures. The protocol does not require any special storage or processing of these.

Protocol Update: Response to COVID-19:
Laboratory tests are no longer mandatory at Follow-up visit 1 (28 days) if this visit is done remotely.

*For remote follow-up visits, the screening strategy for laboratory abnormalities is changed to opportunistic screening instead of scheduled screening. This means that **blood tests for scheduled monitoring of bone marrow suppression, hepatic injury, renal failure, B12 or CRP are no longer mandatory at annual follow-up visits if such visit is done remotely**. Investigators should do these tests at the next in-person visit. If an in-person visit is not scheduled, it is acceptable to record results of these blood tests once per year if the patient has them done during a hospital visit for routine clinical care, with the date of the test. If the blood tests are not done within one year, non-completion of laboratory tests will not be classified as a Protocol Violation.*

Medical and Surgical History: Details relating to relevant significant medical and surgical history will be recorded at Baseline Visit.

Medication Compliance: The number of tablets remaining are counted at every visit excluding the telephone call at day 90 (visit 2).

Protocol Update: Response to COVID-19:

The site team will verify patient compliance with study medication by counting the number of remaining tablets during the telephone/video-call. It is acceptable for the patient to return unused medication via standard post to the study office, using a stamped addressed envelope provided by the study team. Alternative methods of return of unused medication (eg. delivery by patient's family to a study team member at the hospital door) are acceptable, if considered feasible in the opinion of the study team. Other procedures for drug accountability are unchanged.

Occurrence of Carotid Endarterectomy/Stent/Atrial Fibrillation 'since previous visit' assessment: A check is made at every visit to record if any of these have occurred.

Randomisation: see randomisation 9.2.4

Neurological and Quality of Life Scores: The ABCD2 score (TIA only), modified Rankin score all patients (see Appendix 1 for algorithm), Montreal Cognitive Assessment (MOCA), and EuroQO-5L will be recorded (see Appendices 2, 1, 3 &5). The NIH Stroke Scale (Appendix 4) score (stroke patients only) is not mandatory, but if performed results will be recorded.

Protocol Update: Response to COVID-19:

If a follow-up visit is done remotely, the modified Rankin Score can be scored remotely, using the standard mRS algorithm (Appendix 1). If the 2-year and End of Trial/Withdrawal visits are done remotely, the MOCA need not be done. This is not classified as a Protocol Violation. The EQ5D may be done by phone.

Vital Signs: Pulse and BP: Resting pulse and blood pressure measurements will be taken after the subject has been seated for at least five minutes at every visit excluding Visit 2 (phone call visit).

Protocol Update: Response to COVID-19:

Pulse and blood pressure need not be measured at remote follow up visits. This is not a Protocol Violation. Home measurements of blood pressure and/or pulse taken by the patient are acceptable.

Weight and Height will be recorded at baseline.

Outcome event assessment: Subjects will be monitored at each Study Visit for suspected outcome events (cardiac events including hospitalization for unstable angina, cardiac arrest, myocardial infarction, stroke/TIA, vascular death) which will be managed at each site by local physicians according to the standard of care. An event form for cardiac event/hospitalization/death/stroke/TIA,MI will be obtained for central adjudication, the suspected outcome will be reported via the CRF, and a decision relating to withdrawal or continuation of colchicine therapy will be made by the site study physician (for patients randomised to colchicine – see section 9.5). Pre-specified supporting documentation will be pesudo-anonymised (the only reference to the patient will be their CONVINCE Subject ID number), and provided to the Coordinating Centre team, as detailed in Appendix 9.

COVID-19 Protocol Update Summary: Follow-up visits:

**A: Preferred Option**	**Follow-up at face-to-face visit**
**B. Alternative Option permitted**	**Follow-up at remote visit by phone or video-call**

COVID-19 Protocol Update Summary: Blood tests at follow-up:

**A: Preferred Option**	**Blood tests done at 28 days and at follow-up visits at 1,2,3 years and end of trial**
**B. Alternative Option**	**If blood tests are not available at times above, they can be recorded from routine clinical care or a later follow-up study visit. If blood tests not available, no protocol violation**

9.3.3 Screening and Baseline Assessment:

Subjects will be identified by study investigators from patients admitted to acute hospitals following ischaemic stroke or TIA, and from those attending outpatient clinics.

It is expected that all evaluations required to determine subject eligibility for the trial will have been conducted as part of the routine/standard of care evaluation for the qualifying Stroke/TIA. Therefore, no additional screening tests or procedures will be required to determine patient eligibility for the trial. It is expected that screening for patients entering the trial will be identified during their in-patient stay or at return visits to clinics scheduled for routine clinical care.

Once a subject is identified as eligible, the trial will be described to him/her, and written material will be provided. Informed consent will be obtained prior to any study related procedures being undertaken. Date of screening, subject age, gender and reason for ineligibility (if subject is not eligible) will be recorded. The results of the screening evaluation must meet the inclusion/exclusion criteria for the subject to continue in the study.

The maximum duration allowed between screening and randomisation is 28 days. In cases where all data needed to determine eligibility are already available from routine/standard of care evaluations, the Screening Assessment may be performed at the time of the Baseline Visit (i.e. scheduling a separate screening visit is not required). However, if required, a separate Screening Visit may be arranged within 28 days of the Baseline Visit.

If a blood test or ECG/rhythm strip has not been performed as part of the Stroke/TIA evaluation, this may be done at the Screening Assessment to determine eligibility, after informed consent has been obtained.

The Task Schedule outlines the examinations and assessments required at all study visits and should be used as a reference guide at all visits.

9.3.3a Protocol Update: New recruitment option for patients discharged early on COVID-19 pathway:

Most patients will be recruited as outlined above. ***This remains the preferred option.*** *Some patients meet all other eligibility criteria but are rapidly discharged from hospital according to new COVID-19 pathways before 72 hours have passed since the Qualifying Event (stroke or high-risk TIA).*

In this situation, as an alternative option, the following procedure may be applied:

1. *informed consent is obtained from the patient before hospital discharge. The Screening/Baseline visit may be done at this time, before hospital discharge or may be done by phone after discharge.*
2. *As soon as possible after the 72 hour period has passed, the patient should be contacted by phone*
3. *The site team must verify that the patient is still eligible for the trial. If the Screening/Baseline visit has not been completed before discharge, it can be completed during the phone call.*
4. *Randomisation is done during the phone call*
5. *If randomised to colchicine, study drug is provided to the patient as outlined below and the usual instructions provided for storing and taking colchicine*
6. *The Screening/Baseline Form in the eCRF is completed as usual.*
7. *The MOCA need not be done. This is not classified as a Protocol Violation. The EQ5D may be done by phone (it is validated for phone use)*

Summary: Recruitment of new patients:

**A: Preferred Option**	**Consent, Screening/Baseline Visit, Randomisation and Study Drug dispensing procedures all done at face-to-face visit, as per original pre-COVID protocol**
**B. Alternative Option permitted**	**For patients discharged early due to COVID pathways: Consent done and Screening/Baseline started at face-to-face visit. Screening/Baseline, Randomisation, and Drug dispensing finished remotely post-discharge after 72 hours**

9.3.4 Summary of Assessments at Follow-Up visits, End of Study/Withdrawal Visit, Investigator support :

a) Assessments at all visits are:
Safety – adverse events, pulse and blood pressure,
Modified Rankin Score
Outcome Events
Compliance Study Medication
Contraindicated Concomitant Medications
Dispense Study Drug

b) Additional assessments particular to specific visits are:
CRP, B12: at week 4 weeks 52, 104, 156, 208, 260
Hgb, WBC, Platelets, ALT and/or AST eGFR, Creatinine: weeks 52, 104, 156, 208, 260
EuroQol-5L, MOCA, Relevant Concomitant medication weeks 104, 260

Investigator Support:
Support (email, telephone) will be available for study investigators for questions which may arise at follow-up Study Visits. This service will be via the Trial Coordinating Centre and national Lead Investigators.

End of trial/Subject Withdrawal visit:
If for any reason the patient withdraws early, or study is prematurely discontinued all patients will be given an end of trial visit. Assessments are as outlined in (a) plus (b) above. Patients who withdraw from the trial or for whom trial treatment is terminated will not be replaced.

9.4 Definition of end-of-trial

The end of trial will be the date of the last visit/telephone follow-up/home visit of the last subject.

The Trial Steering Committee will have the right at any time to terminate the study for ethical, clinical, or administrative reasons, or following a recommendation from the DMC to terminate for safety or efficacy reasons.

The end of the study will be reported to the relevant Ethics Committees/Institutional Review Boards and Regulatory Authorities within 90 days, or 15 days if the study is terminated prematurely. The investigators will inform subjects and ensure that appropriate follow-up is arranged for recruited patients, if trial termination occurs for reasons of safety concerns.

A summary report of the trial will be provided to the Ethics Committees and relevant Regulatory Authority within as per national legal requirements in participating countries.

If the study is terminated prematurely, all investigators and participating subjects will be informed within 15 working days. All patients will be scheduled for an end-of-trial visit within 28 days. If further follow-up care is required, this will be arranged as appropriate by the Site Investigators. All Ethics Committees and Regulatory Authorities will be informed in writing within 15 days of termination, and a written summary report of the trial will be provided according to locally-agreed timelines.

9.4.1 Loss to follow-up:
At least 3 documented attempts must be made to contact any subject (in writing or by phone) before a subject is deemed lost to follow-up.

9.5 Discontinuation/withdrawal, Dose interruption, and management of AF and carotid disease:

9.5.1 Discontinuation of colchicine:
Subjects have the right to voluntarily discontinue study colchicine without consequences. Colchicine may also be discontinued at the request of the Site Investigator or Sponsor in response to an Adverse Event, Adverse Reaction, pregnancy, medical condition which may jeopardise the subject’s health, or non- compliance with study procedures.

If the reason for discontinuation of study colchicine is temporary (eg. mild adverse reaction such as nausea, or requirement for a short course of antibiotic which may

interact), the subject should be encouraged to resume colchicine when deemed appropriate and safe.

In such cases, the Site Investigator may reduce the dose of colchicine to 0.25mg daily, for a period while the 0.5mg dose is being considered for reintroduction.

An interruption of colchicine for 6 months or greater is classified as permanent discontinuation.

All subjects discontinuing study colchicine permanently should be invited to return for an End-of-trial visit within 28 days. The subject will continue to receive follow-up contacts every six months (+/-7 days) to perform an outcome event assessment until end of trial. The eCRF is continued as per the existing visit schedule. An End of Trial/Early Withdrawal form is not completed if the patient remains in follow up, even if colchicine is prematurely discontinued.

Follow-up of such subjects may be conducted by in-person or by telephone. If the subject declines such visits, then follow-up may be performed by contact with family physician, hospital records, or other surrogate method (eg. named family member) as agreed with the subject.

9.5.2 Management of colchicine dose interruption/reduction

If moderate/strong CYP3A4 or P-gp inhibitors (listed in Section 10.7.2) are temporarily required during the trial, colchicine treatment should be interrupted as recommended in the SPC. If study colchicine must be interrupted for this or other reasons (eg. due to intercurrent illness), the duration of interruption will be recorded, and subjects will be requested to resume colchicine after the reason for interruption has passed. A minimum 7-day wash-out period is recommended before resuming colchicine after treatment with CYP3A4 or P-gp inhibitors.

Note: Non-steroidal anti-inflammatory drugs (NSAIDs) and steroids are not moderate/strong CYP3A4 or P-gp inhibitors. No colchicine interruption is required if a short course of these medications is required (3 weeks or less)

9.5.3 Management of atrial fibrillation

If atrial fibrillation is detected during the conduct of the trial (after randomisation), the patient should be treated according to standard of care at the site, and if deemed appropriate by the Investigator, with anticoagulant medication. The subject may continue colchicine treatment in the trial and will undergo follow-up assessments as per the study schedule.

9.5.4 Management of carotid endarterectomy or stenting

Patients scheduled for carotid surgery or stenting after their qualifying stroke/TIA may be randomised and start low-dose colchicine if assigned to this treatment arm. If carotid surgery or stenting is required during the conduct of the trial (after randomisation), the subject may continue in the trial, where deemed appropriate by the Investigator. Stroke or coronary events following carotid surgery or stenting will be recorded as outcome events.

9.6 Continuation of colchicine after a primary outcome event:

In addition to reducing the rate of first recurrent ischaemic events, colchicine may reduce the cumulative number of recurrent ischaemic events after stroke. Therefore, if a study subject taking colchicine experiences a primary outcome event, he/she may continue on

colchicine for the duration of the trial (i.e. until the end of trial as defined in section 9.4), if deemed safe and appropriate in the opinion of the Site Investigator and treating physician. Such subjects should continue to be followed as per the study schedule until the end of trial.

9.7 Continuation of follow-up assessments after a primary outcome event:

Colchicine may reduce the cumulative number of recurrent ischaemic events after stroke, and may have benefits for reduction in late disability or death after first recurrent ischaemic events.

Therefore, subjects who experience an outcome of non-fatal ischaemic stroke or coronary event should continue to have follow-up assessments until the end of trial (defined in section 9.4), as per the follow-up schedule. Follow-up should be performed regardless of whether the subject was initially randomised to colchicine or usual care, and regardless of whether the subject continues to take colchicine following the recurrent ischaemic stroke or coronary event. If visits to the hospital/clinic are not possible, follow-up may be performed by telephone call with the subject, carer, subject's physician, or family member.

10 OVERVIEW OF TRIAL MEDICATION

10.1 Description

Colchicine 0.5mg tablets, given by mouth, once daily.

10.2 Formulation, packaging and handling

Colchicine is formulated in a white to off white, round or oblong, 6 mm diameter, flat tablet with bevelled edges, debossed with “0.5” on one side. Each tablet contains 500 micrograms of colchicine. One excipient with known effect is contained per tablet – this is 59 mg of lactose monohydrate. Refer to the Summary of Product Characteristics for the full list of excipients. Packaging is in white opaque PVC/ plain push through aluminium foil blister packs containing 100 tablets per pack or bottle containing XX tablets. Original packaging will be labelled in accordance with with Annex 13 (EU Guidelines to Good Manufacturing Practice, Investigational Medicinal Products).

10.3 Storage and disposition

Colchicine tablets should be stored in the original package in order to protect from light. The tablets do not require refrigeration or temperature monitoring.

The shelf-life is a minimum of 2 years. Disposal will be performed at study sites by study staff after verification of returned tablets. There are no special requirements for disposal.

Study colchicine will be stored and locked in a secure place at the Investigator site until they are dispensed for subject use or are returned to the Sponsor.

Colchicine tablets supplied to Investigator sites for the trial are for research use only and are to be used only within the context of the trial.

10.4 Accountability of study colchicine

Study colchicine will be supplied by a licensed supplier to the Contract Research Organisation premises for over-labelling of original packaging in accordance with Annex 13 requirements. It will then be shipped directly to individual study sites by an accredited courier company. Drug supply management will be coordinated by the Contract Research Organisation and the Sponsor Coordinating Centre team.

Sites will receive a supply of study colchicine in batches, based on anticipated recruitment and re-supply requirement within the subsequent 6 months.
The status of all study medication shipments (created, in transit, arrived) will be digitally recorded and maintained on a database maintained by the Contract Research Organisation, with regular reports to the Trial Coordinating Centre. Site drug receipt and episodes of site to site transfer (if required), will be recorded on the database. Each site will maintain an inventory of colchicine received.
Unused study medication will be retrieved at each follow-up visit, and at the end of the trial, to monitor subject compliance with allocated colchicine. The Site Investigator is responsible for the control of study medication at each site.

Records will be kept of dispensing, return, and destruction of study colchicine in study logs and the eCRF at each site.

10.5 Assessment of compliance

The investigator is responsible for ensuring that study colchicine is administered in compliance with the protocol. Subject compliance will be assessed by interview at Study Visits, and by maintaining dispensing records and performing a count of returned medication. Subjects will be asked to bring all used and unused colchicine packaging at each visit. Any discrepancies noted will be followed up with the subject by the Investigator.

Significant non-compliance will be defined as failure to adhere to less than 80% of prescribed colchicine. In the event of significant non-compliance, the following remedial procedures will apply in a stepwise fashion:

1. Interview to evaluate reasons for non-compliance
2. Potential solutions to promote compliance will be offered to the patient- education regarding study medication, option to take with food
3. The option of dose reduction to 0.25mg daily for an agreed period will be offered if other solutions are not successful
4. If non-compliance is sustained, the patient will be encouraged to continue with follow-up visits as described above.

10.6 Overdose of study colchicine

Procedures for colchicine overdose will follow those outlined in the UK Summary of Product Characteristics for colchicine. The lethal dose varies widely (7 - 65 mg single dose) for adults but is generally about 20 mg. Therefore, overdose of study colchicine treatment is unlikely during the trial, given the low dose of colchicine (0.5mg daily) under study.

In the unlikely event of colchicine overdose, all patients, even in the absence of early symptoms, should be referred for immediate medical assessment.

Clinical:
Symptoms of acute overdosage may be delayed (3 hours on average): nausea, vomiting, abdominal pain, hemorrhagic gastroenteritis, volume depletion, electrolyte abnormalities, leucocytosis, hypotension in severe cases. The second phase with life threatening complications develops 24 to 72 hours after drug administration: multisystem organ dysfunction, acute renal failure, confusion, coma, ascending peripheral motor and sensory neuropathy, myocardial depression, pancytopenia, dysrhythmias, respiratory failure, consumption coagulopathy. Death may result from respiratory depression and cardiovascular collapse. If the patient survives, recovery may be accompanied by rebound leucocytosis and reversible alopecia starting about one week after the initial ingestion.

Treatment:
No antidote is available.

Treatment will focus on elimination of toxins by gastric lavage if treatment is begun early (within one hour of acute poisoning). Oral activated charcoal may be of benefit in adults who have ingested more than 0.1mg/kg bodyweight within 1 hour of presentation and in children who have ingested any amount within 1 hour of presentation. Haemodialysis has no efficacy.

Close clinical and biological monitoring in hospital environment will be performed, plus symptomatic and supportive treatment (control of respiration, maintenance of blood pressure and circulation, correction of fluid and electrolytes imbalance).

10.7 Prior and concomitant therapy

While on colchicine or usual care, information on colchicine contraindicated medications (see 10.7.2) will be taken during each study visit. Medications relevant to this patient population will be collected at baseline, week 104, week 260 (anti-platelet, anti-coagulant, anti-hypertensive, lipid lowering, occasional anti-inflammatory/immunosuppressant agents)

10.7.1 Permitted medications/non-investigational medicinal products

Patients will receive standard usual care for stroke prevention, as indicated in the judgement of treating physician, either alone or in combination with colchicine. Standard treatments for acute stroke therapy (including alteplase) and stroke prevention (anti-platelet, anti-coagulant [warfarin, dabigatran, apixaban, rivaroxaban, edoxaban, heparin and low molecular weight heparins], statins, fibrates, ezetimide, and anti-hypertensive medications are permitted in combination with low-dose colchicine. Monitoring for myopathy will be performed in patients taking statins and/or fibrates and colchicine.

10.7.2 Contra-indicated prohibited medications

As listed in the UK SPC, the following are contraindicated medications in patients co-administered colchicine therapy:

- Moderate or strong CYP3A4 inhibitors (clarithromycin, erythromycin, telithromycin, other macrolide antibiotics, ketoconazole, itraconazole, voriconazole, ritonavir, atazanavir, indinavir, other HIV protease inhibitors, verapamil, diltiazem, quinidine, digoxin, tolbutamide, disulfiram) or P-gp inhibitors (cyclosporine).
- Grapefruit juice is a moderate CYP3A4 inhibitor and should be avoided.

Subjects assigned to the colchicine arm will be given a wallet card listing contra-indicated (prohibited) medications during the trial. If a prohibited medication is required

during the study, then colchicine will be interrupted as outlined in section 9.5.2. The GP letter will also include the list of contraindicated medication.

Subjects must not participate in any other clinical trials while participating in CONVINCE.

10.8 COVID-19 Protocol Update, colchicine dispensing:
Resupply of existing patients on colchicine:

During the COVID-19 pandemic, a priority will be to ensure continuous supply of colchicine to patients in this arm of the trial. A range of options are available to ensure this:

a) Colchicine may be dispensed at a scheduled in-person visit

b) In areas where clinic visits are continuing, ***patients may be called in early by site teams*** *to be re-supplied with colchicine. If needed, the remainder of the follow-up visit may be done by phone after colchicine is dispensed.*

c) ***Drive-through dispensing is allowed****. The patient or family member drives to the outside of the research building, and site team personnel hand over study medication and receive returned packets and unused tablets. The remainder of the follow-up visit is done by phone.*

d) If patient visit or drive-through dispensing are not possible, some sites may use ***delivery of colchicine to patients****. Usually the site must have an established relationship/contract with a courier company (or similar service) to avail of this option. Costs will be reimbursed and logistics supported by the trial Project Office, where practical. Guidelines for same will be provided.*

If a temporary interruption of study colchicine occurs, study medication should be re-started as soon as possible in this situation. A Protocol Deviation form should be completed and the Project Office notified of the interruption.

Summary: Colchicine dispensing:

**A: Preferred Option**	**Dispensing at face-to-face visit as per pre-COVID protocol**
**B. Alternative Option permitted**	**Drive-through dispensing**
**C. Alternative Option permitted – Last resort**	**Delivery of colchicine to patient**

11 SAFETY AND QUALITY MONITORING AND REPORTING

11.1 Overview and reference guidance

The safety and tolerability of study colchicine will be evaluated throughout the study, including evaluation of adverse events (AEs), vital signs, physical examination findings and abnormal laboratory values at study visits. Adverse event collection will begin at randomisation and will finish at the end-of-trial assessment (28 days after last dose of study medication).

Pharmacovigilance roles, structures, and processes will be in accordance with available

regulations and guidance on adverse event/reaction reports arising from clinical trials on medicinal products for human use including but not limited to:

- EU Clinical Trial and Good Clinical Practice Directives
- Relevant ICH guidelines
- European Commission CT-3 guidance
- Relevant national guidance, as applicable

Key tasks of Site Investigators include:

- Complying with study assessments
- Recording adverse events
- Reporting adverse events to the sponsor

Key tasks of the Sponsor include:

A. Recording adverse events
B. Expedited reporting of SUSARs
C. Annual safety reporting to Ethics Committees and national regulators
D. Continuous assessment of safety and benefits of the trial, via the DMC and TSC
E. Ensuring compliance with quality standards for data management

11.2 Definitions

11.2.1 Adverse event (AE)

Any untoward medical occurrence in a patient in either arm (Usual Care or Colchicine-treated). This includes any unfavourable and unintended sign (including an abnormal laboratory finding), symptom or disease in a trial subject. *If an AE occurs in a colchicine-treated patient, it may or may not have a causal relationship with colchicine.*

11.2.2 Adverse reaction (AR)

All untoward and unintended responses to a study medicinal product (ie. either colchicine or usual care medications). The phrase 'responses to a medicinal product' means that a causal relationship between a study medication and an AE is at least a reasonable possibility.

All cases judged by either the reporting physician or the sponsor as having a reasonable suspected causal relationship (at least possible) to a study medication (colchicine or usual care) qualify as adverse reactions.

11.2.3 Serious adverse event (SAE)

A serious adverse event (SAE) is any untoward medical occurrence in a patient in either arm of the trial (Usual Care or Colchicine-treated) that meets at least one of the 6 criteria outlined below. *Note that if an SAE occurs in a colchicine-treated patient, it may or may not have a causal relationship with colchicine.*

Criteria for an SAE are that the adverse event:

1. Results in death.

Notes:

(i) Death due to recurrent ischaemic stroke, cardiac arrest, or myocardial infarction are categorised as Outcome Events and will not be reported as SAEs

to the sponsor.

(ii) Death due to the qualifying ischaemic stroke, recurrent haemorrhagic stroke, or other non-vascular causes are categorised as SAEs.

2. Is life-threatening:

A life-threatening event refers to an event in which the subject was at risk of death at the time of the event; it does not refer to an event which hypothetically might have caused death if it were more severe.

Notes:

(i) Recurrent non-fatal ischaemic stroke, myocardial infarction, or cardiac arrest which are life-threatening are categorised as Outcome Events and will not be reported as SAEs to the sponsor.

(ii) Patients must be medically stable from their qualifying stroke/TIA at the time of trial entry. Life-threatening deterioration in medical condition following randomisation which is related to the qualifying stroke/TIA is categorised as an SAE.

3. Requires hospitalisation or prolongation of existing hospitalisation.

Notes:

(i) Recurrent ischaemic stroke, myocardial infarction, unstable angina or cardiac arrest requiring, or prolonging hospitalisation are categorised as Outcome Events and will not be reported as SAEs to the sponsor

(ii) New or extended hospitalisation for medical or surgical procedures (eg. carotid endarterectomy or stenting) which are planned prior to randomisation but conducted after randomisation are not categorised as SAEs.

(iii) Hospitalisation is defined as overnight stay in hospital. The following are not defined as hospitalisation and do not need to be reported as SAEs: Visits to clinics or day hospitals, Emergency Departments, or other hospital day services (eg. minor surgical procedures); surgeries or procedures planned before randomisation but conducted after; hospital admission for an outcome event.

(iv) Note that some events associated with brief treatment in hospital may not meet the definition of hospitalisation, but may be reportable as 'important medical events in the opinion of the investigator' if they pose a risk to life or disability.

4. Results in persistent significant disability or incapacity.

Notes:

Disability caused by the qualifying stroke is not categorised as an SAE.

Disability caused by recurrent ischaemic stroke, unstable angina, myocardial infarction, or cardiac arrest is an outcome event and will not be reported as SAEs to the sponsor

5. Is a congenital anomaly or birth defect.

6. Is an important medical event, in the opinion of the Investigator. Some medical events may jeopardise the subject or may require an intervention to prevent one of the above characteristics/consequences. Such events (hereinafter referred to as 'important medical events') should also be considered as 'serious' in accordance with the definition. For example, events which require medical/surgical intervention to prevent one of the above outcomes (e.g. GI bleeding requiring endoscopy).

11.2.4 Serious adverse reaction

A reaction, in contrast to an event, is characterized by the fact that a causal relationship between the drug and the occurrence is suspected.

Any of the serious events listed above which are judged to be at least possibly related to study colchicine or usual care medications, in the opinion of the site physician investigator, and/or Chief Investigator, will be categorised as a Serious Adverse Reaction (SAR)

A SAR causally associated with study colchicine or usual care medications may be consistent with events previously described and listed in the Summary of Product Characteristics. In this situation, the SAR is considered ‘expected’.

11.2.5 Suspected unexpected serious adverse reaction (SUSAR)

A SUSAR is a serious adverse reaction, considered at least possibly related to study medications (colchicine or usual care), and is unexpected based on the following criteria:

(1) the nature of the reaction is not consistent with reactions previously described and listed in the Reference Safety Information
(2) the nature of the reaction has been previously described, but the severity of the reaction is greater than anticipated based on Reference Safety Information.

The Reference Safety Information for this trial is the Summary of Product Characteristics (SPC) for Colchicine 500microgram Tablets (PL30306/0573).

11.3 Evaluation of Adverse Events

11.3.1 Assessment of seriousness

The site investigator should make an assessment of seriousness using the criteria defined in section 9.3.2.

11.3.2 Assessment of casualty

The causality of study medication (colchicine or usual care medications) to the AE will be determined by the Site Investigator, and Coordinating Centre PV Officer, supported by the CI and TMC on behalf of the sponsor. The causality assessment given by the Site Investigator will not be downgraded by the Sponsor.

The investigator/sponsor must make an assessment of whether the adverse event/serious adverse event is likely to be related to study treatment (colchicine or usual care medications) according to the following definitions:

Unrelated: Event is unrelated to study treatment (colchicine or usual care)

Possibly: Although a relationship to study medication (colchicine or usual care) cannot be completely ruled out, the nature of the event, the underlying disease, concomitant medication or temporal relationship make other explanations possible.

Probably: The temporal relationship and absence of a more likely explanation suggest the event could be related to the study medication (colchicine or usual care).

All AEs/SAEs judged as having a reasonable suspected causal relationship (e.g. possibly, probably) to study medication or to an interaction between study medication and another medication will be considered as Adverse Reactions/Serious Adverse Reactions.

Alternative causes such as natural history of the underlying disease, concomitant therapy, other risk factors and the temporal relationship of the event to the treatment should be considered.

11.3.3 Assessment of severity

The Site Investigator will assess and report the severity of each adverse event on the CRF as follows:

Mild: Event is easily tolerated, causes minimal discomfort and does not impair daily activities.

Moderate: Event is sufficiently discomforting to partly interfere with normal daily activities.

Severe: Event prevents normal daily activities.

Note: the term 'severe' should not be confused with 'serious' which is a regulatory definition based on subject/event outcome or action criteria.

Assessment of expectedness

If an event is coded as an adverse reaction, the expectedness of the reaction will be determined by the Coordinating Centre team (acting for the sponsor) according to the reference safety information.

11.3.4 Emergency unblinding procedures

As the study is unblinded for patients and treating physicians, no unblinding procedure applies.

11.4 Reporting procedures for all adverse events

All AEs occurring during the trial, in both Usual Care and Colchicine-treated arms, will be recorded on the Adverse Event Form in the CRF, whether or not attributed to the study medication. In addition, SAEs will be reported within 24 hours to the pharmacovigilance office using the SAE Reporting Form (available in the site file), by emailing sae.reporting@ucd.ie.

The following information will be recorded: subject code ID, description, date of onset and end date, severity, causality to study colchicine (for subjects on Colchicine-treated arm only), seriousness, outcome, and action taken. Follow-up information will be provided as necessary.

Regardless of severity, AEs considered related (at least possibly) to colchicine will be followed until resolution or until the event is considered stable by the Site Investigator. All colchicine-related AEs that result in a subject's withdrawal or are present at the end of the trial, will be followed up to resolution.

Targeted reporting of adverse events:

Certain adverse events are reported more frequently in patients with stroke or treated with colchicine. Adverse events in the following categories should be reported as standard, regardless of study arm:

(a) Bone marrow depression
(b) New peripheral neuropathy
(c) Abdominal pain, nausea, vomiting, diarrhoea
(d) Hepatic injury
(e) Myopathy, myalgias, rhabdomyolysis
(f) New acute or chronic renal injury
(g) Oligospermia or azoospermia
(h) Low serum B12
(i) Infections
(j) New malignancy (cancer)
(k) Major haemorrhage

Definitions for each category are provided in Appendix 14.

Other events may be reported if meeting the definition of adverse events in the opinion of the site investigator.

Stopping colchicine due to Adverse Event:

It will be left to the Site Investigator's clinical judgment (supported by the Trial Coordination Centre) whether or not an AE is of sufficient severity to require the cessation of colchicine treatment. Procedures in this event are described in Section 9.5.1.

11.4 Reporting procedures for serious adverse events

The investigator will report all SAEs immediately to the Pharmacovigilance office (acting for the sponsor) via the Serious Adverse Event form in the site file, by emailing ***sae.reporting@ucd.ie.***. The immediate report will be followed by detailed, written reports. The immediate and follow-up reports will identify subjects by unique code numbers. The immediate report will be made by the investigator within a very short period of time not exceeding **24 hours** following knowledge of the SAE.

Additional information received for a case (follow-up or corrections to the original case) need to be detailed on a new SAE form and sent without delay to the Coordinating Centre team.

All SAEs will be evaluated on receipt by the sponsor, and will be assessed for seriousness, causality and expectedness. SAEs classified as SUSARs will be reported to Research Ethics Committees (REC), National Competent Authorities/Regulatory Agencies and Investigators, according to the applicable regulatory requirements and sponsor standard operating procedures.
SAEs classified as SARs will also be reported to DMC by the sponsor within 15 days of first recognition by the Site Investigator.

Protocol Update: Reporting of cases of COVID-19 as SAEs:

All cases of COVID-19 identified in trial subjects should be reported as Serious Adverse Events, regardless of whether the patient is in the colchicine or usual care arm, and even if the symptoms are mild

11.5 Reporting procedures for Suspect Unexpected Serious Adverse Reactions

The Coordination Centre team (acting for the sponsor) will report fatal or life- threatening SUSARs to the local ethics committees and to the national regulatory authorities according to local regulations, but no later than 7 days of notification by the Site Investigator. If the initial report is incomplete, the Coordination Centre team will submit a completed report based on the initial information within an additional eight days.

SUSARs which are not fatal or life-threatening will be reported to the local ethics committees and to the national regulatory authority according to local regulations, but no later than within 15 days of notification by the Site Investigator.

If significant new information on an already reported case is received by the Coordination Centre, this information will be reported as a follow-up report within 15 days of the receipt of new information.

The Trial Coordination Centre will also inform all investigators concerned of relevant information about SUSARs that could adversely affect the safety of subjects.

11.6 Development Safety Update Reports (DSURs)

As required by law, the Trial Coordination Centre (acting for the sponsor) will submit once a year throughout the clinical trial or on request, a DSUR to the relevant competent authorities and relevant ethics committees (ICH guideline E2F).

11.7 Pregnancy

Pregnancy is not considered an AE or SAE. Although pregnancy is considered highly unlikely during the trial because women of child-bearing potential are excluded, each Site Investigator must collect pregnancy information for female trial subjects or female partners of male trial subjects who become pregnant while participating. The Site Investigator will record the information on a Pregnancy Form in the CRF and submit this to the Coordinating Centre immediately (within 24 hours of awareness). Any pregnancy that occurs will be followed to outcome, and the development of the new born followed for 1 year post delivery, with parental consent, as appropriate.

11.8 Data monitoring committee (DMC)

An independent Data Monitoring Committee will oversee the trial to protect the safety and wellbeing of the trial participants and to ensure the integrity of the trial data. Membership of the DMC will be independent of the investigators recruiting patients into the trial and will report to the Steering Committee. The DMC will include an independent statistician, who will not be involved in the final analysis of the primary or other outcomes and will not be a member of the TSC.

During the period of recruitment into the study, the DMC will conduct at least 2 interim reviews of safety and analyses for overwhelming efficacy of the intervention when 50% and 75% of the subjects have been recruited. These reviews will be distinct and separate from the review of recruitment, safety, tolerability, and pooled outcome rates (ie. not stratified by study treatment arm) which will be performed at the end of the Vanguard stage.

DMC reviews will include clinical assessment of Adverse Events and statistical analysis using flexible group sequential methods (O'Brien-Fleming and Peto statistical boundaries[35]) with a pre-specified alpha-spending function to preserve the overall Type 1 alpha for the primary outcome analysis at 0.05.

In the light of these reviews, the DMC will advise the chairman of the Trial Steering Committee if, in their view, sufficient evidence exists for (i) a clear conclusion to be made that, for all patients (or some), the treatment is clearly indicated or clearly contra-indicated or (ii) evidence that might reasonably be expected to materially influence future patient management.

Following a report from the DMC, the Trial Steering Committee will decide whether to modify or halt entry to the study (or seek extra data). Detailed membership and operating procedures for the DMC will be outlined in the DMC Charter.

11.9 Trial Steering Committee:

The membership and responsibilities of the TSC are defined in the TSC Charter.

11.10 Outcomes Reporting and Blinded Outcome Adjudication:

Suspected outcomes will be reported without delay by Site Investigators to the Coordinating Centre Team via the eCRF. Pre-specified supporting documentation will be anonymised, de-identified, coded, and provided to the Coordinating Centre team, as detailed in Appendix 9. An independent blinded Outcomes Committee will consist of at least 3 physicians with experience in stroke medicine and cardiology, not involved in patient recruitment for the trial. It will report to the Steering Committee and Chief Investigator, and will verify and categorise suspected outcome events, blinded to treatment allocation. It will meet by teleconference/videoconference regularly.

Note: If site team think a patient probably had an outcome, but it is still not fully clear that a definite outcome has occurred, then please email the trial project office within 24 hours (isctn@ucd.ie). The team will help with monitoring this patient until it is clear that an outcome has occurred, or else the event may need to be re-classified and reported without delay as an SAE.

11.11 Periodic reporting of safety (Switzerland only)

An annual safety report shall be submitted once a year to the relevant Ethics Committee and to SwissMedic. If security or safety measurements must be taken directly while a clinical trial is being carried out, the examiner must inform the ethical review committee which measurements were taken and the circumstances under which they were necessary, within 7 days (Clinical trials article 37).

12 STATISTICS

12.1 Overall analysis strategy

The analysis will be performed by Prof Leslie Daly and Prof Cathal Walsh, both experienced medical statisticians. The specific primary objective of the study is to compare low-dose colchicine (0.5mg/day) plus usual care with usual care alone for the prevention of the composite outcome of non-fatal recurrent ischaemic stroke, non-fatal myocardial infarction or cardiac arrest, unstable angina requiring hospitalisation, or vascular death, compared on an intention-to-treat basis.

12.2 Determination of sample size

The original sample size for CONVINCE was 2,623 patients. However, data from recent clinical trials and registries indicate that the rate of stroke/cardiac recurrent events may have reduced since the initial sample-size calculation. In FOURIER, the annualized major cardiovascular event (MACE) rate in statin-treated stroke patients was 3.8%, in PROFESS it was 5.2%, and the 1-year MACE rates in NAVIGATE-ESUS were 5.4% and in TIARegistry.org was 6.2%. The original estimate of the annualized MACE rate in the control arm of CONVINCE was 6%/year, or 18% over a median 3-year follow-up period. Based on these more recent data, as a precautionary measure to maximise statistical power, a protocol amendment has been made to increase the trial sample size based on a more conservative estimate annualized rate of our composite outcome in controls of 4.5%/year (13.5% over median 3-year follow-up).

The primary analysis uses the Intention to Treat (ITT) principle analysing all subjects including a drop-out rate of 15% in the colchicine arm. Assuming a 25% effect size in colchicine-treated patients after adjusting for a 15% non-compliance rate (relative hazard 0.75, alpha 0.05, power 80%), the revised sample size is increased to 3,154 patients (see Appendix 13). This may over-estimate the required sample size, as the annualized estimated outcome rates are deliberately conservative, and some trials have reported higher annualized rates (above). The estimated effect size of 25% is also deliberately conservative - in the Australian LoDoCo trial, the effect size associated with colchicine was 67% (hazard ratio 0.33, 95% CI 0.18–0.59). However, the sample size is deliberately based on conservative estimates to reduce the risk of inadequate power.

No patients were lost to follow up in the Australian LoDoCo trial and the rate of loss to follow up is minimal (<1%) in our earlier studies. Our follow-up schedule is rigorous and frequent, and each patient will contribute information for the duration of their involvement in our time-to-event analysis. Therefore, the sample size is not inflated further to deal with lost follow-up.

12.3 Analysis sets

Analysis of the primary outcome measure will be performed on an intention-to-treat (ITT) basis, including all patients randomised to colchicine or usual care arms.

In a secondary, pre-specified ‘on-treatment’ analysis, the primary composite outcome will also be analysed in patients who adhere to study medication for 30 days or more.

12.4 Demographic and baseline disease characteristics

Demographic and baseline disease characteristic data will be summarized for each treatment group by presenting frequency distributions and/or descriptive statistics.

12.5 Analysis of primary outcome

On trial completion, the analysis of primary outcome (defined in Section 8.1) will be by a strict intention-to-treat analysis, including all randomised subjects, regardless of patient withdrawals or non-adherence in colchicine or usual care arms. The statistical

approach will be a comparison of time to primary outcome event in colchicine-treated and usual-care groups using the log-rank test. A p-value of 0.05 or less will be considered as statistically significant.

12.6 Analysis of secondary outcomes - safety

Appropriate correction for multiple statistical comparisons for interim analyses will be done.

Each specific safety outcome defined in section 8.2.1 will be compared using the chi-squared or Fisher exact test (the exact test will be used for counts in either arm of less than 5 events). In addition, all Serious Adverse Events will be compared using chi-squared or Fisher exact test.

12.7 Analysis of secondary outcomes - efficacy

Each secondary efficacy outcome defined in section 8 will be compared in colchicine-treated and usual care groups as follows:

(1) On-treatment comparison of time to the primary outcome in colchicine and usual care groups, using the log-rank test. 'On-treatment' will be defined as any patient who received allocated intervention and remained compliant for 30 days or more.

(2) Individual comparisons of time to each component of the primary outcome (ischaemic stroke, non-fatal MI, non-fatal cardiac arrest, and vascular death) will be performed, using the log-rank test

(3) Comparison of time to fatal and non-fatal ischaemic stroke combined, using the log-rank test

(4) Comparison of times to recurrent disabling ischaemic stroke (modified Rankin score 3-5) and to recurrent non-disabling ischaemic stroke (modified Rankin score 0-2) between colchicine and usual care arms will be performed.

(5) Comparison of disability (modified Rankin score) by ordinal logistic regression (shift analysis) and proportions in each arm with no or mild disability (Rankin score 0-2) by chi-squared test.

(6) Subgroup analyses for interaction will be sought between colchicine treatment and important subgroup variables which may influence the primary outcome. These will include age (categorised), gender, qualifying event (stroke or TIA), time since qualifying stroke/TIA, stroke subtype (TOAST-defined large artery, small artery, undetermined), baseline lipid status, diabetes, hypertension, smoking status, atrial fibrillation/no atrial fibrillation (identified during the trial, as atrial fibrillation at screening is an exclusion criterion).

(7) Health economic analysis: Within-trial economic analysis of direct resource costs and health outcomes on an intention to treat basis will be performed. We will estimate three year cumulative costs of inpatient episodes, hospital outpatient visits, care home stays and home case visits, using manual linkage to routine data and valuing length of hospital stay distributions (LOS) using unit (weighted average bed-day) costs derived from country-specific validated sources. Outpatient visits,

care home stays and home case visits will be valued using the estimates reported by Brick[28,29]. Nominal costs will be reported in real terms using pay and prices inflator.

Survival times will be adjusted using the EQ5D-5L (Appendix 5) to calculate quality[30]

adjusted life years (QALYs)), with multiple imputation for missing values.

The primary treatment effect will be estimated using an individual level regression model for mean incremental costs and incremental QALYs. Longer run modelling will estimate the distribution of costs and QALYs calculated over the expected patient lifetimes. A microsimulation model will be calibrated using information gained from the within trial analysis of cost-effectiveness combined with additional cost/survival/quality-of life data from published data and expert beliefs on the distributions of parameters where information is less-readily available. The structural uncertainty in the long run model will be addressed using model averaging methods[31,32].

The economic analysis will contribute to a structured overview of colchicine cardiovascular treatment effects taking advantage of information from systematic reviews and individual trials. Generalisability and assessment of treatment effect heterogeneity will be enhanced by the pooled data across these trials.

12.8 Pre-specified exploratory analyses:

(1) Effect of colchicine on change in cognition and development of dementia between baseline and end of study, measured by the Montreal Cognitive Assessment (MOCA, Appendix 3)

(2) Effect of colchicine on Health-related quality of life, measured by EuroQoL (EQ5D-5L, Appendix 5)

(3) Association of baseline CRP and primary outcome

(4) Relationship of colchicine and primary outcome, according to CRP measured at baseline

(5) Cumulative events: The relationship between colchicine therapy and the cumulative total number of component events in the primary outcome cluster detected over the duration of the trial will be investigated.

12.9 The level of statistical significance

A p-value of 0.05 or less will be considered as statistically-significant (two-sided alpha).

12.10 Procedure for accounting for missing, unused and spurious data

The primary outcome for this trial is time to the first occurrence of non-fatal recurrent ischaemic stroke, non-fatal myocardial infarction, non-fatal cardiac arrest, or vascular death. If a patient is lost to follow-up or withdrawn without further follow-up before trial completion this endpoint will be missing. The intention to treat principle requires that missing outcome data cannot be ignored because information from all randomized patients should be included[33]. Missingness may be related to randomization group so drop-out rates will be examined carefully. We will use the inverse Kaplan-Meier approach to achieve this[34]

For the primary analysis, which will use Kaplan-Meier survival curves and the logrank test for statistical significance, such missing data will be considered as censored at the date last known to be alive. This makes the Missing at Random (MAR) assumption for such data. Sensitivity analyses will be undertaken to evaluate the effect of the missingness in the data. Firstly, in the worst-case scenario, all lost to follow-up patients will be considered to have had an event at the time last known to be alive.

Secondly the worst-comparison scenario will be examined with losses in the treatment group taken as having had an event while losses in the control group being censored. A similar approach will be taken for other time to event variables. This imputation method is not biased towards a positive treatment effect.
For continuous outcome variables which have been measured on multiple occasions the primary analysis will be by a Mixed Model Repeated Measures approach. This technique essentially imputes the final value using the actual values measured when the final outcome is missing. For a sensitivity analysis a multiple imputation approach to missing values will be taken. Five imputations will be performed sampling from a posterior Baysian distribution of the baseline values of the variable in question. Sampling from the baseline distribution values does not assign any treatment benefit in the imputed values.

Multiple imputation will also be used for key non-outcome variables. We anticipate that it may be important to adjust for potential confounders if drop-out rates differ between the treatment groups, and a strategy to deal with missing values for potential confounding variables will be based on multiple imputation. Using a regression approach the non-missing confounders in the model will be used to impute the missing confounder with a multivariate random component added in. Five imputations will be carried out. A sensitivity analysis will be undertaken looking at the effects of other and no imputation methods on the trial outcome. Data will not be imputed where more than 15% of cases are missing for a given variable.

12.11 Procedure for reporting any deviation(s) from the original statistical plan

Procedures for assessing and reporting any deviation(s) from the original statistical plan will be:

1. Review of justification and revised approach to the original statistical plan by the Steering Committee and DMC
2. Approval or non-approval of revised approach, by Steering Committee and DMC
3. Reporting of revised approach in writing to relevant Ethics Committees and Competent Authorities for the participating member state.
4. Incorporation of revised approach into protocol as a Protocol Amendment
5. Informing of Protocol Amendment to site investigators

12.12 Timing of analyses

The primary and secondary efficacy and safety comparisons will be performed at study completion. No hypothesis testing will be performed during monitoring of primary outcome measures at interim meetings of the Steering Group or Data Monitoring Committee. The overall study duration is anticipated to be 5 years and 3 months, including the primary and secondary analyses outlined above. Analyses will be conducted in the final 3 months.

13 DIRECT ACCESS TO SOURCE DATA/DOCUMENTS

Direct access will be granted to authorised representatives from the sponsor, host institution and the regulatory authorities to permit trial-related monitoring, audits and inspections.

14 DATA HANDLING AND RECORD KEEPING

14.1 Data collection, source documents and case report forms (CRF)

Source documents for this study will include hospital records and procedure reports and data collection forms. These documents will be used to enter data on the CRFs. An electronic CRF will be used for this study. However, data may be entered initially on paper CRFs, and subsequently entered digitally at each site on electronic CRFs. All data entered on paper CRFs must be entered legibly. If an error is made, the error will be crossed through with a single line in such a way that the original entry can still be read. The correct entry will then be clearly inserted, and the alterations will be initialled and dated by the investigator.

Data reported on the CRF that are derived from source documents must be consistent with the source documents or the discrepancies must be explained.

All documents will be stored safely in confidential conditions. On all study-specific documents other than the signed consent, the subject will be referred to by the study subject identification number/code.

14.2 Data management and reporting

Central data management will be performed by the Data Management Centre at the Health Research Board Clinical Research Facility at National University of Ireland, Galway. Local user access to the electronic CRF will be controlled via assigned usernames and passwords, approved by the study Data Manager based at NUIG. Access to the central study database will be governed by CRFG Standard Operating Procedures and signed off by the Lead Site Investigator. Audit trails will log all transactions of data into and out of the system including time, date, user ID and the records involved. All external electronic communication with the central database will be protected by using Secure Socket Layer technology. The main database will be hosted in a secure enterprise scale data centre.

The Research team will take every precaution to respect privacy in accordance with relevant legislation and EU Directive 95/46/EC on protection of individuals with regard to the processing of personal data. No data will be transferred outside the EU. During data processing, the identity of the donor will be protected.

The data in the study database will be pseudo-anonymised, so that a number will be assigned to each patient which will be mapped to identifiable patient details at each hospital site only. This means that the data in the database is non-identifiable but will permit re-identification by the local site investigator in case of emergencies and requirement to follow up the patient.

15 RETENTION OF ESSENTIAL DOCUMENTS

All records and documents will be maintained by the investigator for a period of at least 5 years after end of trial. In Canada only records will be retained and maintained for 25 years, as per section C.05.012 of Canadian Food and Drug Regulations. In Belgium records also need to be retained for 25 years after the end of the study. In Switzerland, in accordance with Article 45 ClinO, data will be retained for 10 years after the conclusion or revocation of the clinical experiment.

In the event of withdrawal, the data collected up to that point will still be evaluated in encrypted form, otherwise the entire project will lose its value. After the evaluation, the data will be

completely anonymized, i.e. the key assignment is destroyed, so that no one can know who originally provided the data.

16 QUALITY CONTROL AND QUALITY ASSURANCE PROCEDURES

All sites will have remote data monitoring conducted by the Trial Coordination Centre team. Data will be checked for completeness, logic, and validity. Queries will be sent to sites to verify data as required.

For on-site monitoring trained study staff will visit sites at agreed intervals and will review all informed consent forms and a sample of source documents and CRFs from enrolled subjects. A standard operating procedure for site monitoring visits will be implemented.

This study will be conducted in accordance with the current approved protocol, ICH GCP, relevant regulations, and standard operating procedures.

17 AUDITS AND INSPECTIONS

This trial may be subject to internal or external auditing or inspections procedure to ensure adherence to GCP. Access to all trial-related documents will be given at that time.

The Sponsor and any Participating Site Investigators should understand that source documents for this trial should be made available to appropriately qualified personnel from the Sponsor or designee or to health authority inspectors after appropriate notification.

18 ETHICS

The trial will have a rigorous oversight structure to ensure patient safety, including standardized procedures for site monitoring, adverse effect monitoring and reporting, and oversight provided by the Trial Steering Committee and Data Safety and Monitoring Committee. The DSM Committee will be independent of the Trial Steering Committee and will include a patient representative. Its primary role will be to ensure the protection of study subjects during the course of the trial.

18.1 Declaration of Helsinki

The trial will be conducted in accordance with the ethical principles that have their origins in the Declaration of Helsinki. The trial will be conducted within a strict regulatory and ethical framework, according to the Declaration of Helsinki, and relevant governing legislation in participating countries. Regulatory approvals will be obtained from medicines regulatory authorities in the participating countries.

18.2 Good Clinical Practice

This study will be conducted in accordance with Good Clinical Practice (GCP), as defined by the International Conference on Harmonisation (ICH) and in accordance with the ethical principles underlying governing legislation in participating countries.

18.3 Approvals

The trial protocol will be reviewed and approved by a Recognised Ethics Committees with responsibility for each participating hospital before patients are approached for entry. Required documents including the protocol, informed consent form, subject

information leaflet, investigational medicinal product dossier/SPC, investigators brochure and any other required documents will be submitted to a recognised research ethics committee and the competent authority for written approval.

The sponsor will submit and obtain approval from the above parties for substantial amendments to the original approved documents.

Switzerland only: In accordance with Art 38 ClinO, the completion of the study will be reported by the local site investigator to the relevant ethics committee within a period of 90 days. Premature discontinuation or interruption of the study will be reported by the local site investigator to the relevant ethics committee within 15 days and will include an explanation of the circumstances. In addition, a final report will be submitted to the Ethics Committee within one year of completion or discontinuation of the study. Reporting duties and timelines are the same as for the relevant Ethics Committee, except of non-substantial amendments that shall be reported as soon as possible. These obligations rest on the sponsor.

18.4 Informed consent

The overall aim of the trial, trial procedures, possible risks, benefits, and alternatives to treatment will be explained by study personnel to each patient before recruitment, and each will be given written information detailing the trial. Patients will be encouraged to discuss the trial with family members and their treating physician before entering. Each patient will provide written informed consent prior to any screening assessments required by the study being performed.

18.5 Benefits and risks assessment

The risks of low-dose colchicine in the intended sample group for study are judged to be low, as outlined in previous sections and the SPC. The benefits may be as high as a 66% reduction in the risk of recurrent stroke, coronary events, or vascular death, as reported in the LoDoCo trial. The anticipated benefit risk to the patient population will be evaluated on an ongoing basis by the sponsor.

18.6 Subject confidentiality

Patient data will be subject to rigorous procedures to ensure data protection, as outlined in detail in the data protection section of this application. Patient information will be pseudo-anonymised, and no patient will be identified in any public presentation of material related to the trial.

The sponsor will take every precaution to respect privacy in accordance with relevant legislation in participating countries governing protection of individuals with regard to the processing of personal data. All study documents will be stored securely. During data processing, the identity of the donor will be protected.

The data in the study database will be pseudo-anonymised, so that a number will be assigned to each patient which will be mapped to identifiable patient details at each hospital site only. This means that the data in the database is non-identifiable but will permit re-identification by the local site investigator in case of emergencies and requirement to follow up the patient. In accordance with patient’s rights, only data relevant for the project will be processed.

The participation in the study and the identity of the subjects will be treated as confidential and no patient identifiable records or results relating to the study will be disclosed to any third party other than the authorized investigators and/or regulatory

bodies for medicines, for the purposes of effecting registration of the trial or for the purposes of medical research or as may otherwise be required by law. The recorded data will be passed on to authorized persons in an anonymous form. If the results of the study are published, the patient's identity will remain confidential.

19 FINANCING AND INSURANCE/INDEMNITY

The budget will be held at the Research Finance Department of the sponsor, University College Dublin. Funding is provided by the Health Research Board of Ireland. Contracts will be prepared, and contractual issues will be managed by the Legal Office in UCD under the supervision of Ms Caroline Brennan, University Solicitor.

Trial insurance will be obtained by UCD, acting as sponsor for the trial. Public Liability (‘negligent harm’) and Clinical Trial (‘non-negligent harm’) insurance policies which apply to this trial will be in place.

20 CLINICAL STUDY REPORT

The study report will be signed by the Principal Investigator and Writing Subgroup of the Steering Committee.

21 REFERENCES

1. WHO. Preventing chronic diseases: a vital investment. Geneva Switzerland: World Health Organisation ,2005.http://www.who.int/chp/chronic_disease_report/en/index.html(Accessed

2. American Heart Association. Heart and Stroke Statistics 2013 update. Circulation 2013;127;e6-e245

3. Smith, S, Horgan, F, Sexton E, Cowman S, Hickey A, Kelly P, McGee H, Murphy S, O'Neill D, Royston M, Shelley E, Wiley M. Cost of Stroke in Ireland: Estimating the Annual Economic Cost of Stroke and TIA in Ireland. Irish Heart Foundation, 2010.

4. Kelly PJ, Crispino G, Sheehan O, Kelly L, Marnane M, Merwick A, et al. Incidence, event rates, and early outcome of stroke in Dublin, Ireland: the North Dublin population stroke study. Stroke 2012;43:2042–7

5. Marnane M, Duggan CA, Sheehan OC, Merwick A, Hannon N, Curtin D, Harris D, Williams EB, Horgan G, Kyne L, McCormack, Duggan J, Moore A, Crispino-O'Connell G, Kelly PJ. Stroke subtype classification to mechanism-specific and undetermined categories by TOAST, A-S-C-O and Causative Classification System - Direct comparison in the North Dublin Population Stroke Study. Stroke 2010;41:1579-1586

6. Wardlaw JM, Smith C, Dichgans M. Mechanisms of sporadic cerebral small vessel disease: insights from neuroimaging. Lancet Neurol 2013;32:483-97

7. Sheehan OC, Kyne L, Kelly LA, et al. A population-based study of ABCD2 score, carotid stenosis, and atrial fibrillation for early stroke prediction after TIA: the North Dublin TIA Study. Stroke 2010; 41: 844-845

8. Mohan KM, Wolfe CDA, Rudd AG, Heuschmann PU, Kolominsky-Rabas PK. Grieve AP. Risk and cumulative risk of stroke recurrence: A systematic review and meta-analysis. Stroke 2011;42:1489-1494

9. Touzé E, Varenne O, Chatellier G, Peyrard S, Rothwell PM, Mas JL. Risk of myocardial infarction and vascular death after transient ischaemic attack and ischaemic stroke. A systematic review and meta-analysis. Stroke 2005;36:2748-55

10. Ridker PM, Luscher TF. Anti-inflammatory therapies for cardiovascular disease. Eur Heart J 2014;35:1782- 1791

11. Libby P. Inflammation in atherosclerosis. Nature 2002;420:868-874

12. Tawakol A, Migrino RQ, Bashian GG, et al. In vivo 18F-fluorodeoxyglucose positron emission tomography imaging provides a noninvasive measure of carotid plaque inflammation in patients. J Am CollCardiol 2006;48:1818-1824

13. Marnane M, Merwick A, Sheehan OC, Hannon N, Foran P, Grant T, et al. Carotid plaque inflammation on (18) F-fluorodeoxyglucose positron emission tomography predicts early stroke recurrence. Ann Neurol 2012;71:709-718

14. Rosenberg GA. Inflammation and white matter damage in vascular cognitive impairment. Stroke 2009;40[suppl 1];S20-S23

15. Esiri MM, Wilcock GK, Morris JH. Neuropathological assessment of the lesions of significance in vascular dementia J NeuroNeurosurg Psychiatry 1997;63;749-753

16. Amarenco P, Benavente O, Goldstein LB, Callahan A, Sillesen H, Hennerici MG, et al. Results of the Stroke Preventiion by Aggressive Reduction in Cholesterol Levels (SPARCL) Trial by stroke subtypes. Stroke 2009;40:1405-1409

17. Elkind M, Luna JM, McClure LA, Zhang Y, Coffey CS, Roldan A, et al. C-Reactive Protein as a Prognostic Marker After Lacunar Stroke; Levels of Inflammatory Markers in the Treatment of Stroke Study Stroke. 2014;45:707-16

18. Tahara N, Kai H, Ishibashi M, et al. Simvastatin attenuates plaque inflammation: evaluation by fluorodeoxyglucose positron emission tomography. J Am Coll Cardiol 2006;48:1825-1831

19. Tawakol A, Fayad Z, Mogg R, et al. Intensification of Statin Therapy Results in a Rapid Reduction in Atherosclerotic Inflammation; Results of a Multicenter Fluorodeoxyglucose-Positron EmissionTomography/Computed Tomography Feasibility Study. J Am Coll Cardiol 2013;62:909–17

20. Duewell P, Kono H, Rayner KJ, Sirois CM, Vlademir G, Bauernfeind FG, et al. NLRP3 inflammasomes are required for atherogenesis and activated by cholesterol crystals. Nature. 2010;464:1357-1362

21. Rajamäki K, Lappalainen J, Oorni K, Välimäki E, Matikainen S, Kovanen PT, et al. Cholesterol Crystals Activate the NLRP3 Inflammasome in Human Macrophages: A Novel Link between Cholesterol Metabolism and Inflammation. PLoS ONE. 5(7):e11765

22. Abela GS. Cholesterol crystals piercing the arterial plaque and intima trigger local and systemic inflammation. J Clin Lipid 2010;4:156-164

23. Stack J, Ryan J, McCarthy G. Colchicine: New insights to an old drug. Am J Therapeutics 2013; PMID 24100258

24. Crittenden DB, Lehmann RA, Schneck L, et al. Colchicine use is associated with decreased prevalence of myocardial infarction in patients with gout. J Rheumatol 2012;39:1458-64

25. Nidorf M, Thompson PL. Effect of colchicine (0.5mg twice daily) on high-sensitivity C-reactive protein independent of aspirin and atorvastatin in patients with stable coronary artery disease. Am J Cardiol 2007;99:805-807

26. Nidorf SM, Eikelboom JW, Budgeon CA, Thompson PL. Low-dose colchicine for secondary prevention of cardiovascular disease. J Am CollCardiol 2013;61:404-410

27. Imazio M, Bobbio M, Cecchi E, et al. Colchcine as first-choice therapy for recurrent pericarditis. Results of the CORE (colchicine for recurrent pericarditis) trial. Arch Int Med 2005;165:1987-1991

28. Brick, A., S. Smith, C. Normand, S. O'Hara and E. Tyrell, forthcoming. "Economic Evaluation of Palliative Care in Ireland". Dublin, ESRI/TCD.

29. Curtis, L (ed.) Unit Costs of Health and Social Care 2013, PSSRU, University of Kent

30. Janssen MF, Pickard AS, Golicki D, Gudex C, Niewada M, Scalone L, Swinburn P, Busschbach J. Measurement properties of the EQ-5D-5L compared to the EQ-5D-3L across eight patient groups: a multi- country study. Qual Life Res. 2013 Sep;22(7):1717-275.

31. Baio, G.. Bayesian Methods in Health Economics. 2012, Boca Raton, FL Chapman Hall, CRC

32. Jackson CH, Thompson SG, Sharples LD. Accounting for uncertainty in health economic decision models by using model averaging. J R Stat SocSer A Stat Soc. 2009 Apr;172(2):383-404

33. Committee for Medicinal Products for Human Use (CHMP), Guideline on Missing Data in Confirmatory Clinical Trials, EMA 2011

34. M Schemper and TL Smith. A note on quantifying follow-up in studies of failure time. Controlled clinical trials. 1996;17:343-346

35. Peto R, Pike M, Armitage P, Breslow N, Cox D, Howard S. Design and analysis of randomised clinical trials requiring prolonged observation of each patient. II. analysis and examples. British Journal of Cancer 1977;35:1-39

APPENDIX 1: MODIFIED RANKIN SCORE, STANDARDISED ALGORITHM

MODIFIED RANKIN SCALE (MRS)

Patient Name: ____________________

Rater Name: ____________________

Date:

Score	Description
0	No symptoms at all
1	No significant disability despite symptoms; able to carry out all usual duties and activities
2	Slight disability; unable to carry out all previous activities, but able to look after own affairs without assistance
3	Moderate disability; requiring some help, but able to walk without assistance
4	Moderately severe disability; unable to walk without assistance and unable to attend to own bodily needs without assistance
5	Severe disability; bedridden, incontinent and requiring constant nursing care and attention
6	Dead

TOTAL (0–6): _____

References

Rankin J. "Cerebral vascular accidents in patients over the age of 60."
Scott Med J 1957;2:200-15

Bonita R, Beaglehole R. "Modification of Rankin Scale: Recovery of motor function after stroke."
***Stroke* 1988 Dec;19(12):1497-1500**

Van Swieten JC, Koudstaal PJ, Visser MC, Schouten HJ, van Gijn J. "Interobserver agreement for the assessment of handicap in stroke patients."
***Stroke* 1988;19(5):604-7**

Provided by the Internet Stroke Center — www.strokecenter.org

STANDARDISED mRS ALGORITHM .

Figure. The simplified mRS questionnaire algorithm.

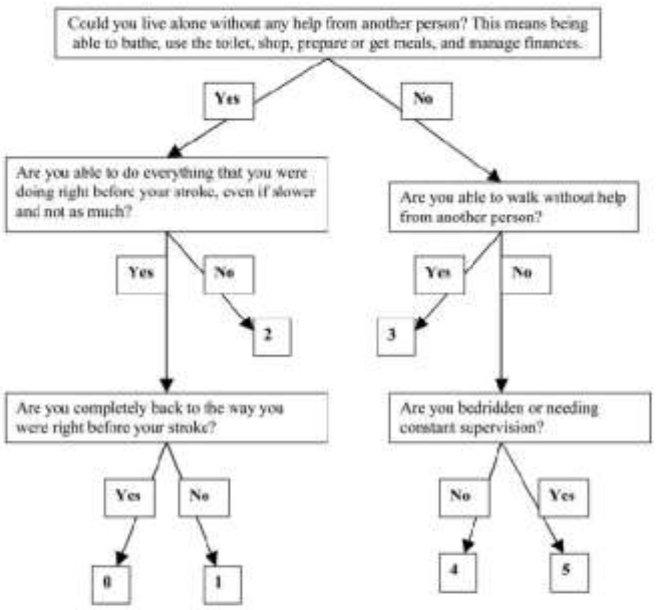

Askiel Bruno et al. Stroke. 2010;41:1048-1050

APPENDIX 2: ABCD2 SCORE

ABCD2 score:

Risk Factors	Score
Age	
<60 Years	0
≥60 years	1
Blood Pressure	
Systolic 140 mmHg or diastolic 90 mmHg or greater	1
Systolic <140 mmHg and diastolic <90 mmHg	0
Clinical Features	
Unilateral weakness	2
Isolated speech disturbance (dysarthria, dysphasia)	1
Neither weakness nor speech disturbance	0
Duration of Symptoms	
<10 minutes	0
10-59 minutes	1
≥60 minutes	2
Diabetes	
No	0
Yes	1
Total Score (0-7)	

References

Johnston SC, et al. Validation and refinement of scores to predict very early stroke risk after transient ischaemic attack. Lancet. 2007;369(9558):283-92

APPENDIX 3: MONTREAL COGNITIVE ASSESSMENT (MOCA)

Administration and Scoring Instructions

The Montreal Cognitive Assessment (MoCA) was designed as a rapid screening instrument for mild cognitive dysfunction. It assesses different cognitive domains: attention and concentration, executive functions, memory, language, visuoconstructional skills, conceptual thinking, calculations, and orientation. Time to administer the MoCA is approximately 10 minutes. The total possible score is 30 points; a score of 26 or above is considered normal.

1. **Alternating Trail Making**:

Administration: The examiner instructs the subject: *"Please draw a line, going from a number to a letter in ascending order. Begin here* [point to (1)] *and draw a line from 1 then to A then to 2 and so on. End here* [point to (E)]."

Scoring: Allocate one point if the subject successfully draws the following pattern: 1 –A- 2- B- 3- C- 4- D- 5- E, without drawing any lines that cross. Any error that is not immediately self-corrected earns a score of 0.

2. **Visuoconstructional Skills (Cube)**:

Administration: The examiner gives the following instructions, pointing to the **cube**: *"Copy this drawing as accurately as you can, in the space below"*.

Scoring: One point is allocated for a correctly executed drawing.
• Drawing must be three-dimensional
• All lines are drawn
• No line is added
• Lines are relatively parallel and their length is similar (rectangular prisms are accepted)

A point is not assigned if any of the above-criteria are not met.

3. **Visuoconstructional Skills (Clock)**:

Administration: Indicate the right third of the space and give the following instructions: *"Draw a* ***clock****. Put in all the numbers and set the time to 10 past 11"*.

Scoring: One point is allocated for each of the following three criteria:
• Contour (1 pt.): the clock face must be a circle with only minor distortion acceptable (e.g., slight imperfection on closing the circle);
• Numbers (1 pt.): all clock numbers must be present with no additional numbers; numbers must be in the correct order and placed in the approximate quadrants on the clock face; Roman numerals are acceptable; numbers can be placed outside the circle contour;
• Hands (1 pt.): there must be two hands jointly indicating the correct time; the hour hand must be clearly shorter than the minute hand; hands must be centred within the clock face with their junction close to the clock centre.

A point is not assigned for a given element if any of the above-criteria are not met.

4. **Naming**:

Administration: Beginning on the left, point to each figure and say: *"Tell me the name of this*

animal".

Scoring: One point each is given for the following responses: (1) lion (2) rhinoceros or rhino (3) camel or dromedary.

5. **Memory**:

Administration: The examiner reads a list of 5 words at a rate of one per second, giving the following instructions: *"This is a memory test. I am going to read a list of words that you will have to remember now and later on. Listen carefully. When I am through, tell me as many words as you can remember. It doesn't matter in what order you say them".* Mark a check in the allocated space for each word the subject produces on this first trial. When the subject indicates that (s)he has finished (has recalled all words), or can recall no more words, read the list a second time with the following instructions: *"I am going to read the same list for a second time. Try to remember and tell me as many words as you can, including words you said the first time."* Put a check in the allocated space for each word the subject recalls after the second trial.
At the end of the second trial, inform the subject that (s)he will be asked to recall these words again by saying, *"I will ask you to recall those words again at the end of the test."*

Scoring: No points are given for Trials One and Two.

6. **Attention**:

Forward Digit Span: Administration: Give the following instruction: *"I am going to say some numbers and when I am through, repeat them to me exactly as I said them".* Read the five number sequence at a rate of one digit per second.

Backward Digit Span: Administration: Give the following instruction: *"Now I am going to say some more numbers, but when I am through you must repeat them to me in the backwards order."* Read the three number sequence at a rate of one digit per second.

Scoring: Allocate one point for each sequence correctly repeated, (*N.B.*: the correct response for the backwards trial is 2-4-7).

Vigilance: Administration: The examiner reads the list of letters at a rate of one per second, after giving the following instruction: *"I am going to read a sequence of letters. Every time I say the letter A, tap your hand once. If I say a different letter, do not tap your hand".*

Scoring: Give one point if there is zero to one errors (an error is a tap on a wrong letter or a failure to tap on letter A).

Serial 7s: Administration: The examiner gives the following instruction: *"Now, I will ask you to count by subtracting seven from 100, and then, keep subtracting seven from your answer until I tell you to stop."* Give this instruction twice if necessary.

Scoring: This item is scored out of 3 points. Give no (0) points for no correct subtractions, 1 point for one correction subtraction, 2 points for two-to-three correct subtractions, and 3 points if the participant successfully makes four or five correct subtractions. Count each correct subtraction of 7 beginning at 100. Each subtraction is evaluated independently; that is, if the participant responds with an incorrect number but continues to correctly subtract 7 from it, give a point for each correct subtraction. For example, a participant may respond "92 – 85 – 78 – 71

– 64" where the "92" is incorrect, but all subsequent numbers are subtracted correctly. This is one error and the item would be given a score of 3.

7. **Sentence repetition:**

Administration: The examiner gives the following instructions: *"I am going to read you a sentence. Repeat it after me, exactly as I say it* [pause]: ***I only know that John is the one to help today.***" Following the response, say: *"Now I am going to read you another sentence. Repeat it after me, exactly as I say it* [pause]: ***The cat always hid under the couch when dogs were in the room.***"

Scoring: Allocate 1 point for each sentence correctly repeated. Repetition must be exact. Be alert for errors that are omissions (e.g., omitting "only", "always") and substitutions/additions (e.g., "John is the one who helped today;" substituting "hides" for "hid", altering plurals, etc.).

8. **Verbal fluency:**

Administration: The examiner gives the following instruction: *"Tell me as many words as you can think of that begin with a certain letter of the alphabet that I will tell you in a moment. You can say any kind of word you want, except for proper nouns (like Bob or Boston), numbers, or words that begin with the same sound but have a different suffix, for example, love, lover, loving. I will tell you to stop after one minute. Are you ready?* [Pause] *Now, tell me as many words as you can think of that begin with the letter F.* [time for 60 sec]. *Stop."*

Scoring: Allocate one point if the subject generates 11 words or more in 60 sec. Record the subject's response in the bottom or side margins.

9. **Abstraction:**

Administration: The examiner asks the subject to explain what each pair of words has in common, starting with the example: *"Tell me how an orange and a banana are alike"*. If the subject answers in a concrete manner, then say only one additional time: *"Tell me another way in which those items are alike"*. If the subject does not give the appropriate response *(fruit)*, say, *"Yes, and they are also both fruit."* Do not give any additional instructions or clarification. After the practice trial, say: *"Now, tell me how a train and a bicycle are alike"*. Following the response, administer the second trial, saying: *"Now tell me how a ruler and a watch are alike"*. Do not give any additional instructions or prompts.

Scoring: Only the last two item pairs are scored. Give 1 point to each item pair correctly answered. The following responses are acceptable:
Train-bicycle = means of transportation, means of travelling, you take trips in both; Ruler-watch = measuring instruments, used to measure.
The following responses are **not** acceptable: Train-bicycle = they have wheels; Ruler-watch = they have numbers.

10. Delayed recall:

Administration: The examiner gives the following instruction: *"I read some words to you earlier, which I asked you to remember. Tell me as many of those words as you can remember."* Make a check mark (√) for each of the words correctly recalled spontaneously without any cues, in the allocated space.

Scoring: **Allocate 1 point for each word recalled freely without any cues.**

Optional:
Following the delayed free recall trial, prompt the subject with the semantic category cue provided below for any word not recalled. Make a check mark (√) in the allocated space if the subject remembered the word with the help of a category or multiple-choice cue. Prompt all non-recalled words in this manner. If the subject does not recall the word after the category cue, give him/her a multiple choice trial, using the following example instruction, *"Which of the following words do you think it was, NOSE, FACE, or HAND?"*
Use the following category and/or multiple-choice cues for each word, when appropriate:

FACE:	category cue: part of the body	multiple choice: nose, face, hand
VELVET:	category cue: type of fabric	multiple choice: denim, cotton, velvet
CHURCH:	category cue: type of building	multiple choice: church, school, hospital
DAISY:	category cue: type of flower	multiple choice: rose, daisy, tulip
RED:	category cue: a colour	multiple choice: red, blue, green

Scoring: **No points are allocated for words recalled with a cue.** A cue is used for clinical information purposes only and can give the test interpreter additional information about the type of memory disorder. For memory deficits due to retrieval failures, performance can be improved with a cue. For memory deficits due to encoding failures, performance does not improve with a cue.

11. Orientation:

Administration: The examiner gives the following instructions: "Tell me the date today". If the subject does not give a complete answer, then prompt accordingly by saying: *"Tell me the [year, month, exact date, and day of the week]."* Then say: *"Now, tell me the name of this place, and which city it is in."*

Scoring: Give one point for each item correctly answered. The subject must tell the exact date and the exact place (name of hospital, clinic, office). No points are allocated if subject makes an error of one day for the day and date.

TOTAL SCORE: Sum all subscores listed on the right-hand side. Add one point for an individual who has 12 years or fewer of formal education, for a possible maximum of 30 points. A final total score of 26 and above is considered normal.

Appendix 17: MOCA v7.1

MONTREAL COGNITIVE ASSESSMENT (MOCA)

Version 7.1 Original Version

NAME :
Education : Date of birth :
Sex : DATE :

VISUOSPATIAL / EXECUTIVE — POINTS

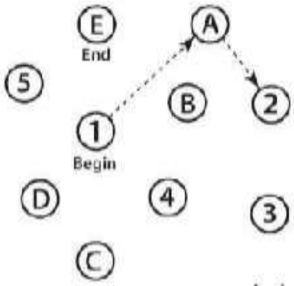

[ ]

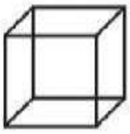

Copy cube [ ]

Draw CLOCK (Ten past eleven) (3 points)

[ ] Contour [ ] Numbers [ ] Hands — __/5

NAMING

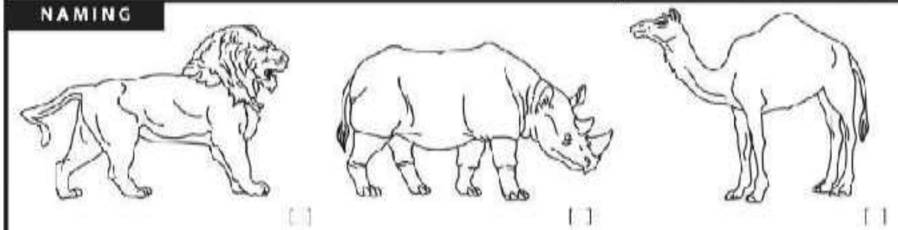

[ ] [ ] [ ] — /3

MEMORY Read list of words, subject must repeat them. Do 2 trials, even if 1st trial is successful. Do a recall after 5 minutes.

	FACE	VELVET	CHURCH	DAISY	RED	
1st trial						No points
2nd trial						

ATTENTION Read list of digits (1 digit/ sec.). Subject has to repeat them in the forward order [ ] 2 1 8 5 4
Subject has to repeat them in the backward order [ ] 7 4 2 — __/2

Read list of letters. The subject must tap with his hand at each letter A. No points if ≥ 2 errors
[ ] F B A C M N A A J K L B A F A K D E A A A J A M O F A A B — __/1

Serial 7 subtraction starting at 100 [ ] 93 [ ] 86 [ ] 79 [ ] 72 [ ] 65
4 or 5 correct subtractions: **3 pts**, 2 or 3 correct: **2 pts**, 1 correct: **1 pt**, 0 correct: **0 pt** — __/3

LANGUAGE Repeat : I only know that John is the one to help today. [ ]
The cat always hid under the couch when dogs were in the room. [ ] — __/2

Fluency / Name maximum number of words in one minute that begin with the letter F [ ] ____ (N ≥ 11 words) — __/1

ABSTRACTION Similarity between e.g. banana - orange = fruit [ ] train – bicycle [ ] watch - ruler — __/2

**DELAYED RECALL**	Has to recall words WITH NO CUE	FACE [ ]	VELVET [ ]	CHURCH [ ]	DAISY [ ]	RED [ ]	Points for UNCUED recall only	__/5
**Optional**	Category cue							
	Multiple choice cue							

ORIENTATION [ ] Date [ ] Month [ ] Year [ ] Day [ ] Place [ ] City — __/6

© Z.Nasreddine MD www.mocatest.org Normal ≥ 26 / 30 TOTAL __/30

Administered by: Add 1 point if ≤ 12 yr edu

APPENDIX 4: NIH STROKE SCALE

NIH STROKE SCALE

Patient Identification. __ __-__ __ __-__ __ __

Pt. Date of Birth __ __/__ __/__ __

Hospital ____________________(__ __-__ __)

Date of Exam __ __/__ __/__ __

Interval: [ ] Baseline [ ] 2 hours post treatment [ ] 24 hours post onset of symptoms ±20 minutes [ ] 7-10 days
[ ] 3 months [ ] Other ____________________(__ __)

Time: __ __:__ __ [ ]am [ ]pm

Person Administering Scale ____________________

Administer stroke scale items in the order listed. Record performance in each category after each subscale exam. Do not go back and change scores. Follow directions provided for each exam technique. Scores should reflect what the patient does, not what the clinician thinks the patient can do. The clinician should record answers while administering the exam and work quickly. Except where indicated, the patient should not be coached (i.e., repeated requests to patient to make a special effort).

Instructions	Scale Definition	Score
**1a. Level of Consciousness:** The investigator must choose a response if a full evaluation is prevented by such obstacles as an endotracheal tube, language barrier, orotracheal trauma/bandages. A 3 is scored only if the patient makes no movement (other than reflexive posturing) in response to noxious stimulation.	0 = **Alert;** keenly responsive.<br>1 = **Not alert;** but arousable by minor stimulation to obey, answer, or respond.<br>2 = **Not alert;** requires repeated stimulation to attend, or is obtunded and requires strong or painful stimulation to make movements (not stereotyped).<br>3 = Responds only with reflex motor or autonomic effects or totally unresponsive, flaccid, and areflexic.	____
**1b. LOC Questions:** The patient is asked the month and his/her age. The answer must be correct - there is no partial credit for being close. Aphasic and stuporous patients who do not comprehend the questions will score 2. Patients unable to speak because of endotracheal intubation, orotracheal trauma, severe dysarthria from any cause, language barrier, or any other problem not secondary to aphasia are given a 1. It is important that only the initial answer be graded and that the examiner not "help" the patient with verbal or non-verbal cues.	0 = **Answers** both questions correctly.<br>1 = **Answers** one question correctly.<br>2 = **Answers** neither question correctly.	____
**1c. LOC Commands:** The patient is asked to open and close the eyes and then to grip and release the non-paretic hand. Substitute another one step command if the hands cannot be used. Credit is given if an unequivocal attempt is made but not completed due to weakness. If the patient does not respond to command, the task should be demonstrated to him or her (pantomime), and the result scored (i.e., follows none, one or two commands). Patients with trauma, amputation, or other physical impediments should be given suitable one-step commands. Only the first attempt is scored.	0 = **Performs** both tasks correctly.<br>1 = **Performs** one task correctly.<br>2 = **Performs** neither task correctly.	____
**2. Best Gaze:** Only horizontal eye movements will be tested. Voluntary or reflexive (oculocephalic) eye movements will be scored, but caloric testing is not done. If the patient has a conjugate deviation of the eyes that can be overcome by voluntary or reflexive activity, the score will be 1. If a patient has an isolated peripheral nerve paresis (CN III, IV or VI), score a 1. Gaze is testable in all aphasic patients. Patients with ocular trauma, bandages, pre-existing blindness, or other disorder of visual acuity or fields should be tested with reflexive movements, and a choice made by the investigator. Establishing eye contact and then moving about the patient from side to side will occasionally clarify the presence of a partial gaze palsy.	0 = **Normal.**<br>1 = **Partial gaze palsy;** gaze is abnormal in one or both eyes, but forced deviation or total gaze paresis is not present.<br>2 = **Forced deviation,** or total gaze paresis not overcome by the oculocephalic maneuver.	____

Rev 10/1/2003

NIH STROKE SCALE

Patient Identification. ___ ___-___ ___ ___-___ ___ ___

Pt. Date of Birth ___ ___/___ ___/___ ___

Hospital ____________________(___ ___-___ ___)

Date of Exam ___ ___/___ ___/___ ___

Interval: [ ] Baseline [ ] 2 hours post treatment [ ] 24 hours post onset of symptoms ±20 minutes [ ] 7-10 days
[ ] 3 months [ ] Other ________________________________(___ ___)

**3. Visual:** Visual fields (upper and lower quadrants) are tested by confrontation, using finger counting or visual threat, as appropriate. Patients may be encouraged, but if they look at the side of the moving fingers appropriately, this can be scored as normal. If there is unilateral blindness or enucleation, visual fields in the remaining eye are scored. Score 1 only if a clear-cut asymmetry, including quadrantanopia, is found. If patient is blind from any cause, score 3. Double simultaneous stimulation is performed at this point. If there is extinction, patient receives a 1, and the results are used to respond to item 11.	0 = **No visual loss.**<br>1 = **Partial hemianopia.**<br>2 = **Complete hemianopia.**<br>3 = **Bilateral hemianopia** (blind including cortical blindness).	_____
**4. Facial Palsy:** Ask – or use pantomime to encourage – the patient to show teeth or raise eyebrows and close eyes. Score symmetry of grimace in response to noxious stimuli in the poorly responsive or non-comprehending patient. If facial trauma/bandages, orotracheal tube, tape or other physical barriers obscure the face, these should be removed to the extent possible.	0 = **Normal** symmetrical movements.<br>1 = **Minor paralysis** (flattened nasolabial fold, asymmetry on smiling).<br>2 = **Partial paralysis** (total or near-total paralysis of lower face).<br>3 = **Complete paralysis** of one or both sides (absence of facial movement in the upper and lower face).	_____
**5. Motor Arm:** The limb is placed in the appropriate position: extend the arms (palms down) 90 degrees (if sitting) or 45 degrees (if supine). Drift is scored if the arm falls before 10 seconds. The aphasic patient is encouraged using urgency in the voice and pantomime, but not noxious stimulation. Each limb is tested in turn, beginning with the non-paretic arm. Only in the case of amputation or joint fusion at the shoulder, the examiner should record the score as untestable (UN), and clearly write the explanation for this choice.	0 = **No drift;** limb holds 90 (or 45) degrees for full 10 seconds.<br>1 = **Drift;** limb holds 90 (or 45) degrees, but drifts down before full 10 seconds; does not hit bed or other support.<br>2 = **Some effort against gravity;** limb cannot get to or maintain (if cued) 90 (or 45) degrees, drifts down to bed, but has some effort against gravity.<br>3 = **No effort against gravity;** limb falls.<br>4 = **No movement.**<br>UN = **Amputation** or joint fusion, explain: ________________<br><br>**5a. Left Arm**<br>**5b. Right Arm**	 <br><br><br><br><br><br><br>_____<br>_____
**6. Motor Leg:** The limb is placed in the appropriate position: hold the leg at 30 degrees (always tested supine). Drift is scored if the leg falls before 5 seconds. The aphasic patient is encouraged using urgency in the voice and pantomime, but not noxious stimulation. Each limb is tested in turn, beginning with the non-paretic leg. Only in the case of amputation or joint fusion at the hip, the examiner should record the score as untestable (UN), and clearly write the explanation for this choice.	0 = **No drift;** leg holds 30-degree position for full 5 seconds.<br>1 = **Drift;** leg falls by the end of the 5-second period but does not hit bed.<br>2 = **Some effort against gravity;** leg falls to bed by 5 seconds, but has some effort against gravity.<br>3 = **No effort against gravity;** leg falls to bed immediately.<br>4 = **No movement.**<br>UN = **Amputation** or joint fusion, explain: ______________<br><br>**6a. Left Leg**<br>**6b. Right Leg**	 <br><br><br><br><br><br><br>_____

Rev 10/1/2003

NIH STROKE SCALE

Patient Identification. __ __-__ __ __-__ __ __

Pt. Date of Birth __ __/__ __/__ __

Hospital ________________(__ __-__ __)

Date of Exam __ __/__ __/__ __

Interval: [ ] Baseline [ ] 2 hours post treatment [ ] 24 hours post onset of symptoms ±20 minutes [ ] 7-10 days
[ ] 3 months [ ] Other __________________(__ __)

**7. Limb Ataxia:** This item is aimed at finding evidence of a unilateral cerebellar lesion. Test with eyes open. In case of visual defect, ensure testing is done in intact visual field. The finger-nose-finger and heel-shin tests are performed on both sides, and ataxia is scored only if present out of proportion to weakness. Ataxia is absent in the patient who cannot understand or is paralyzed. Only in the case of amputation or joint fusion, the examiner should record the score as untestable (UN), and clearly write the explanation for this choice. In case of blindness, test by having the patient touch nose from extended arm position.	0 = **Absent.**<br>1 = **Present in one limb.**<br>2 = **Present in two limbs.**<br>UN = **Amputation** or joint fusion, explain: ________	____
**8. Sensory:** Sensation or grimace to pinprick when tested, or withdrawal from noxious stimulus in the obtunded or aphasic patient. Only sensory loss attributed to stroke is scored as abnormal and the examiner should test as many body areas (arms [not hands], legs, trunk, face) as needed to accurately check for hemisensory loss. A score of 2, "severe or total sensory loss," should only be given when a severe or total loss of sensation can be clearly demonstrated. Stuporous and aphasic patients will, therefore, probably score 1 or 0. The patient with brainstem stroke who has bilateral loss of sensation is scored 2. If the patient does not respond and is quadriplegic, score 2. Patients in a coma (item 1a=3) are automatically given a 2 on this item.	0 = **Normal;** no sensory loss.<br>1 = **Mild-to-moderate sensory loss;** patient feels pinprick is less sharp or is dull on the affected side; or there is a loss of superficial pain with pinprick, but patient is aware of being touched.<br>2 = **Severe to total sensory loss;** patient is not aware of being touched in the face, arm, and leg.	____
**9. Best Language:** A great deal of information about comprehension will be obtained during the preceding sections of the examination. For this scale item, the patient is asked to describe what is happening in the attached picture, to name the items on the attached naming sheet and to read from the attached list of sentences. Comprehension is judged from responses here, as well as to all of the commands in the preceding general neurological exam. If visual loss interferes with the tests, ask the patient to identify objects placed in the hand, repeat, and produce speech. The intubated patient should be asked to write. The patient in a coma (item 1a=3) will automatically score 3 on this item. The examiner must choose a score for the patient with stupor or limited cooperation, but a score of 3 should be used only if the patient is mute and follows no one-step commands.	0 = **No aphasia;** normal.<br>1 = **Mild-to-moderate aphasia;** some obvious loss of fluency or facility of comprehension, without significant limitation on ideas expressed or form of expression. Reduction of speech and/or comprehension, however, makes conversation about provided materials difficult or impossible. For example, in conversation about provided materials, examiner can identify picture or naming card content from patient's response.<br>2 = **Severe aphasia;** all communication is through fragmentary expression; great need for inference, questioning, and guessing by the listener. Range of information that can be exchanged is limited; listener carries burden of communication. Examiner cannot identify materials provided from patient response.<br>3 = **Mute, global aphasia;** no usable speech or auditory comprehension.	____
**10. Dysarthria:** If patient is thought to be normal, an adequate sample of speech must be obtained by asking patient to read or repeat words from the attached list. If the patient has severe aphasia, the clarity of articulation of spontaneous speech can be rated. Only if the patient is intubated or has other physical barriers to producing speech, the examiner should record the score as untestable (UN), and clearly write an explanation for this choice. Do not tell the patient why he or she is being tested.	0 = **Normal.**<br>1 = **Mild-to-moderate dysarthria;** patient slurs at least some words and, at worst, can be understood with some difficulty.<br>2 = **Severe dysarthria;** patient's speech is so slurred as to be unintelligible in the absence of or out of proportion to any dysphasia, or is mute/anarthric.<br>UN = **Intubated** or other physical barrier, explain: ________	____

Rev 10/1/2003

N I H STROKE SCALE

Patient Identification. ___ ___-___ ___ ___-___ ___ ___

Pt. Date of Birth ___ ___/___ ___/___ ___

Hospital ____________________(___ ___-___ ___)

Date of Exam ___ ___/___ ___/___ ___

Interval: [ ] Baseline [ ] 2 hours post treatment [ ] 24 hours post onset of symptoms ±20 minutes [ ] 7-10 days
[ ] 3 months [ ] Other ____________________(___ ___)

**11. Extinction and Inattention (formerly Neglect):** Sufficient information to identify neglect may be obtained during the prior testing. If the patient has a severe visual loss preventing visual double simultaneous stimulation, and the cutaneous stimuli are normal, the score is normal. If the patient has aphasia but does appear to attend to both sides, the score is normal. The presence of visual spatial neglect or anosagnosia may also be taken as evidence of abnormality. Since the abnormality is scored only if present, the item is never untestable.	0 = **No abnormality.**<br>1 = **Visual, tactile, auditory, spatial, or personal inattention** or extinction to bilateral simultaneous stimulation in one of the sensory modalities.<br>2 = **Profound hemi-inattention or extinction to more than one modality;** does not recognize own hand or orients to only one side of space.	____

____

____

Rev 10/1/2003

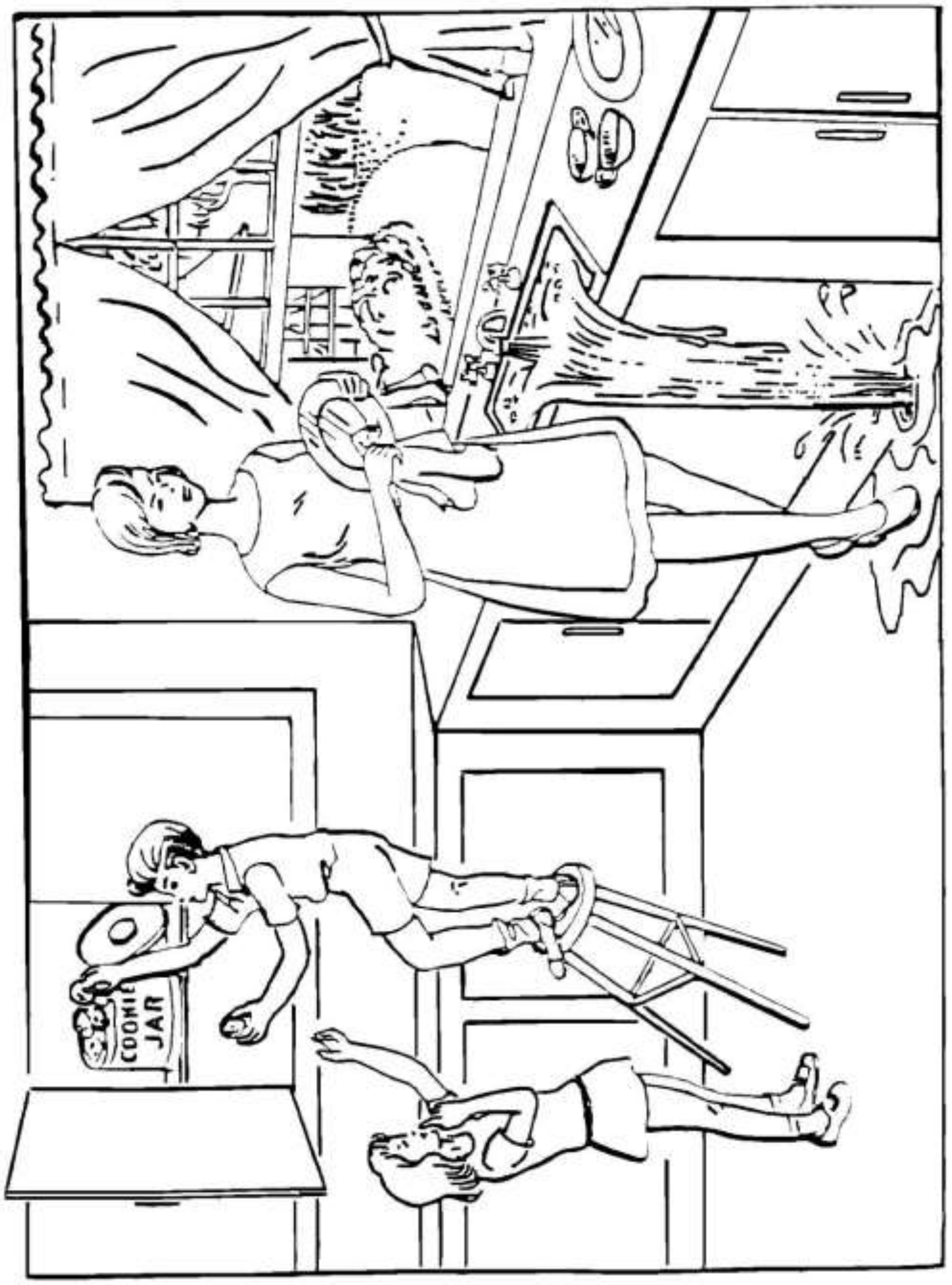
COOKIE JAR

You know how.

Down to earth.

I got home from work.

Near the table in the dining room.

They heard him speak on the radio last night.

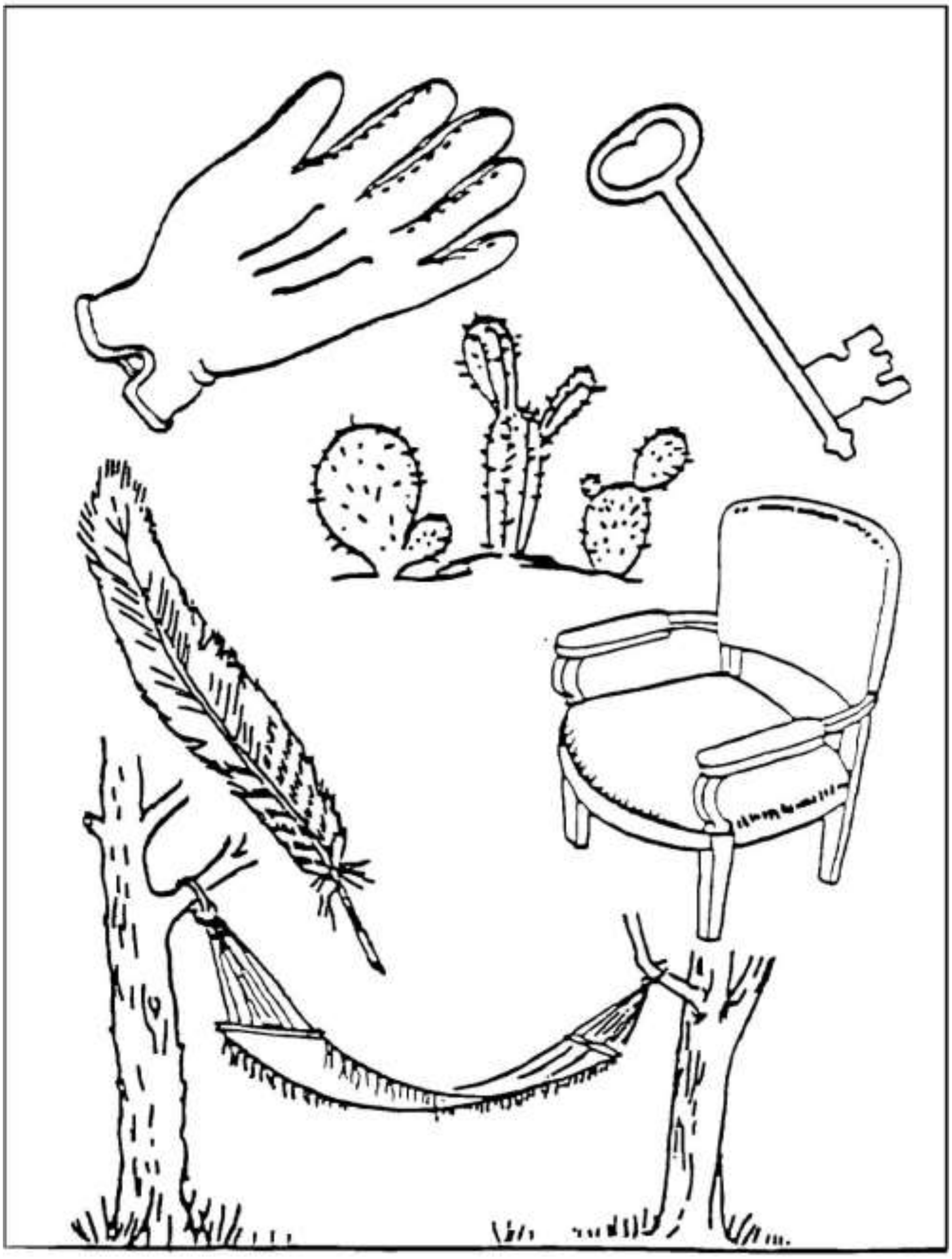

MAMA

TIP – TOP

FIFTY – FIFTY

THANKS

HUCKLEBERRY

BASEBALL PLAYER

APPENDIX 5: EQ5D-5L

EQ-5D-5L

Sample

Health Questionnaire

English version for the UK

UK (English) © 2009 EuroQol Group EQ-5D™ is a trade mark of the EuroQol Group

Under each heading, please tick the ONE box that best describes your health TODAY.

MOBILITY

I have no problems in walking about ☐

I have slight problems in walking about ☐

./ I have moderate problems in walking about ☐

I have severe problems in walking about ☐

I am unable to walk about ☐

SELF-CARE

I have no problems washing or dressing myself ☐

I have slight problems washing or dressing myself ☐

I have moderate problems washing or dressing myself ☐

I have severe problems washing or dressing myself ☐

I am unable to wash or dress myself ☐

USUAL ACTIVITIES *(e.g. work, study, housework, family or leisure activities)*

I have no problems doing my usual activities ☐

I have slight problems doing my usual activities ☐

I have moderate problems doing my usual activities ☐

I have severe problems doing my usual activities ☐

I am unable to do my usual activities ☐

PAIN / DISCOMFORT

I have no pain or discomfort ☐

I have slight pain or discomfort ☐

I have moderate pain or discomfort ☐

I have severe pain or discomfort ☐

I have extreme pain or discomfort ☐

ANXIETY / DEPRESSION

I am not anxious or depressed ☐

I am slightly anxious or depressed ☐

I am moderately anxious or depressed ☐

I am severely anxious or depressed ☐

I am extremely anxious or depressed ☐

Sample

UK (English) © 2009 EuroQol Group EQ-5D™ is a trade mark of the EuroQol Group

- We would like to know how good or bad your health is TODAY.
- This scale is numbered from 0 to 100.
- 100 means the <u>best</u> health you can imagine.
  0 means the <u>worst</u> health you can imagine.
- Mark an X on the scale to indicate how your health is TODAY.
- Now, please write the number you marked on the scale in the box below.

YOUR HEALTH TODAY = ☐

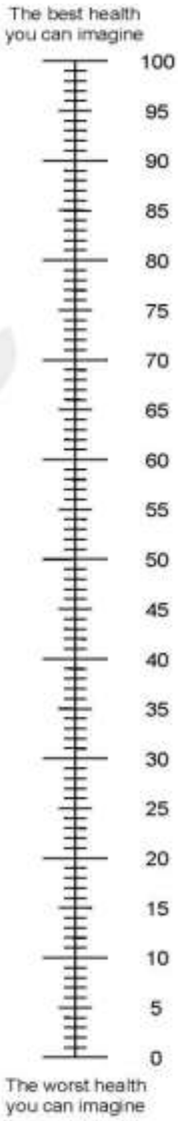

Sample

3

UK (English) © 2009 EuroQol Group EQ-5D™ is a trade mark of the EuroQol Group

APPENDIX 6: UNIVERSAL DEFINITION OF MYOCARDIAL INFARCTION, TIMI DEFINITION FOR HOSPITALIZATION OF UNSTABLE ANGINA

Myocardial Infarction: Third -Universal Definition (22)

Criteria for acute myocardial infarction
The term acute MI should be used when there is evidence of myocardial necrosis in a clinical setting consistent with acute myocardial ischemia. Under these conditions any one of the following criteria meets the diagnosis for MI:

- ***Detection of a rise and/or fall of cardiac biomarker values*** (preferably cardiac troponin [cTn]) with at least one value above the 99th percentile upper reference limit (URL) ***and with at least one of the following***:
    - Symptoms of ischemia.
    - New or presumed new significant ST-segment–T wave (ST–T) changes or new left bundle branch block (LBBB).
    - Development of pathological Q waves in the ECG.
    - Imaging evidence of new loss of viable myocardium or new regional wall motion abnormality.
    - Identification of an intracoronary thrombus by angiography or autopsy
- ***Cardiac death with symptoms suggestive of myocardial ischemia and presumed new ischemic ECG changes or new LBBB***, but death occurred before cardiac biomarkers were obtained, or before cardiac biomarker values would be increased
- ***PCI- related MI*** is arbitrarily defined by elevation of cTn values (>5 x 99th percentile URL) in patients with normal baseline values (≤99th percentile URL) or a rise of cTn values >20% if the baseline values are elevated and are stable or falling. In addition, either (i) symptoms suggestive of myocardial ischemia or (ii) new ischemic ECG changes or (iii) angiographic findings consistent with a procedural complication, or (iv) imaging demonstration of new loss of viable myocardium or new regional wall motion abnormality are required
- Stent thrombosis associated with MI when detected by coronary angiography or autopsy in the setting of myocardial ischemia and with a rise and/or fall of cardiac biomarker values with at least one value above the 99th percentile URL

CABG- related MI is arbitrarily defined by elevation of cardiac biomarker values (>10 x 99th percentile URL) in patients with normal baseline cTn values (≤99th percentile URL).
In addition, either

(i) new pathological Q waves or new LBBB,
or
(ii) angiographic documented new graft or new native coronary artery occlusion,
or
(iii) imaging evidence of new loss of viable myocardium or new regional wall motion abnormality

APPENDIX 6: TIMI DEFINITION OF HOSPITALIZATION FOR UNSTABLE ANGINA

Unstable angina requiring hospitalization is defined as:

1) Ischemic discomfort (angina, or symptoms thought to be equivalent) ≥10 minutes in duration occurring

- At rest, or
- In an accelerating pattern with frequent episodes associated with progressively decreased exercise capacity

AND

2) Prompting an unscheduled hospitalization <u>within 24 hours</u> of the most recent symptoms. Hospitalization is defined as an admission to an inpatient unit or a visit to an emergency department that results in at least a 24* hour stay (or a change in calendar date if the hospital admission or discharge times are not available)

AND

3. At least one of the following:

a) New or worsening ST or T wave changes on resting ECG (in the absence of confounders, such as LBBB or LVH)

Transient ST elevation (duration < 20 minutes)
New ST elevation at the J point in two contiguous leads with the cut-points: ≥ 0.1 mV in all leads other than leads V2-V3 where the following cut-points apply: ≥0.2 mV in men ≥ 40 years, or, 0.15 mV in women.

ST depression and T-wave changes.
New horizontal or down-sloping ST depression ≥0.05 mV in two contiguous leads and/or new T inversion ≥0.3V mV in two contiguous leads with prominent R wave or R/S ratio > 1.

b) Definite evidence of inducible myocardial ischemia as demonstrated by an early positive exercise stress test, defined as ST elevation or ≥2mm ST depression prior to 5 mets

OR

Stress echocardiography (reversible wall motion abnormality)

OR

Myocardial scintigraphy (reversible perfusion defect)

OR

MRI (myocardial perfusion deficit under pharmacologic stress)

<u>And</u> believed to be responsible for the myocardial ischemic symptoms/signs

c) Angiographic evidence of new or worse ≥70% lesion and/or thrombus in an epicardial coronary artery that is believed to be responsible for the myocardial ischemic symptoms/signs

d) Need for coronary revascularization procedure (PCI or CABG) for the presumed culprit lesion(s). This criterion would be fulfilled if revascularization was undertaken during the unscheduled hospitalization, or subsequent to transfer to another institution without interceding home discharge.

AND

4. Negative cardiac biomarkers and no evidence of acute MI

APPENDIX 7: EXPECTED ADVERSE EVENTS AFTER STROKE

The following adverse events occur with increased frequency after stroke. If such event(s) occur in a trial subject, the event should be considered an expected stroke-related adverse event, unless there is a reasonable likelihood that the event was caused by study medication (colchicine or usual care) in the opinion of the Site Investigator or Coordinating Centre medical expert.

Death

Neurological:

Brain oedema/swelling

Haemorrhagic transformation/Intracerebral haematoma/Intracerebral haemorrhage

Subarachnoid/intraventricular haemorrhage, de novo or extension from intracerebral haemorrhage

Seizures

Epilepsy

Recurrent stroke

Pain syndromes

Delerium/Confusion/Agitation

Cognitive decline/Dementia

Headache

Insomnia

Somnolence/Drowsiness/Coma

Cardiovascular:

Congestive cardiac failure

Arrhythmias, including but not limited to: atrial fibrillation, atrial flutter, supraventricular tachycardia, ectopic beats, ventricular tachycardia, ventricular fibrillation, prolonged QT interval, atrio-ventricular block, cardiac arrest

Myocardial infarction/Angina, requiring medical treatment and/or revascularisation

Cardiomyopathy

Respiratory:
Pneumonia/Pneumonitis

Atelectasis

Pleural effusion

Bronchial plugging

Hypoxia/Anoxia

Pulmonary embolism

Apnoea

Obstructive sleep apnoea

Gastro-intestinal/Hepatic:

Dysphagia

Gastro-intestinal bleeding

Faecal incontinence

Diarrhoea

Constipation

Weight loss

Anorexia

Heartburn/Acid reflux

Abdominal discomfort

Abdominal distension/bloating

Abnormal liver function tests

Renal/Genito-urinary:

Acute renal dysfunction

Dehydration

Peripheral oedema

Urinary tract infection

Urinary incontinence

Urinary urgency

Dysuria/Haematuria

Haematologic:

Deep venous thrombosis

Anaemia

Thrombocytopenia/Thrombocytosis

Raised white blood cell count/Leucocytosis

Raised inflammatory markers (CRP, erythrocyte sedimentation rate)

Fever

Dermatological:

Rash

Decubitus ulcers

Itch

Musculoskeletal:

Falls

Fractures

Shoulder dislocation/shoulder pain

Osteopenia/osteoporosis

Mental health:

Fatigue

Depression

Anxiety

Agitation

References:

1. Balami JS, et al. Neurological complications of acute ischaemic stroke. Lancet Neurol 2011;10:357-71

2. Kumar S, et al. Medical complications after stroke. Lancet Neurol 2010;9:105-18

Appendix 8: Expected adverse events after colchicine

Expected adverse effects of colchicine, from Actavis UK SmPC:

The following adverse reactions have been observed with higher doses of colchicine than are being used in the CONVINCE trial. In studies of low-dose colchicine, similar to the dose used in CONVINCE, significant adverse reactions rarely occurred.

The frequencies are listed under one of the following classifications:

Very common > 1/10

Common > 1/100 and < 1/10

Uncommon > 1/1000 and < 1/100

Rare > 1/10 000 and < 1/1000

Very rare < 1/10 000

Not known (cannot be estimated from the available

data) Blood and lymphatic system disorders

Not known: bone marrow depression with agranulocytosis, aplastic anemia and thrombocytopenia.

Nervous system disorders

Not known: peripheral neuritis, neuropathy.

Gastrointestinal system disorders

Common: abdominal pain, nausea, vomiting and

diarrhoea. Not known: gastrointestinal haemorrhage.

Hepatobiliary disorders

Not known: hepatic damage.

Skin and subcutaneous tissue disorders

Not known: alopecia, rash.

Musculoskeletal and connective tissue disorders

Not known: myopathy and rhabdomyolysis.

Renal and urinary disorders

Not known: renal damage.

Reproductive system and breast

disorders

Not known: amenorrhoea, dysmenorrhoea, oligospermia, azoospermia.

APPENDIX 9: MINIMUM SOURCE DOCUMENTS FOR OUTCOME ADJUDICATION

Primary outcome components – non-fatal ischaemic stroke:

Mandatory:

1. Clinical note or letter from treating physician, describing nature of new focal neurological deficit. The duration of the deficit must be greater than 24 hours, either clearly stated or in the opinion of the Outcome Committee from information provided, or less than 24 hours with MRI evidence of acute infarction.

Not mandatory, but to be collected for Outcome Committee review if performed:

1. Report of brain MRI, as soon as possible after new event
2. Report of brain CT, as soon as possible after new event

Primary outcome components – retinal infarction:

Mandatory:

1. Clinical note or letter from treating physician, describing new acute monocular visual loss. The duration of the visual deficit must be greater than 24 hours, either clearly stated or in the opinion of the Outcome Committee from information provided, Branch or central retinal or ophthalmic artery occlusion must be verified by an ophthalmologist. Anterior ischaemic optic neuropathy is excluded.

Primary outcome components – spinal cord infarction:

Mandatory:

1. Clinical note or letter from treating physician, describing new acute neurological deficit attributable to spinal cord dysfunction. The duration of the visual deficit must be greater than 24 hours, either clearly stated or in the opinion of the Outcome Committee from information provided,

2. Spinal cord imaging to exclude mimic syndromes of cord infarction.

Primary outcome components – non-fatal myocardial infarction:

Mandatory:

1. Report of cardiac biomarker values (preferably cardiac troponin [cTn]) with at least one value above the 99th percentile upper reference limit (URL)

2. Plus at least one of the following:

(a) Physician letter or note documenting symptoms of cardiac ischemia.

(b) Copy of ECG, or letter or report referring to ECG, obtained after suspected MI: This must show, new or presumed new significant ST-segment–T wave (ST–T) changes or new left bundle branch block (LBBB), or development of pathological Q waves in the ECG.

(c) Copy of imaging report, or letter or report referring to imaging results, detailing imaging evidence of new loss of viable myocardium or new regional wall motion abnormality.

(d) Copy of report of angiogram, or letter or report referring to angiogram results, detailing identification of an intracoronary thrombus

Primary outcome components – non-fatal cardiac arrest:

Mandatory:

(1) Physician letter or note documenting recovery from sudden collapse

(2) Copy of ECG or rhythm strip, or letter or report referring to ECG or rhythm strip, obtained at the time of collapse, showing cardiac asystole, ventricular tachycardia, or ventricular fibrillation

Primary outcome components – Hospitalization for Unstable Angina:

Mandatory:

(1) Clinical note/letter from treating physician documenting hospitalization for unstable angina
(2) At least one of the following: copy of ECG OR rhythm strip OR stress echocardiography OR myocardial scintigraphy OR MRI OR angiogram
(3) Report of negative cardia biomarkers Autopsy report, or physician letter or note documenting death caused by non-haemorrhagic stroke, cardiac arrest or myocardial infarction, without other identified cause, the onset of which was within the previous 30 days

Primary outcome components – Vascular death:

Mandatory:

(1) Autopsy report, or physician letter or note documenting death caused by non-haemorrhagic stroke, cardiac arrest or myocardial infarction, without other identified cause, the onset of which was within the previous 30 days

Non-mandatory:

(A) If death by non-haemorrhagic stroke, onset within the past 30 days:

Reports of brain MRI and CT should be provided, if performed

(B) If death by myocardial infarction, onset within the past 30 days:

The following reports should be provided, if available:

(i) Reports of cardiac biomarkers

(ii) Physician note documenting symptoms of cardiac ischaemia

(iii) Copy of ECG, or note/report referring to ECG, showing new LBBB, ST-T changes, or new pathological Q waves

(iv) Copy of imaging report, or note/report referring to imaging, showing new loss of viable myocardium or new regional wall motion abnormality

(v) Copy of report of angiogram, or letter or report referring to angiogram results, detailing identification of an intracoronary thrombus

(C) If death by cardiac arrest, onset within the past 30 days:

The following reports should be provided, if available:

(i) Physician letter or note documenting sudden collapse

(ii) Copy of ECG or rhythm strip, or letter or report referring to ECG or rhythm strip, obtained at the time of collapse, showing cardiac asystole, ventricular tachycardia, or ventricular fibrillation

Secondary Outcomes:

1. Safety: Data will be provided as required on the Adverse Event form, and additional safety reporting requirements for Serious Adverse Reactions and Suspected Unexpected Serious Adverse Reactions
2. Disability: Modified Rankin score data obtained according to the Study Schedule and entered on the eCRF will be used
3. Cognition: MOCA data obtained according to Study Schedule will be used
4. Quality of Life: EQ5D data obtained according to Study Schedule will be used

APPENDIX 10: CREATININE CLEARANCE BY MDRD FORMULA

Modification of diet in renal disease (MDRD) equation (eGFR = 175 x $SerumCr^{-1.154}$ × $age^{-0.203}$ ×1.212 [if patient is black] × 0.742 [if female]).

APPENDIX 11: CREATININE CLEARANCE BY CKD-EPI FORMULA

The CKD Epidemiological Collaboration (CKD-EPI) equation (GFR = 141 X min(Scr/κ,1)^{α} X max(Scr/κ,1)$^{-1.209}$ × 0.993^{Age} X 1.018 [if female] × 1.159 [if black]), where Scr is serum creatinine, κ is 0.7 for females and 0.9 for males, α is -0.329 for females and -0.411 for males, min indicates the minimum of Scr/κor 1, and max indicates the maximum of Scr/κ or 1

APPENDIX 12: Detail of all changes applied to Protocol Version 3.0

CHANGES FROM PROTOCOL VERSION 1: 4 May 2016 and VERSION 2: 31 August 2016 and VERSION 2.1: 29 November 2016 and Version 2.2 30 JANUARY 2019 and Version 2.3 20 February 2019 Version 2.4 03 April 2019 to Version 2.5 11 February 2020 to current Version 3.0 12 August 2020

EudraCT No: 2015-004505-16

**Item no.**	**Previous and new wording in track change modus**<br>Deleted Text = ~~in red with strikethrough~~	**New wording**<br><br>New wording **=** **<u>in red and underlined</u>**	**Comments/explanation/ reasons for substantial amendment**
1	To investigate the efficacy of low dose colchicine (0.5mg/day) plus usual care (defined as antiplatelet, lipid-lowering, antihypertensive treatment, and appropriate lifestyle advice) compared with usual care alone to prevent non-fatal recurrent ischaemic stroke, myocardial infarction, cardiac arrest, and vascular death after ischaemic stroke or transient ischaemic attack (TIA) not caused by cardiac embolism or other defined causes unrelated to atherosclerosis	Primary Objective of 'hospitalization for unstable angina' **added to primary objectives**. This outcome has been added to sections 7.1 (study objectives) , 8.1.4 (Outcome measures), 9.1 (Trial Design – general measures)<br><br>To investigate the efficacy of low dose colchicine (0.5mg/day) plus usual care (defined as antiplatelet, lipid-lowering, antihypertensive treatment, and appropriate lifestyle advice) compared with usual care alone to prevent non-fatal recurrent ischaemic stroke, myocardial infarction, cardiac arrest**<u>, hospitalization for unstable angina</u>** and vascular death after ischaemic stroke or transient ischaemic attack (TIA) not caused by cardiac embolism or other defined causes unrelated to atherosclerosis	Unstable angina requiring hospitalisation is a significant cardiac outcome which may be reduced by colchicine therapy. It has been added to the outcome cluster to reflect its importance for patients and to enhance the statistical power of the trial
2	Inclusion Criterion # 3<br>3. Either,<br>• ischaemic stroke without major disability (modified Rankin score *3 or less*)<br>• ***or*** high-risk TIA	3. Patient has had either;-<br>An ischaemic stroke without major disability (modified Rankin score *3 or less*)<br>***or***<br>***a*** high-risk TIA<u>*</u>	Re-worded the text to make the criterion clearer to read and interpret.

	~~within 72 hours-28 days of randomisation~~<br>and<br>brain CT or MRI has excluded primary intracranial haemorrhage<br>High-risk TIA is defined as transient focal neurological symptoms of presumed vascular cause with, in addition, one or more of the following criteria:<br>(a) ABCD2 score 4 or more, with motor or speech symptoms (dysarthria or dysphasia)<br>(b) DWI hyperintensity on acute MRI<br>(c) Stenosis (lumen narrowing of 50% or greater on ultrasound, MRA, CTA, or invasive angiography) of the internal carotid, vertebral, middle cerebral, anterior cerebral, or basilar artery in the arterial territory consistent with symptoms	AND<br>A brain CT or MRI has excluded primary intracranial haemorrhage<br><br>AND<br>The stroke/TIA has occurred more than 72 hours before randomisation AND no more than 28 days prior to randomisation<br>**High-risk TIA is defined as transient focal neurological symptoms of presumed vascular cause with, in addition, one or more of the following criteria:*<br>*ABCD2 score 4 or more, with motor or speech symptoms (dysarthria or dysphasia)*<br>*DWI hyperintensity on acute MRI*<br>*Stenosis (lumen narrowing of 50% or greater on ultrasound, MRA, CTA, or invasive angiography) of the internal carotid, vertebral, middle cerebral, anterior cerebral, or basilar artery in the arterial territory consistent with symptoms*	
3	Key Exclusion Criteria<br>: highlighted text below was removed<br><br>1. ~~Cardio-embolic~~ stroke/TIA, probably caused by identified atrial fibrillation (permanent or paroxysmal), in the opinion of the treating physician.<br><br>2. ~~Cardio-embolic~~ stroke/TIA probably caused by other identified cardiac source (intra-cardiac thrombus, endocarditis, metallic heart valve, low	Current Criteria<br><br>1. Stroke/TIA, probably caused by identified atrial fibrillation (permanent or paroxysmal), in the opinion of the treating physician.<br><br>2. stroke/TIA probably caused by other identified cardiac source (intra-cardiac thrombus, endocarditis, metallic heart valve, low ejection	Removed extraneous wording

	ejection fraction <30%), ~~in the opinion of the treating physician.~~<br><br>3. Stroke/TIA caused by dissection, endocarditis, paradoxical embolism, drug use, venous thrombosis, ~~within 48 hours after~~ carotid or cardiac surgery, hypercoagulability states, migraine, or inherited cerebrovascular disorders ~~(eg. Fabry's disease, CADASIL), in the opinion of the treating physician.~~<br><br>5. Blood dyscrasia ~~defined as anaemia~~ (haemoglobin <10g/dL~~), thrombocytopenia~~ (platelet count <150 x109/L) ~~or leucopenia~~ (white cell count <4 x109/L)~~at randomisation~~.<br><br>6. Impaired hepatic function (transaminases greater than twice upper limit of normal) ~~at randomisation.~~<br><br>10. Active malignancy, known hepatitis B or C, or HIV infection ~~prior to qualifying stroke/TIA.~~<br><br>11. Impaired swallow preventing oral administration of ~~study medication.~~	fraction <30%),<br><br>3. Stroke/TIA caused by dissection, endocarditis, paradoxical embolism, drug use, venous thrombosis, carotid or cardiac surgery, hypercoagulability states, migraine, or inherited cerebrovascular disorders.<br><br>5. Blood dyscrasia (haemoglobin <10g/dL,platelet count <150 x10^9/L,white cell count <4 x10^9/L)<br><br>6. Impaired hepatic function (transaminases greater than twice upper limit of normal)<br><br>11. Active malignancy, known hepatitis B or C, or HIV infection. (criterion was renumbered as there was an error on the original numbering)<br><br>12. Impaired swallow preventing oral administration of **Colchicine** (criterion was renumbered as there was an error on the original numbering)	

4	Exclusion Criteria #7:<br><br>7. Concurrent treatment with ~~moderate or strong~~ CYP3A4 inhibitors (clarithromycin, erythromycin, telithromycin, other macrolide antibiotics, ketoconazole, itraconazole, voriconazole, ritonavir, atazanavir, indinavir, other HIV protease inhibitors, verapamil, diltiazem, quinidine, digoxin, disulfiram) or P-gp inhibitors (cyclosporine) at randomisation.	Exclusion Criteria #7: updated bolded text<br><br>7. Concurrent treatment with **colchicine contraindicated** drugs:- CYP3A4 inhibitors (clarithromycin, erythromycin, telithromycin, other macrolide antibiotics, ketoconazole, itraconazole, voriconazole, ritonavir, atazanavir, indinavir, other HIV protease inhibitors, verapamil, diltiazem, quinidine, digoxin, disulfiram) or P-gp inhibitors (cyclosporine) at randomisation.	Clarification that this list of medications are contraindicated during administration of study drug: Colchicine.
5	18 Criteria in original protocol – numbering was incorrect as Criteria 9 was applied to 2 sequential criteria in error.	Exclusion Criteria renumbered correctly: 1-19	Correct numbering of Exclusion Criteria.
6	Control Arm: pg 21/90<br>Usual care only, defined as ~~anti-thrombotic~~, lipid-lowering, and anti-hypertensive treatment and lifestyle advice (smoking cessation, diet and physical activity), as deemed appropriate by the treating clinician.	Control Arm: pg 10/106<br>Usual care only, defined as anti-platelet, lipid-lowering, and anti-hypertensive treatment and lifestyle advice (smoking cessation, diet and physical activity), as deemed appropriate by the treating clinician.	Updated to be consistent - term of 'anti-platelet treatment' used throughout the protocol.
7	Duration of Treatment: page 10/90<br>Median approximately 36 months (range 12-60 months)	Duration of Treatment pg 10/106<br>Median approximately 36 months (**approximate** range 12-60 months)	Updated range to approximate
8	Section 9.1 pg 22/90<br><br>**Vanguard stage:** The first stage will be a Vanguard stage, during which it is planned to recruit approximately 265 patients at circa. 24 sites	Section 9.1, pg 20/106<br><br>**Vanguard stage:** The first stage will be a Vanguard stage, during which it is planned to recruit approximately 265 patients at circa. 24	265 patients needed to perform end-of-Vanguard analyses (i.e., 10% of complete trial enrolment). Specific duration removed to provide flexibility

	in Europe ~~with a target recruitment period of approximately one year.~~	sites in Europe.	with recruitment period
9	**Full Trial stage:** The next stage is called the Full Trial stage. This is expected to proceed after the outcome of the Vanguard stage review. Recruitment of a further 2,358 patients (approximately) will be performed at up to 200 hospital sites ~~over a further 2-year period approximately.~~ The entire sample size for the trial includes the 265 patients recruited in the Vanguard Stage plus the 2,358 recruited in the Full Trial Stage. Following close of recruitment, a further two years of follow-up will then take place. ~~Trial close-out and analysis is expected to take place in mid-2021~~. Including patients recruited in the Vanguard Stage, the longest follow-up period will be 60 months and shortest will be 24 months (median approximately 36 months).	**Full Trial stage:** The next stage is called the Full Trial stage. This is expected to proceed after the outcome of the Vanguard stage review. Recruitment of a further 2,358 patients (approximately) will be performed at up to 200 hospital sites. The entire sample size for the trial includes the 265 patients recruited in the Vanguard Stage plus the 2,358 recruited in the Full Trial Stage. Following close of recruitment, **an estimated** further two years of follow-up will then take place. Including patients recruited in the Vanguard Stage, the **estimated** longest follow-up period will be 60 months and shortest will be 24 months (median approximately 36 months**).** **Follow up for a longer period may be performed if recommended by the DMC and/or TSC.**	Removed specific dates to provide flexibility with recruitment and follow up period
10	Section 9.2.2 Inclusion and Section 9.2.3 Exclusion<br>Copied sections per items 3-5 above.	Section 9.2.2 Inclusion and Section 9.2.3 Exclusion<br>Copied sections per items 3-5 above.	
11	Section 9.2.4 Randomisation: 1st para<br>Randomisation will be conducted using ~~an automated~~ minimisation algorithm, to ensure groups are balanced for key prognostic variables affecting recurrent stroke risk.	Randomisation will be conducted using a minimisation algorithm, to ensure groups are balanced for key prognostic variables affecting recurrent stroke risk.	Minimisation will be a combination of biostatistician-generated algorithm (not automated algorithm) executing via interactive web randomising application
12	Randomisation 9.2.4, 3rd para<br>Randomisation will take place via an Interactive	Randomisation will take place via an Interactive	At this time a web based randomisation application is

	Web Response System (IWRS), ~~supplemented by an Interactive Voice Response System as needed.~~<br>Before randomisation occurs, informed consent and verification of trial eligibility by computer entry of<br>key inclusion/exclusion criteria ~~will~~ be performed by the site study staff.	Web Response System (IWRS),. Before randomisation occurs, informed consent and verification of trial eligibility of key inclusion/exclusion criteria must be performed by the site study staff.	planned and not a voice response system.
13		Section 9.2.4, new 4th para added<br><br>In practice, when a patient is randomised , the patient will be allocated to active treatment, or, usual care. If randomised to study medication, the patient will be given one dose from the next available package of study medication at the study site. This package (minus the first dose) and a second package will be given to the patient to take home.The patient will be advised to take the medication once daily and in the morning. If the dose is forgotten in the morning it can be taken at a later time in the day. The patient will also be instructed never to take more than one tablet per day even if a previous day/days dosage has been missed	Added detailed instructions regarding randomisation, first dose, dosing times and dispensing study medication.
14	Section 9.2.5 Investigations prior to enrolment in the trial<br>Certain investigations are mandatory prior to entry in the trial to determine patient eligibility. These are usually performed as the standard of usual care for patients with their qualifying stroke or TIA. They are listed here for clarity, ~~but are not included in the trial procedures~~.<br>***~~The following laboratory and/or imaging tests~~ are required within 28 days prior to trial entry to determine patient eligibility:***<br>1. Brain CT or MRI	Section 9.2.5 Investigations prior to enrolment in the trial<br>Certain investigations are **mandatory** prior to entry in the trial to determine patient eligibility. These are usually performed as the standard of usual care for patients with their qualifying stroke or TIA. They are listed below ***and are required within 28 days prior to trial entry:-***<br><br>Brain CT or MRI<br>eGFR (by MDRD or CKD-Epi method – Appendices 10,11)	Original protocol needed clarification on mandatory tests that are 'usual standard of care' and the timelines per protocol required for these tests.

	2. GFR (by MDRD or CKD-Epi method)<br>3. Liver transaminases (AST and ALT)<br>4. Full blood count<br>5. ECG or rhythm strip (a minimum requirement to exclude atrial fibrillation)	Liver transaminases (AST and ALT)<br>Hemoglobin, WBC, Platelets<br>ECG or rhythm strip (a minimum requirement to exclude atrial fibrillation)	
15	Section 9.2.5 non-mandatory tests<br><br>***The following investigations are not mandatory prior to trial entry:***	Section 9.2.5 non-mandatory tests<br><br>***The following investigations are not mandatory prior to trial entry but if they are performed please enter the results into the eCRF at the Baseline visit***	Simplified the wording in the section.
16	Section 9.2.5 Continuous cardiac monitoring<br><br>***Continuous cardiac monitoring (telemetry or ambulatory Holter monitoring) is not mandatory,*** ~~if atrial fibrillation is deemed unlikely as a cause of stroke/TIA in the opinion of the treating physician (eg. patients with lacunar stroke or those with identified carotid stenosis in the appropriate arterial territory).~~ If continuous cardiac monitoring is performed, a minimum duration of 20 hours is recommended and results should be entered on the CRF.	Section 9.2.5 Continuous cardiac monitoring<br><br>***Continuous cardiac monitoring (telemetry or ambulatory Holter monitoring) is not mandatory,*** If continuous cardiac monitoring is performed, a minimum duration of 20 hours is recommended	Simplified the wording in the section.
17		Section 9.2.5: #6 Non mandatory test of NIHSS added<br>**6. The NIH Stroke Scale** (Appendix 4) score (stroke patients only) is not mandatory, but if performed results will be recorded	Added this exam in non-mandatory section.
18	Section 9.3.1 Schedule of Visits<br>~~Study visits and assessments are summarised in Schedule of Events, Figure 2.~~	Section 9.3.1 Schedule of Visits<br>In order to standardise the number of weeks between visits for all patients in the study:	Visit Schedule was based on 'calendar months' in original protocol. It was not specified if

	~~Baseline and follow-up assessment data will be reported to the sponsor using the eCRF.~~	'weeks' rather than 'calendar months' are being used. Therefore, for this study **the definition of a month** is 4 weeks, a year is 52 weeks and a half year is 26 weeks. All visits for each patient are scheduled out for the entire study based on the randomisation date of each patient.<br><br>If for any reason a patient is late/too early for one visit, the subsequent planned visit is not to be adjusted: the patient should continue per the schedule planned at randomisation.<br><br>Study visits and assessments are summarised in the Task Schedule below<br><br>If a patient cannot attend for a visit the information can be taken over the phone with the patient and the visit completed in the e-CRF (the visit date = date of call)	visits were to be projected out from 'randomisation' or to be scheduled as each visit occurred. The protocol now calls for visits to be scheduled out for the entire study from the date of randomisation. A month is now defined as 4 weeks. This will help ensure that visits for all patients will occur within standardised intervals across the study. This change helps project planning and management in calculating end of study timelines for each patient.
19	Section 9.3.1 Figure 2 Task Flow<br>(old table too large to insert here)	Section 9.3.1 table too large to insert – see attached.<br>Renamed 'Schedule of Events' to 'Task Flow Schedule' and table comprehensively updated to include all assessments and visits at which assessments needed to be performed.	Original Task Flow did not include all assessments and needed to be more comprehensive as a useful tool to end users, monitors etc.
20	Section 9.3.2 Description of Study Assessments<br><br>~~Physical Examination: Physical examination at Baseline Visit will include cardiovascular, dermatological, musculoskeletal, respiratory, gastrointestinal, and neurological systems. Weight~~	Section 9.3.2 Description of Study Assessments<br><br>Weight and Height at Baseline	Section revised to make it more inclusive of all assessments and extraneous wording removed.<br><br>Physical exam was removed as the medical history section records relevant baseline conditions. For this stroke and

	~~will also be recorded. Physical examinations performed at subsequent visits will be at physician discretion.~~		TIA patient population the Modified Rankin and ABCD2 score are the relevant baseline assessments in lieu of the Physical Exam.
	Vital Signs: ~~Vital signs will be assessed for all subjects including blood pressure (BP), temperature (°C), pulse, and respiratory rate. Vital signs will be obtained at Baseline, at each study visit and at the end of study.~~ Resting pulse and blood pressure (BP) measurements will be taken after the subject has been seated for at least five minutes.	**Vital Signs**: Pulse and BP:Resting pulse and blood pressure measurements will be taken after the subject has been seated for at least five minutes at every visit excluding Visit 2 (phone call visit).	Removed temperature and respiration rate as they are not measurements relevant to this study population (stable stroke and TIA patients without active infection or significant medical complications). If there are any significant changes in these measurements the AE, SAE, Outcome Event forms will record this information.
	ECG: It is expected that a 12-lead ECG or cardiac monitoring documented on a printed rhythm strip to exclude atrial fibrillation will have been performed as standard of care for the qualifying stroke/TIA within 28 days of randomisation. This will be reviewed at the Screening ~~Assessment~~ to determine eligibility. Abnormal findings will be noted for clinical significance and signed by an Investigator.	**ECG**: It is expected that a 12-lead ECG or cardiac monitoring documented on a printed rhythm strip to exclude atrial fibrillation will have been performed as standard of care for the qualifying stroke/TIA within 28 days of randomisation. This will be reviewed at Screening to determine eligibility and recorded in the Baseline visit. Abnormal findings will be noted for clinical significance and signed by an Investigator.	Need to emphasise that this is a mandatory exam that needs to be recorded to eCRF.

	~~Clinical Laboratory Tests~~: ~~The following laboratory tests will be reviewed as part of screening. It is expected that they will be performed as standard of care for the qualifying stroke or TIA. Tests within 28 days of randomisation performed at local laboratories will be allowed. The most recent tests performed will be entered on the CRF: Mandatory tests:~~<br>~~- Full blood count: haemoglobin, WBC, platelet count~~<br>~~- Biochemistry: estimated GFR (MDRD or CKD-EPI method), liver function tests~~<br><br>~~All laboratory results will be reviewed by an Investigator who will record the clinical significance of any abnormal results.~~<br><br>~~Concomitant Medication: The subject will be assessed to determine all current habitual over-the-counter or prescription medication, vitamins, and/or herbal supplements. The guidance provided in Section 10.7.2, Prohibited medication and the SPC should be considered if there are any changes to concomitant medication over the course of the study.~~	**Laboratory exams**: see task schedule for visits and windows allowed for all lab tests<br><br>**Concomitant Medication**: The subject will be assessed at Baseline, Visit 6 (104 weeks), Visit 12 (week 260) to determine all concomitant medications that are relevant to this study group:- anti-platelets, anti-coagulants, anti-hypertensives, lipid lowering, occasional anti-inflammatory/immunosuppressant agents, anti-diabetic<br><br>**Concomitant contraindicated medication check**: The subject will be asked at every visit if any medications have been taken that are contraindicated in patients taking colchicine.. (see section 10.7.2)	Needed to simplify this section and refer to the task schedule as the reference point of identifying which tests need to be performed and when.<br><br>The Trial Committee believe that restricting collection of concomitant medications to those that are relevant to this specific trial being performed in the specific sample of Stroke/TIA patient focuses collection and recording of information to that which is pertinent and will be analysed.<br><br>Safety requirement for the patient

	Neurological and Quality of Life Scores: The ABCD2 score (TIA only), modified Rankin score (all patients), Montreal Cognitive Assessment (MOCA), and EuroQO-5L will be recorded (see Appendices 2, 1, 3 &5). The NIH Stroke Scale (Appendix 4) score (stroke patients only) is not mandatory, but if performed results will be recorded.	**Neurological and Quality of Life Scores**: The ABCD2 score (TIA only), modified Rankin score (all patients **- see Appendix 1 for algorithm),** Montreal Cognitive Assessment (MOCA), and EuroQO-5L will be recorded (see Appendices 2, 1, 3 &5). The NIH Stroke Scale (Appendix 4) score (stroke patients only) is not mandatory, but if performed results will be recorded.<br><br>Adverse Event Assessment Monitoring<br><br>Adverse Events:<br>Adverse event collection will begin at randomisation and will finish at the end-of-trial assessment (28 days after last dose of study medication).	Updated section to include the Algorithm information that is useful to the end user at participating sites.<br><br>Moved to include AE info under AE in description of study assessments.
	Outcome event assessment:<br>Subjects will be monitored at each Study Visit for suspected outcome events, which will be managed<br>at each site by local physicians according to the standard of care. Minimum documentation for suspected outcome events will be obtained for central adjudication, the suspected outcome will be reported via the CRF, and a decision relating to withdrawal or continuation of colchicine therapy will be made by the site study physician (for patients randomised to colchicine).	Outcome event assessment:<br>Subjects will be monitored at each Study Visit for suspected outcome events (cardiac events including hospitalization for unstable angina, cardiac arrest, myocardial infarction, stroke/TIA, vascular death) which will be managed at each site by local physicians according to the standard of care. An event form for cardiac event/hospitalization/death/stroke/TIA,MI will be obtained for central adjudication, the suspected outcome will be reported via the CRF, and a decision relating to withdrawal or continuation of colchicine therapy will be made by the site study physician (for patients randomised to colchicine – see section 9.5). Pre-specified supporting documentation will be anonymised, de-identified,	Updated section to include new outcome event of 'hospitalization for unstable angina'.<br><br>Outcome event eCRF forms have been finalized since original protocol was submitted and section needed to be updated with specific details.

		coded, and provided to the Coordinating Centre team, as detailed in Appendix 9.<br><br>**Dispense study medication**: patients randomised to study drug are given 2 packages (100 tabs/pack) at baseline, visit 3 and every subsequent visit. (no drug is dispensed at week 4 visit 1)<br><br>Informed Consent: see screening assessment (see 9.3.3)<br><br>Inclusion/Exclusion criteria: see screening assessment (see 9.3.3)<br><br>Medication Compliance: The number of tablets remaining are counted at every visit excluding the telephone call at day 90 (visit 2)<br><br>**Occurrence of Carotid** Endarterectomy/Stent/Atrial Fibrillation 'since previous visit' assessment Randomisation: see randomisation 9.2.4	All of these were added to Description of study assessment to have one place where the reader can find (or be referred to the appropriate section) information on the assessment.

21	~~9.3.4 Baseline Visit~~<br><br>~~The following assessments will be performed at the Baseline Visit prior to randomisation.~~<br>~~- confirmation of eligibility (review inclusion/exclusion criteria)~~<br>~~- recording of demographics, medical history and concomitant medications~~<br>~~- physical examination, including weight~~<br>~~- vital signs~~<br>~~- results of ECGs/rhythm strips, laboratory (biochemistry and haematology tests), and investigations for qualifying TIA/stroke aetiology will be recorded.~~<br>~~- Modified Rankin score (Appendix 1), and ABCD2 score (TIA patients only) (Appendix 2) will be recorded~~<br>~~- MOCA (Appendix 3) and EuroQOL (Appendix 5) assessments will also be performed~~<br><br>~~Randomisation will be performed using the IWRS/IVRS system. If randomised to colchicine, study medication will be provided. If randomised to Usual Care, medications for usual care (anti-thrombotic, lipid-lowering, anti-hypertensive) will be recorded on the CRF. Advice on Lifestyle~~	Section 9.3.4 Baseline visit removed and updated with the following:<br><br>**9.3.4 Summary of Assessments at visits, End of Study/Withdrawal Visit, Investigator support:**<br>**a) Assessments at all visits are:**<br>Safety – adverse events, pulse blood pressure Modified Rankin Score<br>Outcome Events<br>Compliance Study Medication<br>Contraindicated Concomitant Medications<br>Dispense Study Drug<br><br>**b) Additional** assessments particular to specific visits are:<br>CRP, B12: at week 4,<br>weeks 52, 104, 156, 208, 260<br>Hgb, WBC, Platelets, ALT, AST, eGFR, Creatinine: weeks 52, 104, 156, 208, 260 EuroQol-5L, MOCA, Relevant Concomitant medication weeks 104, 260	This section has been simplified to make it more user friendly to staff at study sites. The task schedule outlines all the assessments required at each visit. This section was simplified and updated to give a general summary of assessments across the study.

	(smoking cessation, diet and physical activity) will be provided to all patients. The subject will be deemed to have entered the trial at the point of randomisation.		
22	9.3.5 Subsequent Study Visits and End-of-Trial/Withdrawal Visit:<br>Clinic visits will be performed at 4 weeks, 6 months, and every 6 months thereafter until an outcome event occurs or end of trial (maximum duration 60 months in patients entered in the Vanguard Stage, Year 1).<br>At each visit, the following will be assessed:<br>- assessment for outcome events<br>- modified Rankin score<br>- assessments of safety (assessment for adverse events, vital signs, physical examination at physician discretion)<br>- laboratory assessments: CRP, and B12 will be measured by local laboratories at 4 weeks (Study Visit 2) and at annual intervals (Study Visits 4,6,8,10 and End-of-Trial). At each annual visit (Study Visits 4,6,8,10 and End-of-Trial), a serum creatinine, eGFR, LFTs, and full blood count taken within the previous 60 days will be recorded. If taken for clinical care, this will be allowed. If this has not been performed for clinical care, blood will be taken at the study visit for creatinine/eGFR/FBC/LFT measurement at local hospital laboratories.<br>- dispensing of study medications<br>- recording of concomitant medications	Current section 9.3.4 (per item 21 above) replaces this section	Compacted 9.3.4 and 9.3.5 into one section.

	- assessment of compliance with study medications<br><br>A telephone and email helpline/support service will be available for study investigators for questions which may arise at follow-up Study Visits. This service will be operated by the Trial Coordinating Centre and national Lead Investigators.<br>***A brief telephone assessment will be performed at 90 days:***<br>At this call, performed by the Site study staff, the following will be performed:<br>- assessment of early outcome events<br>- assessments of safety (interview for signs and symptoms suggesting adverse events)<br>- recording of concomitant medications<br>- assessment of compliance with study medications<br><br>***End of trial/Subject Withdrawal visit:***<br>At EOT/Subject Withdrawal visit, the following will be assessed:<br>- suspected outcome events<br>- modified Rankin score<br>- assessment for adverse events, vital signs, physical examination at physician discretion<br>- laboratory assessments as detailed above<br>- recording concomitant medications<br>- compliance assessment with study medications<br>- MOCA (Appendix 3)<br>- EuroQOL-5L (Appendix 5)		

23	Section 10.4 Accountability of Study Colchicine<br><br>In practice, when a patient is randomised at a site, the patient will be allocated to active treatment or usual care. If randomised to study medication~~, the Site Investigator will select the next available package of study medication from the available kits at the study site. The investigator/research nurse will collect the medication from the pharmacy, as applicable, and instruct the patient in writing and verbally when to begin study medication (typically this will begin the morning after randomisation).~~	This section was reworded and moved to Randomisation Section 9.2.4, as follows (4th para added)<br>In practice, when a patient is randomised , the patient will be allocated to active treatment, or, usual care. If randomised to study medication, the patient will be given one dose from the next available package of study medication at the study site. This package (minus the first dose) and a second package will be given to the patient to take home.The patient will be advised to take the medication once daily and in the morning. If the dose is forgotten in the morning it can be taken at a later time in the day. The patient will also be instructed never to take more than one tablet per day even if a previous day/days dosage has been missed	Reworded and moved to a more appropriate location, to simplify the protocol text.
24	Section 10.7 Prior and Concomitant Therapy While on ~~colchicine or usual care medications, information on any other medication taken during the study will be collected and recorded in the CRF at the Baseline Visit, and each subsequent Study Visit and telephone call to end of the trial.~~	Section 10.7 updated as follows:<br>While on colchicine or usual care information on colchicine contraindicated medications (see 10.7.2) will be taken during each study visit.<br><br>Medications relevant to this patient population will be collected at baseline, week 104, week 260 (anti-platelet, anti-coagulant, anti-hypertensive, lipid lowering, occasional anti-inflammatory/immunosuppressant agents)	Reworded only.<br><br>The Trial Committee believe that restricting collection of concomitant medications to those that are relevant to this specific trial being performed in the specific sample of Stroke/TIA patient focuses collection and recording of information to that which is pertinent and will be analysed.

25	Section 10.7.2 ~~Prohibited Medications~~<br><br>Subjects assigned to the colchicine arm will be given a wallet card listing prohibited medications during the trial. If a prohibited medication is required during the study, then colchicine will be interrupted ~~for the duration of therapy of the prohibited medication and re-started after an appropriate wash-out period (minimum 7 days or at least 5 half-lives, whichever is longer) after the last dose of the prohibited medication is taken.~~	Section 10.7.2<br><br><u>Contra-indicated prohibited medications</u><br>Subjects assigned to the colchicine arm will be given a wallet card listing contra-indicated (prohibited) medications during the trial. If a prohibited medication is required during the study, then colchicine will be interrupted <u>as outlined in section 9.5.2. The GP letter will also include the list of contraindicated medication.</u>	Updated as the original protocol wallet card did not include the prohibited meds while on Colchicine.
26	Appendix 1: Modified Rankin Scale	Appendix 1: Modified Rankin Scale<br><u>STANDARDISED mRS Algorithm</u>	Algorithm was added
27	Appendix 2: ABCD2 Score<br>Appendix 3: Montreal Cognitive Assessment (MOCA)<br>Appendix 4: NIH Stroke Scale<br>Appendix 5: EQ5D-5L<br>Appendix 7: Expected Adverse Events after Stroke<br>Appendix 8: Expected Adverse Events after Colchicine<br>Appendix 9: Minimum Source Documents for Outcome Adjudication<br>Appendix 10: Creatinine Clearance by MDRD Formula<br>Appendix 11: Creatinine Clearance by CKD-EPI Formula	Reformatted Appendices to a Word insert format from 'table' and 'object' format previously	To facilitate easier updates to protocol if required in the future
28	Appendix 6: Universal Definition of Myocardial Infarction	Appendix 6: Universal Definition of Myocardial Infarction<br><u>TIMI Definition for Hospitalization of Unstable Angina</u>	Hospitalization for Unstable Angina has been added as an outcome event and this definition needed to define the event.

29	Objectives: Key exclusion criteria #6<br>Impaired hepatic function (transaminases greater than twice upper limit of normal)	Updated to:-<br>Impaired hepatic function (transaminases ALT and/or AST greater than twice upper limit of normal)	AST is not performed in all hospitals for assessing hepatic function
30	Safety pg. 16 para 2:<br>Per the FDA SPC, excessive accumulation or overdose of colchicine has been associated with a range of other adverse events, which are 'generally reversible upon temporarily interrupting treatment or lowering the dose'. These include: neuropathy, alopecia, rash, myelosuppression, elevated transaminases, myopathy, muscle pain,	Safety: para 2:<br>Per the FDA SPC, excessive accumulation or overdose of colchicine has been associated with a range of other adverse events, which are 'generally reversible upon temporarily interrupting treatment or lowering the dose'. These include: neuropathy, alopecia, rash, myelosuppression, elevated transaminases (ALT and/or AST)., myopathy, muscle pain,	AST is not used in all hospitals for assessing hepatic function
31	9.2.5 Investigations prior to enrolment in the trial<br>3. Liver transaminases (AST and ALT)	Update to<br>3. Liver transaminases (ALT and/or AST)	AST is not used in all hospitals for assessing hepatic function
32	Task Flow Schedule	Updated to<br>ALT and/or AST in schedule<br>Removed the 'mandatory' footnote from the non-mandatory 'Lipids, CK, CRP…)<br>And<br>'Other Stroke/TIA tests…'	AST is not used in all hospitals for assessing hepatic function
33	9.3.2 Adverse Events<br>5. Hepatic (transaminases ≥2 ULN)	9.3.2 Adverse Events<br>5.Hepatic (transaminases ALT and/or ≥2 ULN)	AST is not used in all hospital for assessing hepatic function
34	9.3.4 Summary of Assessments at visits<br>Hgb, WBC, Platelets, ALT, AST eGFR, Creatinine: weeks 52, 104, 156, 208, 260	Updated to<br>Hgb, WBC, Platelets, ALT and/or AST eGFR, Creatinine: weeks 52, 104, 156, 208, 260	AST is not used in all hospital for assessing hepatic function

35		Page 31: Section End of trial/Subject withdrawal visit:<br><br>The following text has been added: Patients who withdraw from the trial or for whom trial treatment is terminated will not be replaced.	Compliance with Canadian regulations
36		Page 51:<br>Section 15: Retention of Essential Documents:<br>The following text has been added:In Canada only records will be retained and maintained for 25 years, as per section C.05.012 of Canadian Food and Drug Regulations	Compliance with Canadian regulations
37.	**4. Synopsis**<br>Key Exclusion Criteria Item 15 pg9-10<br>Pregnancy, breast-feeding, or pre-menopausal woman<br><br>**9.2 Selection of study population**<br>9.2.3 Exclusion Criteria Item 15 pg25	**The following text has been added:**<br>Women of childbearing potential (WCBP), or pregnant or are breastfeeding, are not eligible to participate in this study. A woman of childbearing potential is a woman who:<br>•has not had surgery to remove the uterus and ovaries<br>•has had menstrual periods at any time in the preceding 24 consecutive months<br>•Menstrual periods interrupted due to cancer chemotherapy treatment are considered WCBP as this may still allow conception.<br><br>Pregnancy is considered highly unlikely during the trial because women of childbearing potential are excluded. However, in the unlikely event that a woman in the trial becomes pregnant, pregnancy information will be collected.	Clarification for The Office of Clinical Trials, Canada
38	Section 9.3.2: Outcome event assessment (page 30)<br><br>Text: Pre-specified supporting documentation will be anonymised, de-	Changed to:<br><br>Pre-specified supporting documentation will be pseudo-anonymised (the only reference to the	Compliance with Ethics Committee request, Belgium

	identified, coded and provided to the Coordinating Centre team, as detailed in Appendix 9	patient will be their CONVINCE Subject ID number) and provided to the Coordinating Centre team, as detailed in Appendix 9	
39	Section 15: Retention of essential documents (page 51)	Text added:<br><br>'In Belgium records also need to be retained for 25 years after the end of the study'	Compliance with Belgian regulations
40	Section 18.6: Subject confidentiality (Page 53)<br><br>Patient data will be subject to rigorous procedures to ensure data protection, as outlined in detail in the data protection section of this application. Patient information will be de-identified and anonymised, and no patient will be identified in any public presentation of material related to the trial.	Changed to:<br><br>Patient data will be subject to rigorous procedures to ensure data protection, as outlined in detail in the data protection section of this application. Patient information will be pseudo-anonymised, and no patient will be identified in any public presentation of material related to the trial.	Compliance with Ethics Committee request, Belgium
41	Section 12.5<br><br>Removed: No hypothesis testing comparing the primary outcome between arms will be performed on interim analyses. Therefore, no correction for multiple statistical comparisons will be required.	Changed to:<br><br>Appropriate correction for multiple statistical comparisons for interim analyses will be done.	Request by SwissEthics regarding inconsistency in protocol
42	Section 15. Retention of essential documents (page 51)<br><br>Removed '5 years'	Added:<br>In Switzerland, in accordance with Article 45 ClinO, data will be retained for 10 years after the conclusion or revocation of the clinical experiment.	Request by SwissEthics
43	Section 9.3.2: Laboratory Exams, page 30:	Text added:<br><br>'All blood samples collected for safety monitoring will	Request by SwissEthics

		be measured by local laboratories and destroyed by local laboratories according to their standard procedures. The protocol does not require any special storage or processing of these samples.’	
44	Section 11.13: New section added: Heading ‘Switzerland only, New investigations for safety monitoring’	Text added:<br>An annual safety report shall be submitted once a year to the relevant Ethics Committee and to SwissMedic. If security or safety measurements must be taken directly while a clinical trial is being carried out, the examiner must inform the ethical review committee which measurements were taken and the circumstances under which they were necessary, within 7 days (Clinical trials article 37).	Request by SwissEthics
45	Section 18.6: Subject Confidentiality	Text added:<br><br>In Switzerland, birth year only will be entered	Request by SwissEthics
46	Section 15: Retention of Essential Documents	Added:<br>In the event of withdrawal, the data collected up to that point will still be evaluated in encrypted form, otherwise the entire project will lose its value. After the evaluation, the data is completely anonymized, i.e. the key assignment is destroyed, so that no one can know who originally provided the data.	Request by SwissEthics
47	Section 1.5	Added:<br><br>CONVINCE is a clinical trial of medicinal products under category C (colchicine is not authorised in Switzerland)	Request by SwissEthics
48	Section 11.6: Reporting procedures for Serious Adverse Events	Added:<br><br>Switzerland only: In accordance with Art 40 ClinO, SAEs resulting in death are reported to the Ethics	Request by SwissEthics

		Committee via BASEC within 7 days	
49	Section 11.6: Reporting procedures for Suspect Unexpected Serious Adverse Reactions (SUSAR)	Added:<br>Switzerland only: In accordance with Art 41 ClinO, the investigator shall notify the responsible Ethics Committee via BASEC of a fatal SUSAR occurring in Switzerland within 7 days, and of any other SUSAR within 15 days. These notifications shall also be made to the competent regulatory authority (SwissMedic). This obligation rests on the sponsor.	Request by SwissEthics
50	Section 18.3, page 53:<br>Approvals, Regulatory and Ethics Compliance:	Added:<br>**Switzerland only:** In accordance with Art 38 ClinO, the completion of the study will be reported by the local site investigator to the relevant ethics committee within a period of 90 days. Premature discontinuation or interruption of the study will be reported by the local site investigator to the relevant ethics committee within 15 days and will include an explanation of the circumstances. In addition, a final report will be submitted to the Ethics Committee within one year of completion or discontinuation of the study. Reporting duties and timelines are the same as for the relevant Ethics Committee, except of non-substantial amendments that shall be reported as soon as possible. These obligations rest on the sponsor.	Request by SwissEthics
51	**Protocol Synopsis, page 10:**<br>2623 deleted	3154 inserted	Revision of sample size by trial statisticians

52	**Sample size estimation, Section 12, deleted.**<br>The CONVINCE trial is designed to detect a 25% risk reduction at 3-years from 18.0% to 13.5% with a power of 80% and 5% two-sided significance. To allow for 20% dropouts we have inflated the size of the treatment group by 25%. For the statistical estimation of sample size, uniform recruitment over a two year period has been assumed, with two years further follow-up, consistent with the design of the Full Trial stage. (The additional 12-month follow-up period for the approximately 265 patients recruited in the Vanguard stage will provide a modest further increase in statistical power). The primary analysis uses the Intention to Treat (ITT) principle analysing all subjects including a drop-out rate of 20% in the colchicine arm. It is assumed that the treatment effect in the 20% dropouts will be half the treatment effect of those who remain on colchicine for the duration of the trial, with a consequent event rate of 15.75% among patients who drop out [ie. (13.5+18)/2]. The overall colchicine-treated group, including the dropouts, will then have a 3-year event rate of 13.95% (0.8x13.5% + 0.2x15.75%). Therefore, the study needs to be powered to detect the 18.0% versus 13.95% difference. This gives a sample size requirement of 1,457 subjects in the colchicine- treated group and 1,166 in the usual care group (total 2,623	**Inserted text:**<br><br>The original sample size for CONVINCE was 2,623 patients. However, data from recent clinical trials and registries indicate that the rate of stroke/cardiac recurrent events may have reduced since the initial sample-size calculation. In FOURIER, the annualized major cardiovascular event (MACE) rate in statin-treated stroke patients was 3.8%, in PROFESS it was 5.2%, and the 1-year MACE rates in NAVIGATE-ESUS were 5.4% and in TIARegistry.org was 6.2%. The original estimate of the annualized MACE rate in the control arm of CONVINCE was 6%/year, or 18% over a median 3-year follow-up period. Based on these more recent data, as a precautionary measure to maximise statistical power, a protocol amendment has been made to increase the trial sample size based on a more conservative estimate annualized rate of our composite outcome in controls of 4.5%/year (13.5% over median 3-year follow-up).<br><br>Assuming a 25% effect size in colchicine-treated patients after adjusting for a 15% non-compliance rate (relative hazard 0.75, alpha 0.05, power 80%), the revised sample size is increased to 3,154 patients (see Appendix 13). This may over-estimate the required sample size, as the annualized estimated outcome rates are deliberately conservative, and some trials have reported higher annualized rates (above). The estimated effect size of 25% is also deliberately conservative - in the Australian LoDoCo trial, the effect size associated with colchicine was 67% (hazard ratio 0.33, 95% CI 0.18–0.59). However, the sample size is deliberately	Revision of sample size by trial statisticians

	subjects). This sample size is justified as follows: The risk of recurrent stroke after first stroke or TIA is substantial, particularly in the first 28 days. The CONVINCE study protocol allows recruitment of patients as early as 72 hours of stroke onset, thus including patients when early recurrent stroke risk is highest. In 840 Dublin patients with minor stroke/TIA treated with modern stroke prevention medications, the 90-day risk of recurrent stroke was 4.95% and in 3 Harvard cohorts it was in the range 5.3-6%. The German Stroke Units Collaboration reported recurrent stroke rates of 4.8%/year (95% CI 3.9-6.2%). In patients recruited a median of 3 months after index stroke, the SPARCL trial reported annual recurrent ischaemic stroke rates of 2.24% (atorvastatin 80mg arm) and 2.64% (placebo-treated). In the SPS3 trial, the rate of recurrent stroke was 2.5-2.7% per year. In patients recruited within 90 days of index stroke, MATCH found recurrent stroke rates of at 18 months of 8% (aspirin+clopidogrel arm) and 9% (aspirin- alone). A recent systematic review reported pooled recurrent stroke risks of 11.1% (1-year) and 26.4% (5-year)·Based on this data, we conservatively estimate a recurrent stroke rate of 5.5% in Year 1, and 2.5% in subsequent years in patients receiving Usual Care. This equates to a cumulative rate of 10.5% over a 3-year median follow-up period. In a systematic	based on conservative estimates to reduce the risk of inadequate power.	

	review (39 studies, 65,996 patients), Touze reported a consistent 2.1%/year rate of non-stroke vascular death and 0.9%/year nonfatal MI in stroke survivors (total 3%/year)[9]. We conservatively estimate 2.5%/year combined for non-fatal MI, and vascular death (cumulative rate 7.5% over 3-year follow-up). Together, a conservative estimate of the 3-year rate of our primary composite endpoint outcome therefore is 18% in patients randomised to Usual Care.Similar to SPARCL, POINT, and other secondary prevention trials in stroke, CONVINCE is powered to detect a clinically-important relative reduction (effect size) of 25% in the rate of the primary endpoint in colchicine-treated patients (power 80%, 2-sided alpha 0.05), equivalent to absolute rate of the primary composite outcome of 13.95% at 3 years (number-needed-to-treat 20).		
53	**New Appendix 13 added**		Detailed re-estimation of sample size
54	Protocol synopsis page 11<br>Section 6.2 Description of Colchicine and Rationale for the study page 16<br><br>Section 9.1 Trial design, General considerations page 22	Inserted 24-84 months	Recruitment pause and reduced recruitment due to COVID-19 requires extension of recruitment period, with extended duration of treatment for patients already in the trial

	Duration of treatment deleted 12-60 months		
55	**Section 9.2.2, page 23,**<br>**Inclusion criteria**	**Added to criterion 3:**<br><br>**Retinal infarction due to retinal artery occlusion<br><br>is allowed	Response to investigator queries. Clarification of inclusion criteria
56	**Protocol synopsis, Exclusion criteria page 10**<br><br>**Section 9.2.2, page 23, Exclusion criteria**	**Added to criterion 7**<br><br>**Tolbutamide**	Response to investigator queries. Clarification that concomitant use of tolbutamide is an exclusion criterion for trial entry due to potential CYP3A4 inhibition
57	**Section 11.2.3**<br>**Serious adverse events**	***ADDED TEXT:***<br>*Hospitalisaton is defined as overnight stay in hospital. The following are not defined as hospitalisation and do not need to be reported as SAEs: Visits to clinics or day hospitals, Emergency Departments, or other hospital day services (eg. minor surgical procedures); surgeries or procedures planned before randomisation but conducted after; hospital admission for an outcome event.*<br><br>*Note that some events associated with brief treatment in hospital may not meet the definition of hospitalisation, but may be reportable as 'important medical events in the opinion of the investigator' if they pose a risk to life or disability.*	Response to investigator queries.<br><br>Clarification of definition of hospitalisation for SAE reporting

58	**Section 11.4: Reporting procedure, all adverse events**<br><br>**Section 11.6: Reporting procedure for Serious Adverse Events**	**Added text:**<br><br>In addition, SAEs will be reported within 24 hours to the pharmacovigilance office using the SAE Reporting Form (available in the site file), by emailing sae.reporting@ucd.ie	Response to investigator queries.<br><br>Clarification of process for SAE reporting
59	**New section: 11.4.1 Targeted reporting of adverse events: page 44**	Certain adverse events are reported more frequently in patients with stroke or treated with colchicine. Adverse events in the following categories should be reported as standard, regardless of study arm:<br>Bone marrow depression<br>New peripheral neuropathy<br>Abdominal pain, nausea, vomiting, diarrhoea<br>Hepatic injury<br>Myopathy, myalgias, rhabdomyolysis<br>New acute or chronic renal injury<br>Oligospermia or azoospermia<br>Low serum B12<br>Infections<br>New malignancy (cancer)<br>Major haemorrhage<br>Definitions for each category are provided in Appendix 14.Other events may be reported if meeting the definition of adverse events in the opinion of the site investigator.	Response to investigator queries. Clarification of adverse events for standard reporting
60	**New appendix 14**	**Definitions of targeted adverse events**	Response to investigator queries. Clarification of definitions of adverse events for standard reporting

61	**Section 9.5.1: Discontinuation of colchicine**	**Text added:**<br><br>An interruption of colchicine for 6 months or greater is classified as permanent discontinuation.<br><br>The eCRF is continued as per the existing visit schedule. An End of Trial/Early Withdrawal form is not completed if the patient remains in follow up, even if colchicine is prematurely discontinued.	Response to investigator queries. Clarification of procedures regarding colchicine discontinuation
62	**Section 9.2.4: Randomisation**<br><br>**Deleted text: subjects will be** given one dose from the next available package of study medication at the study site. This package (minus the first dose) and a second package will be given to the patient to take home.	Changed to:<br><br>instructed to take the first dose on the day of receipt of study medication, with food.	
63	**Section 9.5.2: Management of colchicine interruption**	**Added text:**<br><br>*Note: Non-steroidal anti-inflammatory drugs (NSAIDs) and steroids are not moderate/strong CYP3A4 or P-gp inhibitors. No colchicine interruption is required if a short course of these medications is required (3 weeks or less)*	Response to investigator queries. Clarification of procedures regarding co-treatment with NSAIDs or steroids
64	**Section 11.12: Outcome reporting and blinded outcome adjudicaton**	**Added:**<br><br>*Note: If site team think a patient probably had an outcome, but it is still not fully clear that a definite outcome has occurred, then please email the trial project office within 24 hours (isctn@ucd.ie). The*	Response to investigator queries. Clarification of procedures regarding reporting if an outcome is probable but not definite

		team will help with monitoring this patient until it is clear that an outcome has occurred, or else the event may need to be re-classified and reported without delay as an SAE.	
65	**New Appendix 15**<br><br>**Modified trial procedures in response to COVID-19**		Modification of trial procedures in response to COVID-19 pandemic
66	**New text, 9.3.3a**<br><br>**Protocol update: Recruitment option for patients discharged early on COVID-19 pathway**	**Added:**<br>Most patients will be recruited as outlined above. ***This remains the preferred option.*** Some patients meet all other eligibility criteria but are rapidly discharged from hospital according to new COVID-19 pathways before 72 hours have passed since the Qualifying Event (stroke or high-risk TIA).<br><br>In this situation, as an alternative option, the following procedure may be applied:<br>1. informed consent is obtained from the patient before hospital discharge. The Screening/Baseline visit may be done at this time, before hospital discharge or may be done by phone after discharge.<br>2. As soon as possible after the 72 hour period has passed, the patient should be contacted by phone<br>3. The site team must verify that the patient is still eligible for the trial. If the Screening/Baseline visit has not been completed before discharge, it can be completed during the phone call.<br>4. Randomisation is done during the phone call	Modification of trial procedures in response to COVID-19 pandemic

		5. If randomised to colchicine, study drug is provided to the patient as outlined below and the usual instructions provided for storing and taking colchicine<br>6. The Screening/Baseline Form in the eCRF is completed as usual.<br>**7.** The MOCA need not be done. This is not classified as a Protocol Violation. The EQ5D may be done by phone (it is validated for phone use)	
67	**New text, Section 9.3.1a**<br><br>**Protocol update: Schedule of visits:**	**Added:**<br>In response to the COVID-19 pandemic, to reduce unnecessary visits to hospital, the following procedures will apply:<br><br>1 **<u>Timing of follow-up visits</u>**: All follow up visits should be completed as close as possible to the schedule outlined in the study protocol. Increased flexibility is permitted for the timing of the follow-up visit, if judged by the site team that this is a practical solution to completing the visit. If the visit is conducted outside the range +/-7 days of the scheduled date, this is not classified as a Protocol Violation.<br><br>2 The protocol currently allows for follow-up visits to be completed by telephone with patient supported by a family member if needed. **Remote visits may also be done by video-call** (using Skype, Zoom, or other applications).	Modification of trial procedures in response to COVID-19 pandemic
68	**New text, Section 9.3.2**<br><br>**Protocol update: Description of study assessments**	**Added:**<br><br>**New text updating procedures for medication compliance, pulse and blood pressure measures, neurological scores, and laboratory tests, in response to COVID-19 pandemic.**	Modification of trial procedures in response to COVID-19 pandemic

69	**New text, Section 10.8**<br><br>**Protocol update: Colchicine dispensing**	**Added:**<br><br>**New text updating procedures for colchicine dispensing, in response to COVID-19 pandemic.**	Modification of trial procedures in response to COVID-19 pandemic
70	**New text, Section 11.6**<br><br>**Protocol update: Safety reporting of COVID-19 cases as SAEs**	**Added:**<br><br>**New text updating procedures for SAE reporting, to instruct investigators to report all COVID cases as SAEs, in response to COVID-19 pandemic.**	Modification of trial procedures in response to COVID-19 pandemic

Appendix 13

Re-estimation of Sample Size for CONVINCE Study (v2.1 6th June 2018)

Cathal Walsh
Leslie Daly

Contents

Introduction **121**

Hazards and person years of follow-up **124**

Hazards **124**

The effect of dropouts from the treatment arm 125

Annual hazard rate in ITT treatment group 125

Number of events required to detect a relative hazard of 0.746 126

Person-years of follow-up **127**

Years of follow-up to end March 2018 127

Years of follow-up from the start of April 2018 to the end of July 2019 127

Years of follow-up from the start of August 2019 to the end of June 2020 127

Follow-up from start July 2020 to end September 2022 128

Events **128**

Number of events up to end September 2022 128

Adjustment for reduced person-years of follow-up in those who had an event 129

Conclusion and discussion **129**

Introduction

This sample size re-estimation was conducted according to the methodology described by Prof Leslie Daly in his calculations in Version 2 dated April 24th 2018. This calculation assumes a rate of 4.5% of the composite outcome in controls, and a 26% effect size, before accounting for non-adherence to study drug. This version includes patients recruited up to the end of March 2018, close to completion of the Vanguard phase of the study. By this stage 220 patients had been randomized. Table 1 shows what month these 220 patients were recruited from December 2016 when the first patient was entered into the study. Table 2 shows the projected patient entry into the trial up to the end of June 2020. The study design requires a further follow-up of all patients to the end of September 2022.

The sample size calculation is based on the information contained in these two tables. This is a technical report created to provide a comprehensible, checkable description of the sample size calculation.

The aim of CONVINCE is to detect a 26% reduction in the 3-year event rate from 13.5% to 9.99%, at a 5% two-sided level of significance with 80% power.

The outcome of the sample size calculation is that the study requires 3,154 patients projected to be recruited by the end of June 2020, together with a further two years follow-up of all 3,154.

Table 1 Accrual into trial up to end March 2018

**Month**	**Number of Patients**	Months of follow-up	**Person-years of follow-up**
**Dec-16**	**1**	15.5	**1.29**
**Jan-17**	**2**	14.5	**2.42**
**Feb-17**	**5**	13.5	**5.63**
**Mar-17**	**8**	12.5	**8.33**
**Apr-17**	**12**	11.5	**11.50**
**May-17**	**13**	10.5	**11.38**
**Jun-17**	**20**	9.5	**15.83**
**Jul-17**	**19**	8.5	**13.46**
**Aug-17**	**12**	7.5	**7.50**
**Sep-17**	**7**	6.5	**3.79**
**Oct-17**	**14**	5.5	**6.42**
**Nov-17**	**8**	4.5	**3.00**
**Dec-17**	**22**	3.5	**6.42**
**Jan-18**	**18**	2.5	**3.75**
**Feb-18**	**20**	1.5	**2.50**
**Mar-18**	**39**	0.5	**1.63**
**Total**	**220**		**104.83**

Table 2 Projected accrual into trial up to June 2020

Year	Month	Projected cumulative total at end of month	Projected recruitment in month	Months of follow-up to end July 2019	Person years of follow-up
2018	April	250	30	15.5	38.75
	May	293	43	14.5	51.96
	June	346	53	13.5	59.63
	July	409	63	12.5	65.63
	August	479	70	11.5	67.08
	September	556	77	10.5	67.38
	October	640	84	9.5	66.50
	November	731	91	8.5	64.46
	December	829	98	7.5	61.25
2019	January	934	105	6.5	56.88
	February	1044	110	5.5	50.42
	March	1159	115	4.5	43.13
	April	1279	120	3.5	35.00
	May	1404	125	2.5	26.04
	June	1534	130	1.5	16.25
	July	1669	135	0.5	5.63
	August	1804	135		
	September	1939	135		
	October	2074	135		
	November	2209	135		
	December	2344	135		
2020	January	2479	135		
	February	2614	135		
	March	2749	135		
	April	2884	135		
	May	3019	135		
	June	3154	135		
	**Total person years (up to end July 2018)**				**775.96**

Hazards and person years of follow-up

Because of the complex study implementation, which had no defined accrual period with uniform patient entry, the more standard survival study sample size approaches were not applicable. Instead this calculation is based on determining the number of events (endpoints) that must be observed in the trial to achieve the desired level of power and significance to detect a given treatment effect. It is then possible to convert the number of required events to the number of patients needed. The concepts of a hazard and a person-year of follow-up are central to the estimation and hazards form the input to the sample size method that is at the core of this calculation.

An annual hazard rate (also referred to without the 'rate' or 'annual' qualifiers as a plain hazard) is very similar to an annual event risk. For instance a one-year event risk of 5% corresponds to an annual hazard rate of 0.05129[1]. A three year risk of 13.5% translates to an annual hazard of 0.04834. Allied to the concept of a hazard is that of person-years of follow-up. A person-year of follow-up is one person followed for one year, and no distinction is made between, for example, two persons followed for 10 years each and four persons followed for five years – each set of persons having 20 person-years of follow-up. Person-years of follow-up can be added together for different individuals.

Assuming the hazard rate is constant over time, the hazard rate multiplied by the actual person- years of follow-up yields the number of events[2]. If two different groups are combined the hazard for the total group is a weighted average of the hazards in the groups with the weights defined by the person-years in each group.

An alternate viewpoint is that annual hazards, multiplied by 1,000, give the number of events that would be observed with 1,000 person-years of follow-up. Thus a hazard of 0.05129 would give 51.29 events.

The main issues in this sample size calculation were then determining the appropriate hazards and person-years of follow-up in the trial.

Hazards

The trial specifications were originally in terms of risks. The trial was designed to detect a 26% reduction in the three-year event risk from 13.5% assumed in controls to 9.99% - corresponding to a relative risk of 0.74. **A 13.5% 3-year risk equates to an annual hazard rate of 0.04834 and the 9.99% risk is equivalent to an annual hazard of 0.03508.** This is a relative hazard of 0.03508/0.04834 = 0.726.

---

[1] An annual hazard *h* can be calculated from a *t*-year mortality risk of M(*t*) using

$$h = \frac{-(1 - M(t))}{t}$$

[2] This formulation is a crude approximation and ignores the distinctions between continuous and discreet
time. Additionally any fatal events that occur actually reduce the person-years of follow-up. The follow-up quantities being dealt with are essentially potential person-years of follow-up, though the hazard should only be applied to person years at risk of an event – i.e. follow-up periods after a death should be excluded. A heuristic adjustment is made for this.

The effect of dropouts from the treatment arm

The primary endpoint of this trial is determined based on an ITT analysis. Dropouts in controls have no effect on the analysis or sample size calculation since dropouts are followed up anyway. In the treatment group however dropouts will cease taking their trial medication and while they may experience a treatment benefit before their withdrawal, subsequently, apart from perhaps a short carry over effect, will not experience any of the treatment benefits. The treatment group dropouts should revert to the control event rate after withdrawal.

With an ITT analysis the event rate in the treatment group will be therefore greater than that stipulated in a pure treatment group with no dropouts.

The estimation of this effect, that has been used here, is based on an assumption that on average dropouts occurred halfway through their total follow-up. This is reasonable since there might be early dropouts due to aspects of the treatment which were unpalatable, but dropouts later in the trial due to being fed up with the regular follow-up. Under this assumption half the total person- years in dropouts would be associated with the lower treatment hazard rate and half with the higher rate experienced by controls. Because of the equal number of person-years with each hazard rate the total hazard rate experienced by the dropouts is a simple average of the two rates involved – the rate of 0.04834 specified for controls and the rate of 0.03508 in the group who received treatment.

Hazard in treatment dropouts = (0.04834 + 0.03508)/2 =-

0.04171 This corresponds to a 3-year risk of 11.8%

Annual hazard rate in ITT treatment group

It is assumed for this sample size calculation that there is a total 15% dropouts in the treatment group. Because these dropouts have a higher hazard rate (0.04171) than the non-dropouts (0.03508) the annual hazard rate in the treatment group is increased to a weighted average of the two rates weighted according to the proportion of dropouts. Allowing for dropouts

Hazard in ITT treatment group = (0 .15 x 0.04171 + 0.85 x 0.03508) = 0.03608

This hazard corresponds to a three year event risk of 10.3% in a treatment group with 15% dropouts compared to 9.99% in a treatment group without dropouts[3]. Consequently for an ITT analysis the study must be sufficiently powered to detect the smaller difference in hazards, 0.04834 in controls versus 0.03608 in the treatment group instead of 0.03508 (the original treatment hazard)– corresponding to a relative hazard of 0.746 rather than the 0.726 originally specified. (see Table 3)

[3] To convert an annual hazard *h* to a *t*-year mortality risk M(*t*) the following holds:

$$(t) = 1 - e^{-ht}$$

Table 3 Hazards and 3-year event rates

Group	Annual hazard rate	3-year event risk	Relative hazards	Relative Risks
Control group	0.04834	13.5%	1.00	1.00
Treatment group (no dropouts)	0.03508	9.99%	0.726	0.74
Dropouts (in treatment group)	0.04171	11.8%	-	-
Treatment group (15% dropouts)	0.03608	10.3%	0.746	0.76

Number of events required to detect a relative hazard of 0.746

The underlying sample size calculation is based on the assumption that the analysis will use Cox's proportional hazard regression to adjust for confounders, and that the trial involves a uniform entry rate in an accrual period with a further follow-up. The method is described by Schoenfeld (1983)[4]. A web-based implementation of the approach was employed (http://www.sample-size.net/sample- size-survival-analysis/). Though the accrual for this trial is not uniform it is the required number of events that is critical.

To detect a relative hazard of 0.746 (corresponding to a 3-year event rate of 13.5% in controls and 10.3% in the treatment group) at a 5% two-sided level of significance with 80% power requires a total of 367 events in the treatment and control groups combined. (Figure 1)

Figure 1 Output from sample size program.

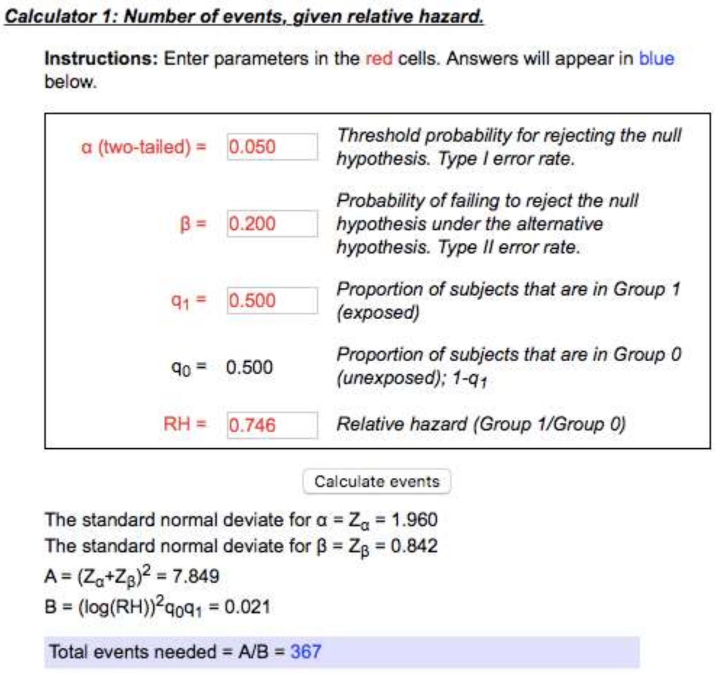

[4] Schoenfeld D (1983). Sample size formula for the proportional-hazards regression model. Biometrics 39:499- 503.

Person-years of follow-up

This section relates to determining the expected potential person-years of follow-up in the study up to the anticipated end of recruitment at the end of June 2020 and in the subsequent 27 months of follow-up to the end of September 2022.

Years of follow-up to end March 2018

Table 1 shows the month of entry of the 220 patients randomized by the end of March 2018. The average months of follow-up to this date are also shown in column 3. Thus the two patients, for example, who entered the trial in January 2017 each had on average 14.5 months of (potential) follow-up to end of March in the following year. The person-years of follow-up for all patients entered in a given month are shown in column 4. For example the above two patients had

- A total of 2 x 14.5 = 29 person-months of follow-up
- A total of 29/12 = 2.42 person-years of

follow-up Summing up the person-years of follow-up

in column 4 gives

- **A total of 104.83 person-years from the start of the trial to the end of March 2018.**

Years of follow-up from the start of April 2018 to the end of July 2019

Table 2 shows projected recruitment into the trial from the start of April 2018. After July 2019 patient entry is uniform at 135 per month and the period up to this is considered here. At the start of April 2018 there were 220 patients in the trial (discussed in the previous section). At the end of July the following year 1669 patients are estimated to have been entered, giving 1669 – 220 or

- **A total of 1449 patients recruited from start of April 2018 to end July 2019**
- **A total of 1669 patients in total by end July 2019**

The potential months of follow-up for each of the newly recruited patients up to end July 2019 are shown in column 5 and column 6 gives the person-years of follow-up for all patients. Summing up this column gives

- **A total of 775.96 person-years of follow-up for the newly recruited patients from start of April 2018 to end July 2019**

Additionally, in this 16 month period the 220 patients already entered had a total of 16 x 220 = 3520 person-months of follow-up which is 293.33 person-years.

- **293.33 person-years of follow-up from start of April 2018 to end July 2019 for the 220 patients who entered the trial before April 2018**

Years of follow-up from the start of August 2019 to the end of June 2020

At the start of August 2019 there is an estimated 1669 patients entered (including the 220 entered prior to April 2018) . Up to the end of June 2020 (11 months) the person-years of follow-up for these patients would be (1669 x 11)/12 = 1529.92

- **The person years of follow-up in all 1669 patients entered before start of August 2019 up to end June 2020 is 1529.92**

In this 11 month period 135 patients per month are estimated to enter the trial. This is a total of 1485 patients. They have each an average follow-up of 11/2 = 5.5 months giving (5.5 x 1485)/12 = 680.63 person-years of follow-up.

- **In the 1485 recruits, from start of August 2019 to end June 2020, there would be 680.63 person-years of follow-up.**
- **A total of 3154 patients are estimated to enter the trial before the end of June 2020**

Follow-up from start July 2020 to end September 2022

The follow-up of all 3154 subjects from the start of July 2020 will add (3154 x 27 months)/12 = 7096.5 potential person-years of follow-up to the study.

- **7096.5 person-years of follow-up will be accrued in the follow-up period from start July 2020 to end September 2022.**

Events

This section estimates the number of events expected in CONVINCE and shows that the study requires the 3,154 patients projected to be recruited by the end of June 2020, together with a further 27 months follow-up of all 3,154.

Number of events up to end September 2022

The hazard rate in the treatment group (adjusted for dropouts) is 0.03608, giving 36.08 events per 1,000 person-years. In the control group it is 0.04834 giving 48.34 events per 1,000 person-years. Since there will be the same number of patients and the same number of person-years in each of the two groups the hazard rate in the two groups combined is the average of the component hazards or (0.04834 + 0.03608)/2 = 0.04221. This gives 42.21 events per 1,000 person-years. Table 4 below shows the number of events in the different follow-up periods. The number of events however is biased upwards because no account is taken of the effect of an event on subsequent person years of follow-up.

Table 4 Calculation of events

Period concluding at end of	Total recruited in period	Person-years of follow-up in period		Total person-years of follow-up in period	Events expected in period *	%
		New recruits	Earlier recruits			
March 2018	220	104.83	-	104.83	4.42	1%
July 2019	1449	775.96	293.33	1069.29	45.13	10.2%
June. 2020	1485	680.63	1529.92	2210.54	93.31	21.1%
Sept. 2022	0	-	7096.5	7096.5	299.54	67.71%
**Total**	**3559**			**10481.16**	**442.4**	**100.0 %**

*** Column 3 x 0.04221**

The total number of events expected in the trial, given the recruitment projections, is 442 and 68% of them occur in the period June 2020 to September 2022 showing the value and necessity of this two-year period of follow-up for the trial.

Adjustment for reduced person-years of follow-up in those who had an event

It has already been noted that the person-years of follow-up used so far in these calculations are potential periods of follow-up which ignore the effect of a death which precludes further follow-up. In fact it is assumed in what follows that there is no period at risk in a subject following the occurrence of their first event even if it is not fatal[5]. The average person-years of follow-up in the trial is the total person-years divided by the no of subjects which is 10,481.16/3154 = 3.32 years.

If follow-up times are distributed equally between those who have an event and those who are event free, this average in particular applies to the group of those who experience an event. If events are distributed uniformly over follow-up (which arises from the hazard being time- independent) then, on average, events occur half-way into the potential follow-up which is at 3.32/2 = 1.66 years. Each subject who experiences an event loses 1.66 years of his/her potential follow-up time. Thus the calculated total person-years of follow-up must be reduced by 1.66 x 423.53 = 703.1 person years. This corresponds to a reduction in the total number of events by 0.04221 x 703.1 = 29.7 events. Thus the number of events in the study is 442.4 – 29.7 = 412.7 events.

Conclusion and discussion

The required number of events is 367 . Thus it can be concluded that the trial with a total sample size of 3154 and follow-up as projected achieves over 80% power (actually 84.5%) to detect a 26% reduction in the event rate (13.5% to 9.99% and allowing for 15% dropouts) at 5% two-sided significance. Analysis will be using Cox proportional hazard regression to adjust for specified confounders, including minimisation factors applied at randomisation.

This sample size estimation is based on the accuracy of the assumptions made. The most important of these are noted below

- A hazard that is constant over time in both the treatment and control groups. This is a very strong assumption and is much more stringent than the proportional hazards assumption which is implied by this assumption anyway.
- Applicability of using the total events required determined from a sample size program with different assumptions

[5] In the trial subjects are followed up after a non-fatal event so that other outcomes can be identified. However for the primary analysis the endpoint is the first occurrence of one of the defined events and subsequent events are not counted so the follow-up does not constitute a risk period.

- Appropriateness of the first calculation of the number of events by multiplying the annual hazard by the potential person-years of follow-up
- Validity of the heuristic adjustment of the number of events for the reduced follow-up due to the occurrence of events.
- Validity of assumptions that deaths and dropouts occur on average halfway through their potential follow-up

The trial statisticians believe that the projected numbers for the CONVINCE trial will result in an ability to detect the treatment effect postulated

APPENDIX 14: DEFINITIONS OF TARGETED ADVERSE EVENTS

System	Adverse event	Definition/comment
Blood and Lymphatic System	Bone marrow depression with agranulocytosis, aplastic anemia, thrombocytopenia	Defined as Hb<10mg/dL, in the absence of major bleeding; neutrophil count <1.5 x 10^9/L; platelet count <75 x10^9/L (any one of these)
Nervous System	New Peripheral Neuropathy	Defined as new or worsened symptoms of numbness, parasthesia, burning or weakness in the extremities *with confirmation on nerve conduction studies*
GI System	Abdominal pain, Nausea/Vomiting, Diarrhoea	
Liver	Hepatic Damage	Transaminases >2ULN (ALT, AST, or both)
Skin and Subcutaneous Tissue	Alopecia, Rash	
Musculoskeletal	Myopathy/myalgias (muscle weakness/pain + raised CK), rhabdomyolysis	CK must be >2 ULN
Renal/urinary system	New Acute or Chronic Renal Injury	Chronic: eGFR less than 50ml/min/1.73m^2 on two measurements at least 3 months apart<br>Acute: Increase in Serum Creatinine to X1.5 times baseline, which occurred within the prior 7 days
Reproductive system	Oligospermia, azoospermia	
Metabolic	Low serum B12	Defined as B12 level < 200ng/L
Infections	Upper Respiratory Tract Infection<br>Pneumonia<br>Urinary Tract Infection<br>Other serious infections	
Malignancy	All new malignancies	
Major Haemorrhage	All new major bleeds	Defined as:<br>1. Fatal bleeding.<br>and/or<br>2. Symptomatic bleeding in a critical area or organ, such as intracranial, intraspinal, intraocular, retroperitoneal, intra-articular or pericardial, or intramuscular with compartment syndrome.<br>and/or<br>3. Bleeding causing a fall in hemoglobin level of 2 g/dL or more, or leading to transfusion of two or more units of whole blood or red cells.

Appendix 15: UPDATED CONVINCE PROCEDURES IN RESPONSE TO COVID-19

Background:

Together with the trial Sponsor, Steering Committee members, and key support teams, procedures for the CONVINCE trial have been reviewed in response to the COVID-19 pandemic in the context of guidance from national regulators. Based on a risk-benefit assessment, to protect the health of patients who may be at risk of contracting COVID-19 from non-essential contacts with research staff, and equally to protect the research staff who may be at risk of infection during research encounters, several modifications to trial procedures have been made.

1. Recruitment of new patients – Remote Screening/Baseline Visits:

Most patients will be recruited as outlined in the protocol. ***This remains the preferred option.*** Some patients meet all other eligibility criteria but are rapidly discharged from hospital according to new COVID-19 pathways before 72 hours have passed since the Qualifying Event (stroke or high-risk TIA).

In this situation, as an alternative option, the following procedure may be applied:

1. informed consent is obtained from the patient before hospital discharge. The Screening/Baseline visit may be done at this time, before hospital discharge or may be done by phone after discharge.
2. As soon as possible after the 72 hour period has passed, the patient should be contacted by phone
3. The site team must verify that the patient is still eligible for the trial. If the Screening/Baseline visit has not been completed before discharge, it can be completed during the phone call.
4. Randomisation is done during the phone call
5. If randomised to colchicine, study drug is provided to the patient as outlined below and the usual instructions provided for storing and taking colchicine
6. The Screening/Baseline Form in the eCRF is completed as usual.
7. The MOCA need not be done. This is not classified as a Protocol Violation. The EQ5D may be done by phone (it is validated for phone use)

Summary: Recruitment of new patients:

**A: Preferred Option**	**Consent, Screening/Baseline Visit, Randomisation and Study Drug dispensing procedures all done at face-to-face visit, as per original pre-COVID protocol**
**B. Alternative Option permitted**	**For patients discharged early due to COVID pathways:**<br>**Consent done and Screening/Baseline started at face-to-face visit. Screening/Baseline, Randomisation, and Drug dispensing finished remotely post-discharge after 72 hours**

2. Follow-up visits - In-person and Remote:

In response to the COVID-19 pandemic, to reduce unnecessary visits to hospital, the following procedures will apply:

1. Timing of follow-up visits: All follow up visits should be completed as close as possible to the schedule outlined in the study protocol. Increased flexibility is permitted for the timing of the follow-up visit, if judged by the site team that this is a practical solution to completing the visit. If the visit is conducted outside the range +/-7 days of the scheduled date, this is not classified as a Protocol Violation.

2 The protocol currently allows for follow-up visits to be completed by telephone with patient supported by a family member if needed. **Remote visits may also be done by video-call** (using Skype, Zoom, or other applications).

3 The site team will verify patient compliance with study medication by counting the number of remaining tablets during the telephone/video-call. It is acceptable for the patient to return unused medication via standard post to the study office, using a stamped addressed envelope provided by the study team. Alternative methods of return of unused medication (eg. delivery by patient's family to a study team member at the hospital door) are acceptable, if considered feasible in the opinion of the study team. Other procedures for drug accountability are unchanged.

4. Pulse and blood pressure need not be measured at remote follow up visits. This is not a Protocol Violation. Home measurements of blood pressure and/or pulse taken by the patient are acceptable.

5. The modified Rankin Score can be scored remotely, using the standard mRS algorithm (Protocol, Appendix 1)

6. Laboratory tests are no longer mandatory at Follow-up visit 1 (28 days), if this visit is done remotely.

7. For remote follow-up visits, the screening strategy for laboratory abnormalities is changed to opportunistic screening instead of scheduled screening. This means that ***blood tests for scheduled monitoring of bone marrow suppression, hepatic injury, renal failure, B12 or CRP are no longer mandatory at annual follow-up visits if such visit is done remotely*** (justification for this change is provided below). Investigators should do these tests at the next in-person visit. If an in-person visit is not scheduled, it is acceptable to record results of these blood tests once per year if the patient has them done during a hospital visit for routine clinical care, with the date of the test. If the blood tests are not done within one year, non-completion of laboratory tests will not be classified as a Protocol Violation.

8 Where possible, the End of Trial/Withdrawal Visit should be done as a face-to-face visit. Laboratory tests should be done at this visit where possible. If a face-to-face visit is not possible, it is acceptable for this visit to be done remotely without laboratory tests. In this case, a remote visit or unavailability of laboratory tests will not be classified as a Protocol Violation.

9 Questionnaires: If the 2-year and End of Trial/Withdrawal visits are done remotely, the MOCA need not be done. This is not classified as a Protocol Violation. The EQ5D may be done by phone

Summary: Follow-up visits:

**A: Preferred Option**	**Follow-up at face-to-face visit**
**B. Alternative Option permitted**	**Follow-up at remote visit by phone or video-call**

Summary: Blood tests at follow-up:

**A: Preferred Option**	**Blood tests done at 28 days and at follow-up visits at 1,2,3 years and end of trial**
**B. Alternative Option**	**If blood tests are not available at times above, they can be recorded from routine clinical care or a later follow-up study visit. If blood tests not available, no protocol violation**

3. Resupply of existing patients on colchicine:

A priority will be to ensure continuous supply of colchicine to patients in this arm of the trial. A range of options are available to ensure this:

a) Colchicine may be dispensed at a scheduled in-person visit, per the main protocol

b) In areas where clinic visits are continuing, **patients may be called in early by site teams** to be re-supplied with colchicine. If needed, the remainder of the follow-up visit may be done by phone after colchicine is dispensed.

c) **Drive-through dispensing is allowed**. The patient or family member drives to the outside of the research building, and site team personnel hand over study medication and receive returned packets and unused tablets. The remainder of the follow-up visit is done by phone.

d) If patient visit or drive-through dispensing are not possible, some sites may use **delivery of colchicine to patients**. Usually the site must have an established relationship/contract with a courier company (or similar service) to avail of this option. Costs will be reimbursed and logistics supported by the trial Project Office, where practical. Guidelines for same will be provided.

If a temporary interruption of study colchicine occurs, study medication should be re-started as soon as possible in this situation. A Protocol Deviation form should be completed, and the Project Office notified of the interruption.

Summary: Colchicine dispensing:

**A: Preferred Option**	**Dispensing at face-to-face visit as per pre-COVID protocol**
**B. Alternative Option permitted**	**Drive-through dispensing**
**C. Alternative Option permitted – Last resort**	**Delivery of colchicine to patient**

4. Safety Reporting of cases of COVID-19:

All cases of COVID-19 identified in trial subjects should be reported as Serious Adverse Events, regardless of whether the patient is in the colchicine or usual care arm, and even if the symptoms are mild. As for all SAEs, these should be reported within 24 hours to **sae.reporting@ucd.ie** using the form in Section 15 of the site file, and the CRF Adverse Event page should be completed.

Justification of opportunistic screening for laboratory abnormalities in patients undergoing remote visits:

In a 2016 Cochrane review, no increase in Serious Adverse Events (SAEs) related to bone marrow suppression, renal or hepatic injury, or low B12 was identified (Hemkens, Cochrane database, 2016). However, in CONVINCE prior to COVID-19, the protocol required annual screening of these systems for enhanced safety monitoring in all patients. Since the protocol was written, new data suggests that significant toxicity of colchicine related to these body systems is unlikely. In 4745 patients in the COLCOT trial (New England Journal of Medicine, 2019), no such safety concerns were identified. Similarly, in 5522 patients in the LoDoCo2 trial, no increase in toxicity related to these body systems was identified. In the first 964 patients recruited in CONVINCE, the incidence of myelosuppression, renal or hepatic injury and low vitamin B12 was similar between groups. Based on a risk/benefit assessment, taking into account the potential risk to study subjects of COVID-19 exposure/infection associated with hospital visits for routine blood tests, the strategy has been modified to opportunistic screening for laboratory abnormalities in patients undergoing remote visits, and study procedures have been modified.
