# Supplementary material for: Validation of the SMART-REACH model after stroke and the effect of colchicine by atherosclerotic cardiovascular disease risk category: a secondary analysis of the CONVINCE randomised clinical trial
Source: Eur Stroke J. 2026 Apr 25;11(4):aakag033. doi: 10.1093/esj/aakag033 (PMC13109098; doi:10.1093/esj/aakag033)
Supplement: Supplemental_Materials_aakag033 [file supplemental_materials_aakag033.zip › Supplemental_Materials_aakag033_NEW/Supplemental Material - Table S1.docx]

| **Table S1: SMART-REACH predictor variables by risk category and treatment assignment** | | | | | | | | | |
| --- | --- | --- | --- | --- | --- | --- | --- | --- | --- |
|  | Moderate (10-19%) risk | | | High (20-29%) risk | | | Very high (≥30%) risk | | |
|  | Total  (n = 594) | Colchicine  (n = 304) | Control  (n = 290) | Total  (n = 1148) | Colchicine  (n = 568) | Control  (n = 580) | Total  (n = 1401) | Colchicine  (n = 697) | Control  (n = 704) |
| Female | 193 (32.5) | 99 (32.6) | 94 (32.4) | 355 (30.9) | 185 (32.6) | 170 (29.3) | 404 (28.8) | 204 (29.3) | 200 (28.4) |
| Current smoking | 65 (10.9) | 33 (10.9) | 32 (11.0) | 282 (24.6) | 139 (24.5) | 143 (24.7) | 347 (24.8) | 178 (25.5) | 169 (24.0) |
| Diabetes | 9 (1.5) | 8 (2.6) | 1 (0.3) | 163 (14.2) | 86 (15.1) | 77 (13.3) | 529 (37.8) | 264 (37.9) | 265 (37.6) |
| Systolic Blood Pressure, mmHg | 138 (22) | 138 (23) | 137.5 (21) | 136 (23) | 136 (23) | 136 (23) | 137 (25) | 137 (25) | 138 (25) |
| Total cholesterol, mmol/L | 5 (1.5) | 5 (1.6) | 5.1 (1.3) | 4.8 (1.7) | 4.8 (1.7) | 4.8 (1.6) | 4.3 (1.9) | 4.3 (1.9) | 4.3 (2) |
| Creatinine, µmol/L | 76 (20) | 76 (21.2) | 75 (19) | 76 (21) | 76 (20.2) | 76 (22) | 82 (26) | 81 (26) | 83 (25.2) |
| Number of cardiovascular disease locations  Coronary artery disease  Cerebrovascular disease  Peripheral artery disease | 6 (1.0)  594 (100)  2 (0.3) | 3 (1.0)  304 (100)  0 (0) | 3 (1.0)  290 (100)  2 (0.7) | 57 (5.0)  1148 (100)  16 (1.4) | 19 (3.3)  568 (100)  8 (1.4) | 38 (6.6)  580 (100)  8 (1.4) | 303 (21.6)  1401 (100)  110 (7.9) | 142 (20.4)  697 (100)  55 (7.9) | 161 (22.9)  704 (100)  55 (7.8) |
| Atrial Fibrillation | 0 (0) | 0 (0) | 0 (0) | 0 (0) | 0 (0) | 0 (0) | 0 (0) | 0 (0) | 0 (0) |
| Heart Failure | 0 (0) | 0 (0) | 0 (0) | 3 (0.3) | 2 (0.4) | 1 (0.2) | 51 (3.6) | 25 (3.6) | 26 (3.7) |
| Antithrombotic therapy  None  Antiplatelet monotherapy  Dual antiplatelet therapy  Anticoagulant | 4 (0.7)  243 (40.9)  343 (57.7)  4 (0.7) | 1 (0.3)  103 (33.9)  199 (65.5)  1 (0.3) | 3 (1.0)  140 (48.3)  144 (49.7)  3 (1.0) | 27 (2.4)  605 (52.7)  508 (44.3)  8 (0.7) | 15 (2.6)  299 (52.6)  251 (44.2)  3 (0.5) | 12 (2.1)  306 (52.8)  257 (44.3)  5 (0.9) | 61 (4.4)  887 (63.3)  439 (31.3)  14 (1.0) | 38 (5.5)  433 (62.1)  222 (31.9)  4 (0.6) | 23 (3.3)  454 (64.5)  217 (30.8)  10 (1.4) |
| *Continuous variables are presented as median (interquartile range), categorical variables as N (%).* | | | | | | | | | |
